# Novel Haptic Electronic Yarn for Wearable Textiles: Vibrotactile Feedback via Knitted Channels for Skin-Based Communication

**DOI:** 10.1038/s41528-026-00613-w

**Published:** 2026-07-04

**Authors:** Malindu Ehelagasthenna, Arash Moghaddassian Shahidi, Pasindu Lugoda, Parvin Ebrahimi, Carlos Oliveira, Dominic Eberl-Craske, William Hurley, Tilak Dias, Theodore Hughes-Riley

**Affiliations:** 1 Nottingham Trent University, Nottingham School of Art & Design, Nottingham, United Kingdom; 2 University of California, Davis, CA USA; 3 University College London, Department of Orthopaedics and Musculoskeletal Science, Division of Surgery & Interventional Science, Brockley Hill, Stanmore, United Kingdom; 4 Nottingham Trent University, School of Engineering, Nottingham Trent University, Clifton, Nottingham United Kingdom

**Keywords:** Engineering, Materials science

## Abstract

This work introduces a novel electronic yarn (E-yarn) capable of delivering controlled vibrotactile feedback, offering a seamless haptic integration technique into wearable textiles. By embedding miniature vibrotactile motors at the yarn level and employing robust encapsulation techniques, the design ensures durability and compatibility with everyday wear. Prototypes, including a knitted glove incorporating these haptic E-yarns, demonstrated sustained functionality over 20 wash cycles with minimal resistance change (< 1%), and first signs of failure at cycle 23. Performance evaluation using a custom test rig revealed strong correlations between compression forces and vibrotactile output, with frequency and amplitude influenced by textile structure and dynamic loading. Haptic E-yarns achieved an average vibration of 282.6 ± 33.4 Hz at 0.34 ± 0.2 ms^−2^, with only 1–5% frequency variation and up to 40% amplitude increase post-integration to wearables. This technology enables reliable haptic feedback within textiles, paving the way for future applications in assistive communication and interactive wearables.

## Introduction

Haptic technology has been widely explored in human-machine interaction, offering a communication method beyond visual and auditory cues. In recent years, the advancement of haptic technology in wearable devices has further expanded its applications, enhancing user experiences in immersive gaming, interactive education, rehabilitation^1–4^, medical diagnostics^5^, therapy^6^, and communication^7–12^. In the context of communication, haptic technology enables users to express emotions and convey information through tactile sensations, effectively bridging the gap between physical and digital interactions^7^. Textiles present a particularly promising platform for this purpose, as their continuous and intimate contact with the skin allows for seamless, accessible, and intuitive interaction. Building on this potential, the present study focuses on designing and developing an electronic yarn capable of delivering haptic feedback, thereby enabling tactile communication within textile-based substrates.

Haptics arise from two primary nerve functions: tactile sensing and kinesthetic sensing^13,14^. Tactile sensing detects vibrations, skin indentation, and skin stretch through mechanoreceptors^13,15,16^, while kinesthetic sensing perceives internal forces and movements in muscles, tendons, and joints^13,16^. This study focuses primarily on vibrotactile feedback, which stimulates mechanoreceptors that respond to vibrations in the range of 0.4 Hz to 1000 Hz, with peak sensitivity between 200 Hz and 300 Hz^13,17^.

Recent research has explored various mechanisms that provide tactile feedback, including vibration, point-based pressure, compression, and skin stretch^18–23^. Among them, vibrotactile stimulation is commonly used in integrated textile technologies due to its effectiveness and adaptability^24^. Several actuation technologies generate vibrotactile feedback, such as piezoelectric^19,25^, dielectric elastomer^26–31^, electrotactile^32–35^, fluidic^36–38^, and electromagnetic actuators^17,39,40^. However, each technology presents trade-offs in terms of flexibility, power consumption, safety, and integration into wearable textiles (see Table 1). Among electromagnetic actuation technologies, eccentric rotating mass (ERM) motors and linear resonant actuators (LRAs) are the most prevalent. In particular, ERM motors are commonly employed in commercial haptic wearables such as the bHaptics TactSuit and TactGlove^39,40^. These actuators are compact, lightweight and operate efficiently at low DC voltages (2.5–3.0 V, 27–78 mA)^17^, delivering vibration feedback within the range of human tactile sensitivity^17^. While LRAs offer improved durability, ERM motors typically generate stronger maximum vibration amplitudes (2.5–30.0 ms^−2^) compared to LRAs (0.7–1.9 ms^−2^)^17^. Furthermore, LRAs require an AC power source (1.2–2.0 V AC, 28–69 mA), adding complexity to power management. In contrast, ERM motors are powered by a simple DC supply, which makes them more suitable for integration into compact, energy-efficient wearable systems^17^. Despite these advantages, their successful integration into textiles presents engineering challenges related to comfort, breathability, washability, and flexibility^41^.

**Table 1:** Technical comparison of textile-integrated vibrotactile haptic actuators

Actuator	Dimensions	Weight	Voltage	Current	Integration	Primary	Critical
Category	(mm)	(g)	(V)	(mA/A)	Methodology	Advantages	Limitations
Pneumatic^87,88^ (Thin-Film)	25 (Ø) × 0.4 (t)	< 2.0 g	N/A (Fluidic)	N/A (Fluidic)	Heat-pressing	High force/weight; conformable	External pumps/valves noise and bulk
Pneumatic^89,90^ (Bladder)	> 4.5 (Ø) (Knit area)	> 3.0 g (Fabric)	N/A (Fluidic)	N/A (Fluidic)	Machine knit; yarns	Radial forces; radial displacement	Force loss if fabric lacks stiffness
SMA^24,91–93^	0.20–0.51 (wire Ø)	Minimal (Wire)	9.4 V	0.4–1.0 A	Knitting SMA or smocking folds	Silent; very high power density	Slow cooling limits frequency
FFMS^94,95^	Tubes: 1.0 (Ø)	6–40 g (System)	1.4– 1.5 kV	< 1 mA	Stitching elastic tubes in fabric	Near-lossless force; high stroke	Risk of leakage; liquid reservoir
DEA^24,96,97^ (Elastomer)	18 *μ*m (t)	0.09– 1.3 g	450– 3500 V	0.1 mA	Lamination or active yarns	Fast response; large strain	High voltage safety risk; complex drive
Electro- tactile^91,96,98,99^	2–5 Ø (electrode)	Minimal (Film)	10–50 V	1–13.5 mA	Electrospinning; conductive yarns	No moving parts; high density	Tingling feel; varying skin impedance
Electro- magnetic^17,98,100^	6.0–18.0 (Ø)	0.95– 3.3 g	1.2– 5.0 V	5–250 mA	Pocketing or weaving fibers	Simple control; low latency	Rigid magnets limit textile drape
This work	5.3 ± 0.2 Ø 20 (L)	2.5 g (300 mm)	2.5-3.6 V	< 90 mA	Insert through knitted channels	Textile feel; washability	Stiffness of ERM motor

Addressing these challenges, this study explores an innovative approach by embedding miniature vibrotactile motors directly into textile yarns to create haptic electronic yarns. These yarns can be seamlessly integrated into knitted fabrics, forming textile panels with haptic feedback. Knitting offers superior flexibility, elasticity, and breathability compared to other textile fabrication methods, making it ideal for wearable integration^42^. This research investigates encapsulation techniques for ERM vibrotactile actuators, mechanical reinforcement strategies, and textile covers to optimize durability and tactile effectiveness. This work advances the current understanding of how mechanical forces are transmitted through fibres and textiles, building on prior studies by this group^43^ and others^44,45^, and extending these insights beyond the effects of knit architecture alone. There has also been significant interest in the literature exploring the use of fibre structures to specifically enhancing the transfer of mechanical forces for both sensing and actuation. For example, twisting individual fibres or fibre bundles has been shown to significantly enhance sensitivity by increasing deformation-induced contact changes, as demonstrated^44^. Likewise, wrinkled or buckled conductive surfaces on fibres have been shown to improve mechanical-electrical coupling and robustness in stretchable sensing textiles^45^. These structural strategies complement knitted architectures and highlight additional routes for sensitivity enhancement.

Furthermore, this study presents a functional prototype of a wearable haptic electronic glove designed to assist individuals with deafblindness, demonstrating the real-world impact and feasibility of engineered haptic E-yarn technology. Building on our previously published work^46,47^, the device could potentially leverage real-time haptic communication, navigation support, and alert mechanisms to enhance accessibility, independence, and overall quality of life. The findings of this research advance the field of haptic wearable technology by introducing a novel approach to integrate vibrotactile feedback seamlessly into everyday textiles.

## Results

### Development of Haptic Electronic Yarns for Haptic-Enabled E-textiles

**Fig. 1:**
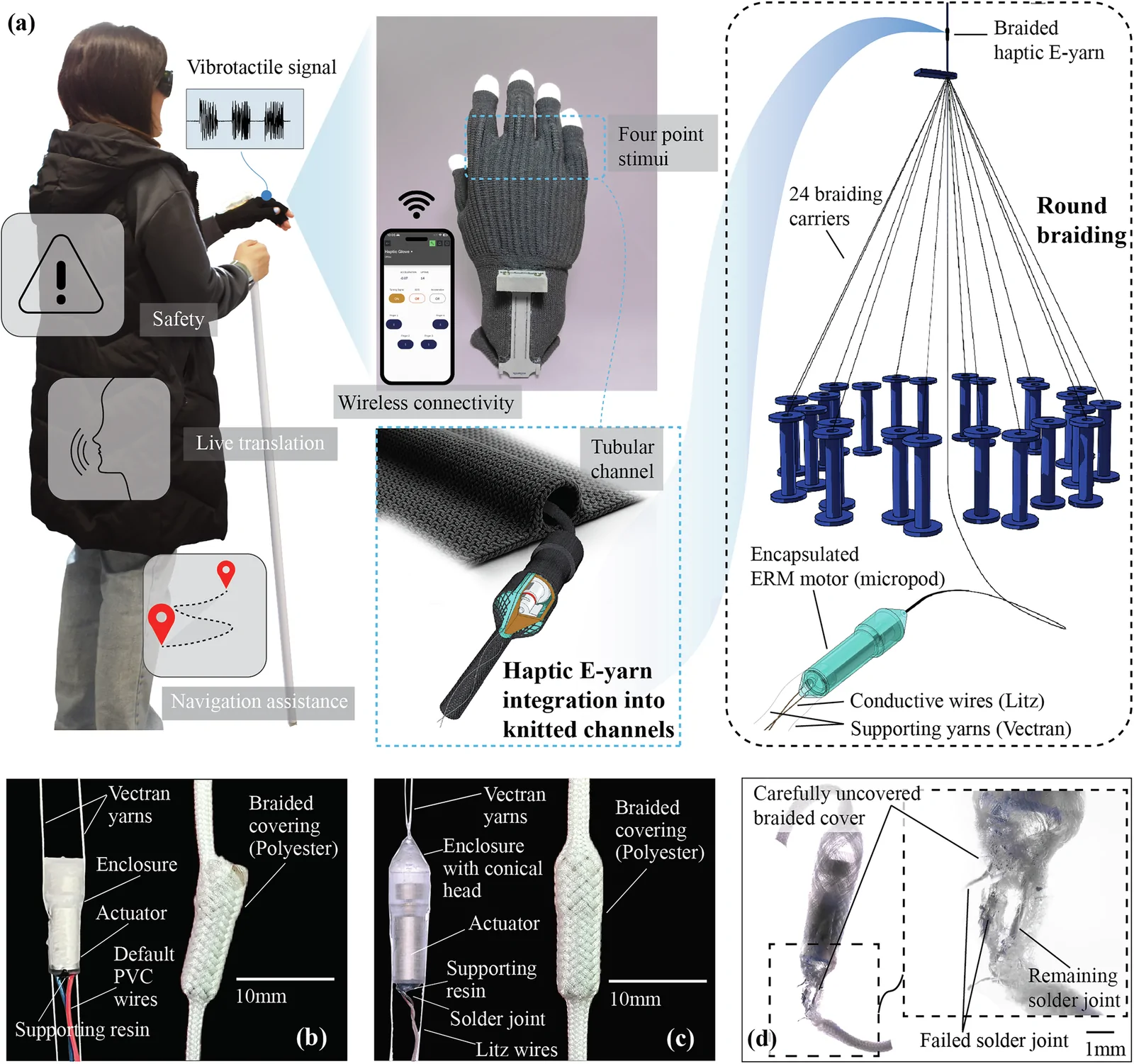
Development of haptic electronic yarns and applications. **a** Detailed schematic illustrations of a prototype haptic electronic glove developed to improve safety, assist live translation, and navigation for individuals with deaf-blindness. **b** Improved encapsulation and textile covering of the actuator with its default terminal wires. **c** Encapsulation and textile covering (configuration 1) of the actuator that is soldered its two terminals to Litz wires. **d** Failure of the solder joint of configuration 1 design due to mechanical stresses.

Developed wearable textiles with haptic feedback incorporate electronic yarns (E-yarns) with embedded vibrotactile motors as shown in Fig. 1a. Integration of such actuators at the textile yarn level which involves encapsulating the electronics and covering the assembly using a textile braiding process was identified mainly two critical design issues. First, improper guidance of the braid over the encapsulated motor assembly (defined hereafter as the *micropod*) resulted in misalignment during fabrication. This caused the micropod to deviate from the central axis of the yarn, leading to uneven or incomplete textile coverage and potentially compromising both structural integrity and functional performance (Fig. 1b). To address this, a conical geometry was introduced in the 3D-printed enclosure design, which significantly improved alignment during braiding process (Fig. 1c). Hereafter, the term *enclosure* refers to the 3D-printed components that house the ERM motors.

Second, failures were observed in the conductive paths near micropod-wire interface, particularly in the solder joints, during initial testing and handling (Fig. 1d). These failures are consistent with prior reports attributing such issues to high stiffness gradients at the interfaces in electronic textiles^48,49^. To mitigate this, a case study was conducted to optimize the micropod-wire interface and reduce mechanical stress concentrations on the solder joint (see Supplementary Movie 1).

### Finite Element Analysis for the Solder Joint Failure

**Fig. 2:**
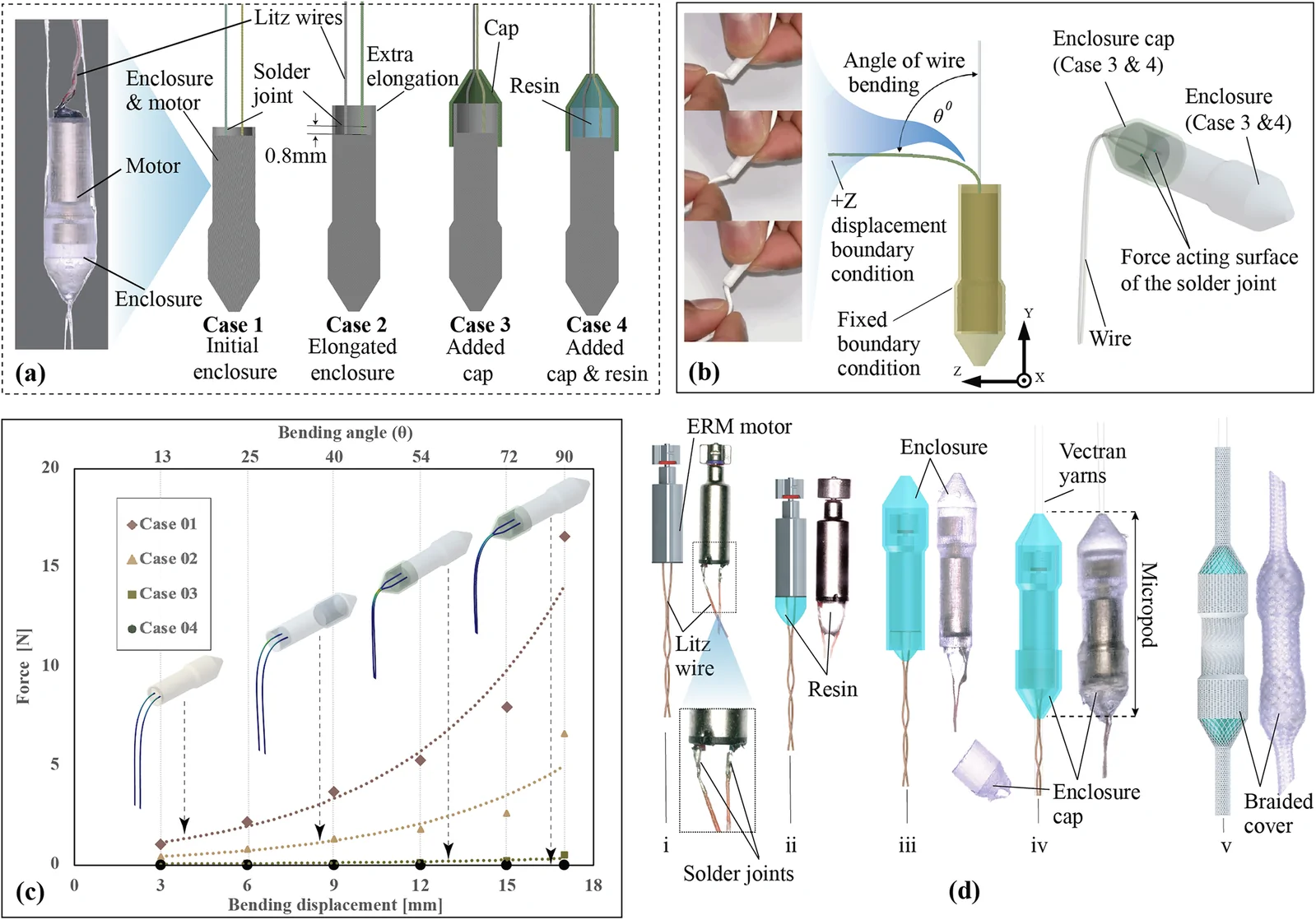
Numerical analysis of solder joint failure at the micropod-wire interface of haptic yarns under bending deformations. **a** Finite element analysis (FEA) conducted on four enclosure design modifications to enhance structural integrity and mitigate solder joint failure. **b** Boundary conditions were applied in the FEA simulations to accurately replicate real-world bending deformation. **c** Visualization of force distribution on the solder joint under different bending angles as simulated in the FEA model. **d** Illustrations of optimized enclosure configurations and fabrication steps of the haptic E-yarn.

The case study was performed using numerical simulations to estimate forces acting on solder joints and conductive wires under bending deformation at the micropod-wire interface. Four conceptual encapsulation configurations were developed iteratively to minimize the force acting on the solder joint, and each configuration was modelled using finite element analysis (FEA) to evaluate its mechanical performance (see Fig. 2a). The FEA models were simplified to reduce numerical errors and computational time. Specifically, the ERM motor and enclosure were modelled as a single body to reduce computational complexity (Fig. 2a).

The four encapsulation configurations are shown as four configurations in Fig. 2a. Configuration 1 reflected the improved enclosure design with conical head described earlier (Fig. 1c, d). Configuration 2 introduced a slightly elongated enclosure end to cover the solder joint (see case 2 in Fig. 2a). Configuration 3 added an enclosure cap to further reduce the deformation of the bending in the solder joint area, while configuration 4 incorporated additional resin inside the enclosure cap to minimize the high stiffness gradient at the micropod-wire interface (Fig. 2a). This reduction in stiffness gradient was achieved by using the resin with a hardness of 45D^50^, which is more stiff than the conductive Litz wire yet more flexible than the ERM motor (Fig. 2a).

Boundary conditions were defined to replicate real-world constraints on the elements of the haptic E-yarn. To simulate bending, the enclosure was fixed using ANSYS boundary conditions, and a displacement constraint was applied at the free end of the Litz wires in the +Z direction (free in Y, restricted in X), enabling bending up to *θ* = 90° (Fig. 2b). These conditions were applied consistently across all four configurations.

In configurations 1-3, additional fixed boundary was introduced at the enclosure-wire interface on the XZ plane to mimic restrictions imposed by the braided textile covering (Fig. 2a, b). In configuration 3, this region was shifted to the enclosure cap-wire interface. In configuration 4, the fixed region defined in configuration 3 was expanded to include the entire wire-resin contact area (highlighted in light blue in Fig. 2a), replicating resin-imposed constraints. These conditions restricted X and Z movements of the conductive wire at the specified interfaces while permitting free deformation along the Y-axis, thereby reproducing directional constraints imposed by the resin and braided textile cover. Mesh refinement was also performed to ensure accuracy, and detailed mesh parameters are provided in the Supplementary Information (see Supplementary Table 1).

As the ANSYS Parametric Design Language (APDL) commands were used to measure angular displacement of the conductive wire, a consistent 90° bend across all configurations were ensured (see Fig. 2c). Maximum stress of the conductive wire was evaluated at 0.8 mm from the ERM motor interface, corresponding to the solder joint position (Fig. 2a). Reaction forces at the motor-wire interface were also recorded to quantify the total force acting on the solder joint during bending. The numerically approximated maximum stress in the wire and solder joint, along with the maximum force acting on the solder joint, are summarized in Table 2. It should be noted that the wire stress values reported here are likely overestimates, as the simplified geometry and boundary conditions of the model do not capture the load-distributing effect of the multi-strand Litz wire architecture or the surrounding encapsulation material; these figures should therefore be interpreted as relative indicators of bending severity across configurations rather than absolute material stress predictions. FEA results indicated that although maximum stress on wire increased across consecutive configurations during bending (Table 2), maximum stresses and forces acting on solder joint decreased significantly by nearly 99.99% with successive design improvements (Fig. 2c). Configuration 4 reduced solder joint forces to near zero (Table 2; Fig. 2c). Therefore, configuration 4 was selected as the optimal design for its ability to minimize bending forces on solder joints, improve braid coverage, and enhance overall encapsulation integrity (Fig. 2d). This configuration has also been experimentally incorporated across multiple ERM motor models that vary in form factor, including differences in package geometry, mass, terminal configuration, solder-joint type (e.g., wire-lead versus pad-based terminations), and inter-pad spacing. Additional details are provided in the Supplementary Table 2 and 3.

**Table 2:** Numerical simulation results showing stress and force distributions on solder joints under 90^0^ wire bending across four configuration studies

Config.	Max. Stress	Max. stress	Max. force on the
	on the wire	on the solder joint	solder joint
	(MPa)	(MPa)	(N)
1	5,712	5,014.6	16.6
2	9,414.8	4,442.5	6.6
3	11,228	158.27	0.5
4	6092	≈ 0^†^	≈ 0^†^

^†^ Below numerical resolution of the FEA solver.

### Survival of Haptic E-yarns Under Bending Fatigue

A systematic bending fatigue study was conducted to experimentally validate the numerical predictions of the robustness of enhanced solder-joint under flexural deformation. Six haptic E-yarn specimens from the configuration 1 encapsulation design (*n* = 6) and six specimens from the improved configuration 4 encapsulation design (*n* = 6), each with an effective length of 300 mm, were subjected to controlled cyclic bending. As there was no specific universal standard for E-yarn bending tests at the time of this research, the bending test method was adapted from the *BS IEC 63203-204-2:2022, Wearable electronic devices and technologies — Part 204-2: Electronic textile — Test method to characterize the resistance change in the bending test of E-textile systems*^51^. A custom test apparatus was developed to replicate the deformation mode modelled in the FEA analysis, enabling repeated and consistent loading across all samples (see Supplementary Fig. 1 for technical details).

**Fig. 3:**
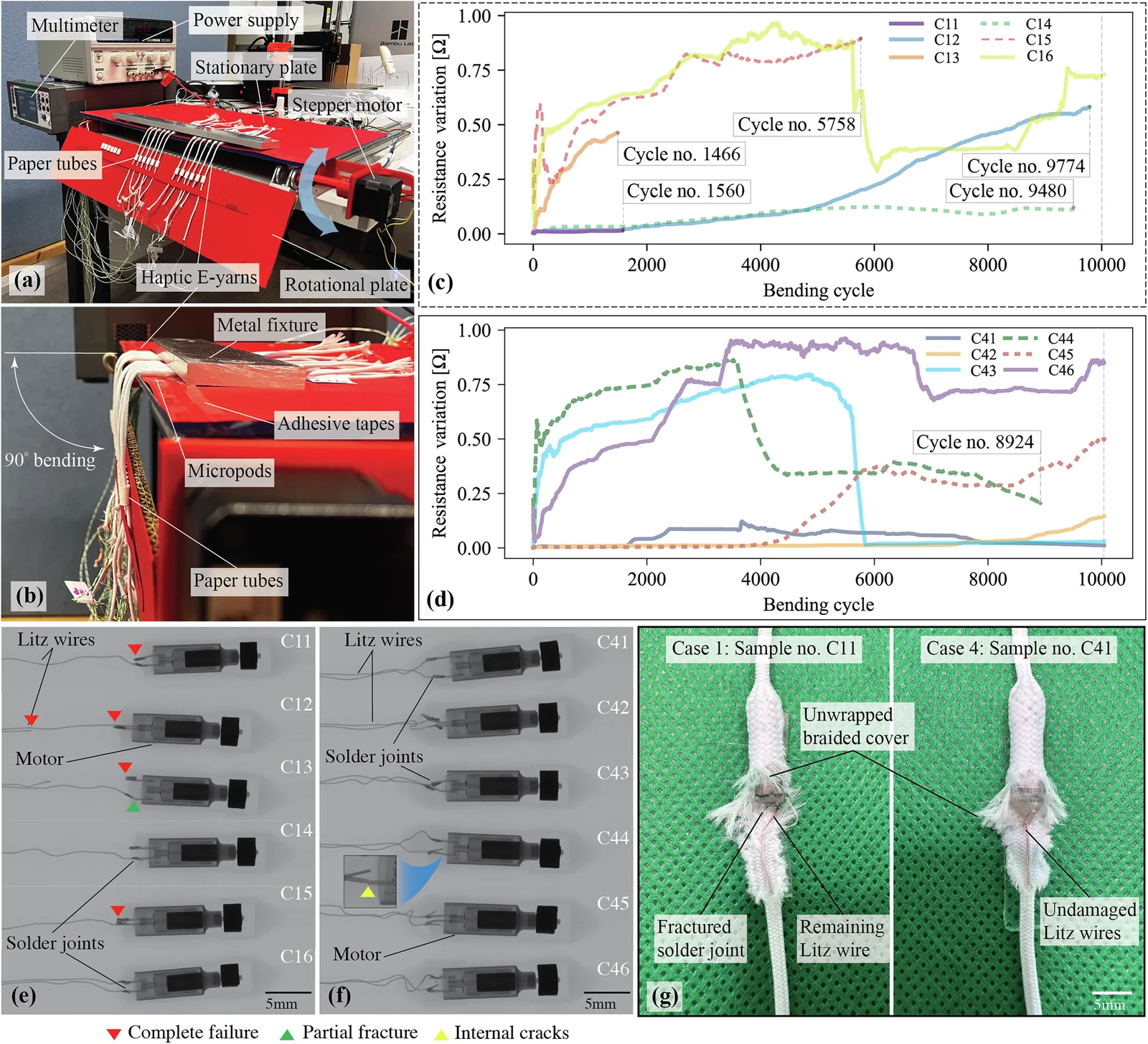
Repeated bending test at the micropod–wire interface of haptic E-yarns. **a** Custom test rig for repeated bending of haptic E-yarns with simultaneous resistance measurement. **b** Bending angle of 90° applied from the micropod-wire interface. **c**, **d** Moving-average of the absolute resistance variation over bending cycles for specimens with encapsulation designs of configuration 1 (C11-C16) and configuration 4 (C41-C46). **e, f** X-ray radiography of configuration 1 and configuration 4 specimens at the micropod-wire interface after test. **g** Unwrapped braided covers of representative samples from configuration 1 and configuration 4 after 10,000 cycles, showing solder-joint and Litz-wire integrity.

Each micropod of the specimens was securely mounted on double-sided adhesive tape beneath a metal clamping fixture. This mounting ensured that the deformation zone encompassed the micropod-wire interface, identical to the high-strain region analyzed in the numerical model (see Fig. 3a, b). The ends of each haptic E-yarn with Litz wires were guided through tubes to permit free longitudinal movement without clamping, thereby minimizing tensile loading and accurately reproducing the boundary conditions implemented in the FEA simulations (see Fig. 3b). All specimens were cyclically bent to a prescribed angle of 90° at a fixed frequency of 60 cpm for up to 10,000 cycles, which is the maximum number of cycles category recommended by BS IEC 63203-204-2:2022 (Category III ≥ 10,000)^51^.

The electrical resistance of each haptic E-yarn was continuously monitored throughout cyclic bending by using a DAQ6510 Multimeter System (Keithley, Tektronix Inc., Oregon, USA). Resistance was measured at the ends of the two Litz conductors in the haptic E-yarn, across a 150 mm conductive length, using a two-wire configuration. This resistance data was recorded at three stages: baseline (prior to cycling), in situ during cyclic deformation, and post-test after 10,000 cycles. Figure 3c, d show the absolute fluctuations in resistance for each specimen over the bending cycles, either up to the failure point or to 10,000 cycles. Configuration 1 specimens exhibited higher resistance fluctuations, with a mean variation of 0.34 ± 0.27 *Ω*, compared with 0.25 ± 0.22 *Ω* in the improved configuration 4 specimens.

Following 10,000 cycles, five of the six of configuration 1 specimens exhibited electrical failure, whereas only one specimen from configuration 4 failed. Figure 3c, d show the moving average of the absolute resistance fluctuations over the bending cycles for the tested haptic E-yarn specimens of configuration 1 and 4, respectively. Comparative X-ray radiography images shown in Fig. 3e, f acquired before and after testing, reveal detailed failure mechanisms, including failure locations, solder-joint cracking, breakage of individual coated copper strands that the Litz wire comprises of, and conductor discontinuities. All failures in configuration 1 originated at the solder-joint region, consistent with the high-strain concentration predicted by FEA (see Fig. 3e). A single failure observed in configuration 4 resulted from an internal discontinuity within the micropod, originating at a motor terminal rather than at the solder-joint or the micropod-wire interface of the Litz conductors (see Fig. 3f). This failure was likely caused by damage sustained during the haptic E-yarn fabrication process and subsequently propagated under cyclic bending. No deformation or strand-level damage was observed in any of the remaining configuration 4 specimens that include in regions adjacent to the micropod-wire interface compared to configuration 1 (see Fig. 3g). Furthermore, the average relative change in resistance after 10,000 bending cycles in configuration 4 specimens is 0.16 ± 0.18 Ω, revealing a stable electrical connection. These results provide direct experimental confirmation of the mechanical durability of the improved encapsulation design of the haptic E-yarns under dynamic bending fatigue and eventually validate the predictions of the numerical FEA study.

### Dynamic Response of the Haptic E-yarns

**Fig. 4:**
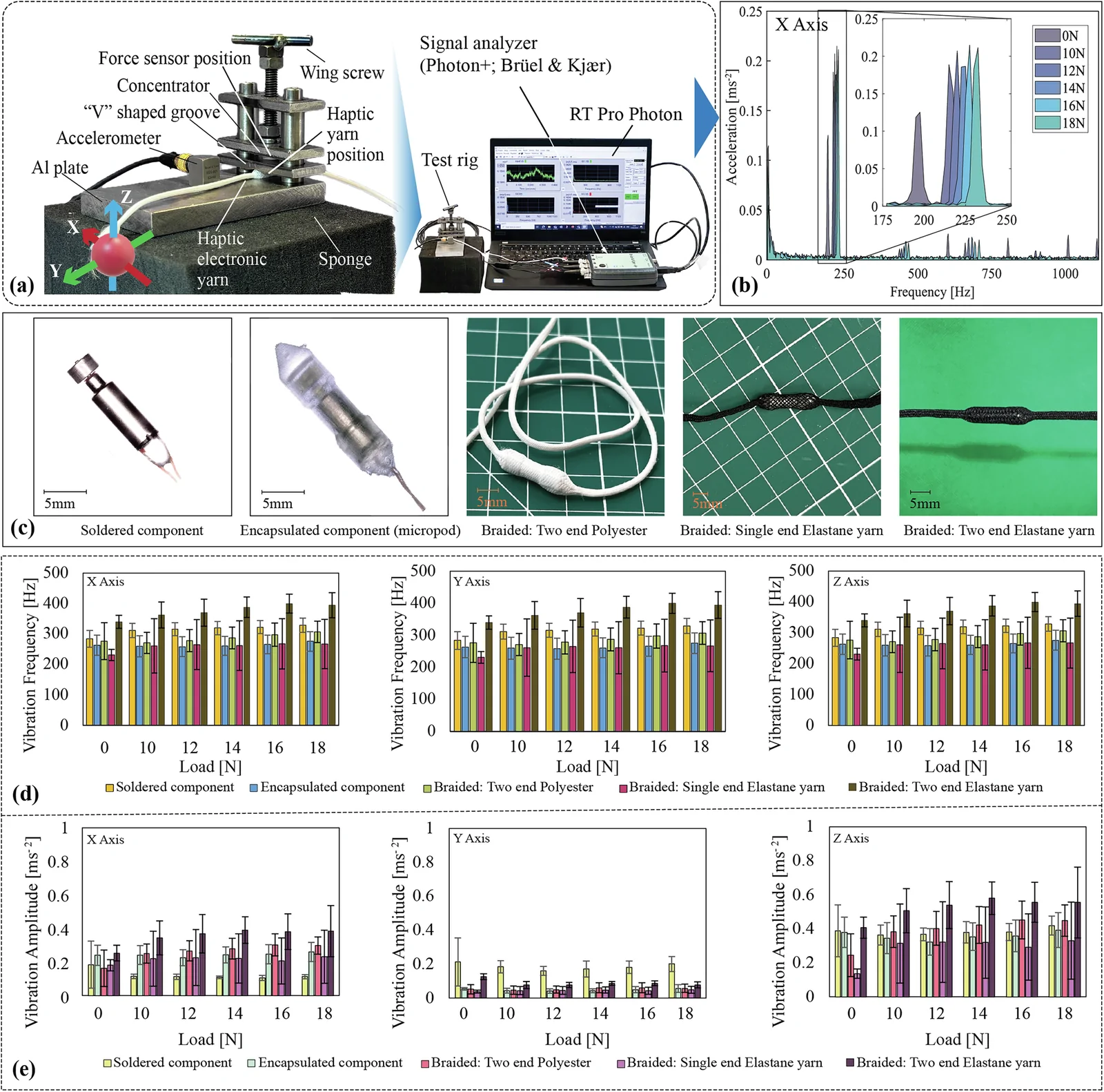
Experimental vibration response of the engineered haptic electronic yarn under continuous loading. **a** Developed test rig and vibration measurement setup for haptic E-yarns. **b** Vibration response along the X-axis under incremental loading, demonstrating an increase in frequency with rising load. **c** Analyzed E-yarn fabrication steps and braiding methods for vibration characterization. **d** Frequency response of the haptic E-yarn in all three axes (X, Y, Z) under progressive loading across five configurations: unsheathed soldered component, encapsulated component, final E-yarn braided with polyester, and final E-yarn braided with elastane. **e** Corresponding amplitude response in the X, Y, and Z axes under the same loading conditions and configurations, highlighting the influence of structural design on vibration behaviour.

The optimized haptic E-yarn design with improved encapsulation method was characterized using the dedicated testing rig developed to measure vibration feedback (see Fig. 4a). Given that vibration characteristics of ERM cylindrical motors vary under compression forces, a range of compressive loads (0-18 N) were selected. This range includes the practical loads previously reported for garments and gloves during dexterity- and posture-related compression^52,53^. In addition, typical forces encountered during daily activities and the threshold at which discomfort occurs are generally below approximately 9 N^54,55^. Therefore, extending the upper limit to 18 N ensured that the testing conditions exceeded normal physiological levels to enable the experimental performance with a safety factor. The selected range (0-18 N) also remained within the calibrated operating limits of the force sensor, avoiding measurement inaccuracies and preventing potential damage to both the actuator and the encapsulation.

Continuous loading caused a progressive increase in motor frequency and a corresponding rise in vibration amplitude, highlighting the yarn’s dynamic response under varying compressive forces (see Fig. 4b). As each haptic E-yarn fabrication step (soldering, encapsulation, and braiding) adds material that can also alter these loading conditions, a systematic characterization of the dynamic response with E-yarn fabrication steps was conducted using sets of haptic E-yarn samples (*n* = 6). Samples were tested at three successive fabrication stages: soldered component, encapsulated component, and braided component. Further, braiding materials and braiding parameters were systematically analyzed due to their influence on compression forces acting on the motors. Thus, two braiding materials were selected to examine their effect on vibrotactile feedback: high-strain elastane (20/DCY/003 Black, Stretchline, Sri Lanka; 44 Dtex Creora® elastane core, 44 Dtex/34 filament Nylon 66, 2 ply/78 Dtex/24 filament Nylon 6) and low-strain polyester (PE1716, J. H. Ashworth & Son Ltd, Kingston Mills, Hyde, Cheshire, United Kingdom; 1 ply/167 Dtex/48 filaments). With these selected two braiding materials and the effect of strand count in carrier yarns, three types of braid carrier yarns were evaluated: two-end polyester, single-end elastane, and two-end elastane (see Fig. 4c). These variations enabled analysis of braiding parameters, particularly material and number of strand count effects on vibration response. The influence of strand count in braiding carrier yarns revealed weaker textile coverage when single-end braiding carriers were used compared to two-end carriers (see Fig. 4c).

Each E-yarn fabrication step generally resulted in a reduction in vibration frequency under no-load conditions (0 N), except for braiding with two-end elastane yarn, which showed an increase (Fig. 4d). Although vibration frequencies were consistent across all three axes (X, Y, Z), amplitude behaviour (as a vector) varied due to movement restrictions imposed by the V-groove of the test rig (Fig. 4a). Notably, the Y-axis exhibited the lowest amplitude response, primarily because the motor’s rotational axis was aligned along the Y-axis during mounting on the test rig (Fig. 4a). However, braiding with two-end elastane yarn resulted in a clear increase in amplitude compared to other configurations.

Progressive compression loading (from 0 N to 18 N) on each configuration resulted in a notable increase in vibration frequency, while amplitude response showed mixed variations (both increases and decreases) depending on the configuration (Fig. 4d, e). Braided haptic E-yarns with two-end polyester braids exhibited a frequency of 276.6 ± 60.5 Hz and amplitude of 0.3 ± 0.2 ms^−2^ under no load, rising to 307.0 ± 35.5 Hz and 0.6 ± 0.1 ms^−2^ under an 18 N load. Single-end elastane braids showed 231.0 ± 18.6 Hz and 0.2 ms^−2^ without load, increasing to 267.0 ± 80.7 Hz and 0.4 ± 0.3 ms^−2^ under 18 N load. Two-end elastane braids achieved the highest values: 340.1 ± 21.1 Hz and 0.5 ms^−2^ without load, and 394.7 ± 41.3 Hz and 0.7 ± 0.3 ms^−2^ under 18 N load (Fig. 4c, d). Overall, increasing load consistently elevated both the average frequency and the resultant amplitude across all configurations.

These performance evaluations of haptic E-yarns with two different braided coverings (one less stretchable polyester and one highly stretchable elastane) showed that the elastane-based covering provided a 22.9% higher vibration frequency and a 66% higher amplitude compared to polyester. Furthermore, strand count significantly impacted haptic feedback, with two-end elastane braiding delivering a 47% higher frequency and 150% greater amplitude than single-end elastane braiding. These findings indicate that vibrotactile feedback is influenced not only by the external compression forces and materials introduced during fabrication but also by the specific properties and configuration of the braiding material. This understanding is useful for achieving accurate and consistent haptic feedback in e-textiles with ERM-based electronics.

The observed changes in dynamic response are particularly important when considering real-world use, where body movements such as flexion and extension impose varying forces on the haptic yarn depending on the placement of the garment. Additionally, the amplitude was found to be sufficient to be easily identified (0.1-0.5 ms^−2^). Thus, these results require further clarification to identify the underlying reasons for the observed changes in vibration behaviour of the actuator.

To understand this, the following characteristic equation (1), of a DC motor which explains the electromechanical characteristic of the ERM motor, can be used. Here, the frequency equation is expressed as follows: *V*_*m*_ is the supply voltage, *R*_*a*_ is the armature circuit resistance, *I*_*a*_ is the armature current, *k*_*b*_ is the back EMF equation constant, and Φ is the magnetic flux.

1 $$f=\frac{{V}_{m}-{R}_{a}{I}_{a}}{60{k}_{b}\Phi }$$

During the testing procedures, the supply voltage (*V*_*m*_) remained at 3.2 V and armature current (*I*_*a*_) was measured, and no changes were observed with loading, as provided in the Supplementary Fig. 2. Given that the *R*_*a*_ was 30 ± 20%*Ω* and *k*_*b*_ remained constant, the only variable was the magnetic flux (*Φ*). The observed frequency shifts are therefore hypothesized to result from deformation of the motor’s internal components under load, which can alter *Φ* and consequently affect the motor speed. However, other motor non-idealities (i.e., temperature-dependent resistance variation, load-dependent friction, and changes in effective back-EMF under operational loading) were not independently characterized and cannot be fully excluded as contributing factors on the basis of the present data alone. Under this hypothesis, continuous loading by the test rig and the additional radial forces introduced by the fibre coverings (particularly in braided configurations) impose further compression on the motor body, eventually increasing internal deformation and producing the observed rise in frequency after braiding. Accordingly, the higher frequencies measured in the two-end elastane and two-end polyester braids, relative to the single-end elastane braid, can also be attributed to the increased radial forces arising from higher strand counts and the associated increase in textile coverage.

### Integration of Haptic Electronic Yarns into Knitted Textile Structures

**Fig. 5:**
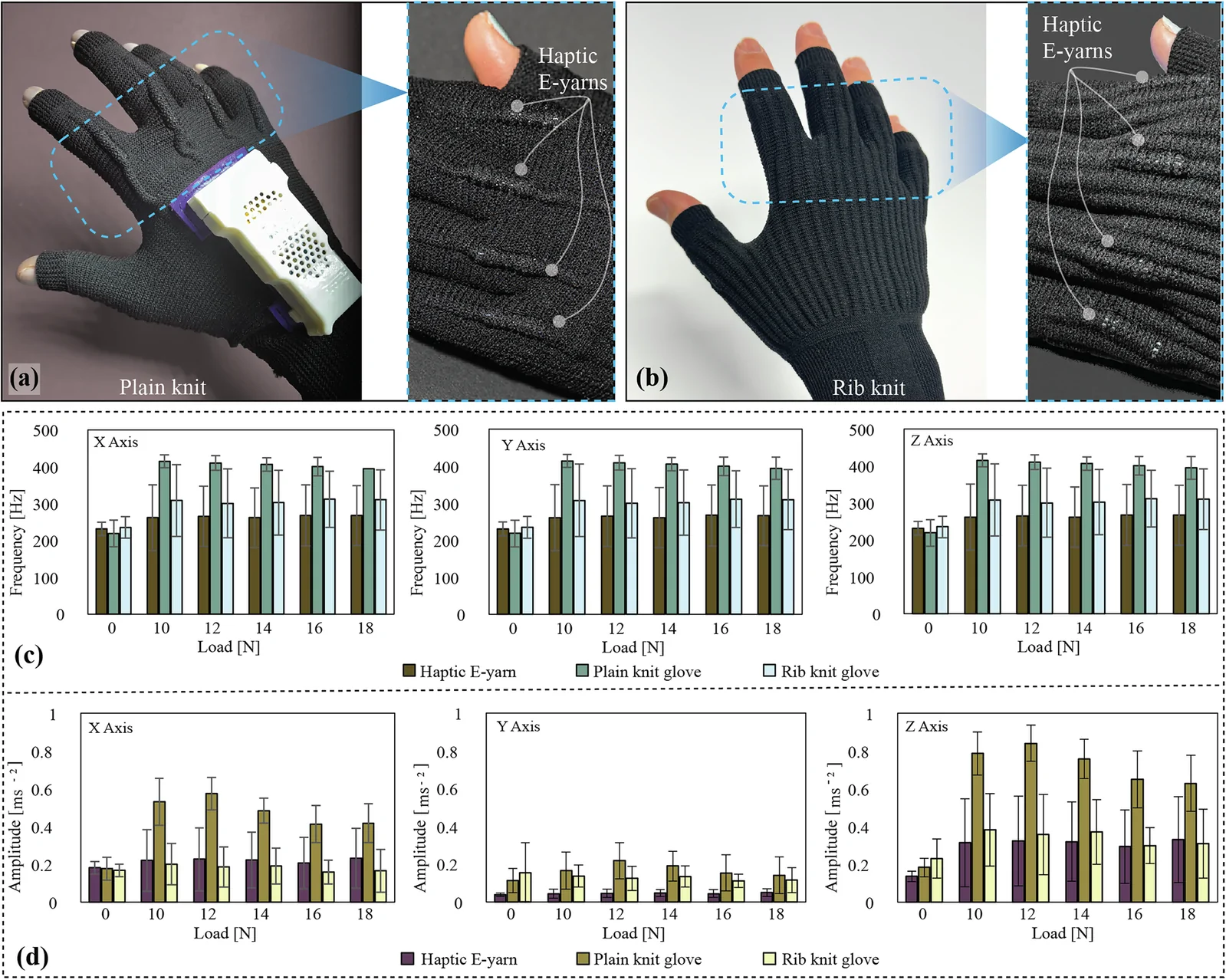
Overview of the final integration of the engineered haptic electronic yarn into seamless knitted glove systems. **a** Integration of four haptic electronic yarns along the proximal phalanges of the fingers using 3D-knitted channels in a plain knit structure for optimal placement and responsiveness. **b** A rib knit design for embedding the haptic yarns, offering improved aesthetics and consistent structural alignment across the glove. **c** Frequency response of the haptic electronic yarn in the X, Y, and Z axes under progressive loading, comparing unsheathed soldered component with post-integration into plain and rib knit textile structures. **d** Corresponding amplitude response in all three axes under progressive loading across the same configurations, highlighting the influence of textile integration on vibration performance.

Development of electronic textiles with haptic feedback incorporate embedded haptic E-yarns in knitted structures. This was achieved by inserting engineered haptic E-yarns into knitted hollow channels using a flat bed knitting machine (Stoll ADF32W, 7.2 gauge; Karl Mayer Stoll GmbH, Oberhausen, Germany). Seamless knitted glove prototypes as haptic interfaces were developed incorporating these channels oriented along the finger directions (Fig. 5a, b).

A prototype with a *plain knit* double jersey structure was produced by embedding haptic E-yarns into the knitted channels, resulting in the glove shown in Fig. 5a. However, this design exhibited reduced aesthetic appeal due to the conspicuous channels and visible haptic components, as well as structural non-uniformity. To address these limitations and improve inconspicuousness, a *rib knit* double jersey structure was employed for the second prototype (see Supplementary Movie 2). The rib knit structure provided enhanced aesthetics and improved structural uniformity by concealing the haptic components more effectively (Fig. 5b).

Each glove incorporated four engineered haptic E-yarns positioned within these 3D-knitted channels along the proximal phalanges (Fig. 5a, b). Channel dimensions were kept consistent across both knit structures to ensure comparable fit and yarn placement. Both knitting techniques secured the haptic E-yarns within distinct textile architectures, potentially influencing vibrotactile performance. To assess this performance, each of the four haptic E-yarns integrated within each glove prototype (plain knit and rib knit) was individually tested using the vibration measurement rig; then the mean and standard deviation of these four measurements were computed per glove. It should be noted that these measurements originate from a single fabricated glove prototype per knit structure and therefore do not constitute independent glove-level replicates; accordingly, the reported statistics are descriptive and reflect variation within one single glove rather than differences between multiple gloves. The mean vibration responses and associated standard deviations across the four channels for each glove prototype (plain knit and rib knit) are presented in Fig. 5c, d.

The frequency response of integrated E-yarns showed only minor deviations compared to standalone yarns under no-load conditions (Fig. 5c). Under applied loads ranging from 0 to 18 N, the plain knit glove exhibited a substantial increase in vibration frequency, reaching 414.7 ± 17.4 Hz compared to 219.0 ± 35.9 Hz for the standalone haptic E-yarn. After loading up to 18 N, the frequency slightly decreased to 394.7 ± 30.4 Hz. Amplitude followed a similar trend, with an initial sharp rise and subsequent decline (Fig. 5d). In contrast, the rib knit glove exhibited only minor changes in frequency and amplitude under load. Overall, integration into either knit structure resulted in a 1–5% variation in frequency and an 18–40% increase in amplitude compared to standalone yarns. However, when force was applied, the plain knit structure demonstrated substantial increases in both frequency (75%) and amplitude (286%), whereas the rib knit structure remained relatively stable (Fig. 5c, d).

These differences can be attributed to the distinct surface geometries and through-thickness mechanics of the knitted structures, which together control the contact area, load distribution, and the effective compressive modulus at the actuator site. Although the same external force was applied to all samples, the rib knit dispersed the load across multiple ribs, increasing the effective contact area and reducing local stress transmitted to the embedded actuator. In contrast, the plain knit concentrated force onto fewer contact points (specifically a single knitted channel aligned with the load and containing the haptic E-yarn) thereby elevating the local compressive stress and amplifying the vibration response, as described by Equation (1). Together, these results demonstrate that ERM based vibrotactile sensitivity was governed by textile architecture, integration methodology and external forces collectively.

### Wash Reliability

Wearable electronic textiles require regular laundering to maintain hygiene and usability, similar to conventional textiles. However, the washing process introduces complex mechanical stresses on delicate electronic components, making washability one of the most critical and challenging performance characteristics to achieve^48,56,57^. Despite these challenges, it is essential to evaluate the durability of developed E-yarns and prototypes incorporating haptic E-yarns under repeated washing cycles to ensure reliability in practical applications.

In this study, the wash durability of engineered haptic E-yarns and a prototype haptic glove with embedded haptic E-yarns was assessed following procedures adapted from ISO 6330:2021 and IEC 63203:2023 wash standards. These standardized protocols ensured consistent and reliable evaluation of performance after laundering^58,59^.

All test specimens, including ten standalone haptic E-yarns (300 mm length) and a prototype glove embedded with four haptic E-yarns were tested. Specimens were inspected and electrically tested to confirm initial functionality prior to testing. X-ray radiography imaging was also performed before initiating the wash test to document the state of internal conductive elements. Two-wire resistance measurements were performed at the ends of the two Litz conductors in the haptic E-yarn, across a 150 mm conductive length, using a digital multimeter (34461A, Keysight Technologies, California, USA). The vibration characteristic was recorded using the custom test rig described earlier.

A total air-dry load mass of 2.05 kg was achieved using additional polyester ballast pieces. Each wash cycle consisted of a main wash at 40 °C for 15 min, followed by four rinse cycles (10 min in total) and a final spin cycle (5 min). All specimens underwent 20 consecutive wash and dry cycles. Specimens were line dried after each wash cycle and electrically tested to confirm functionality. Resistance and vibration measurements were repeated after the 10^th^ and 20^th^ wash cycles, and X-ray radiography imaging was conducted after the 20^th^ cycle. A detailed description of the wash procedure is provided in the Supplementary Wash Report.

**Fig. 6:**
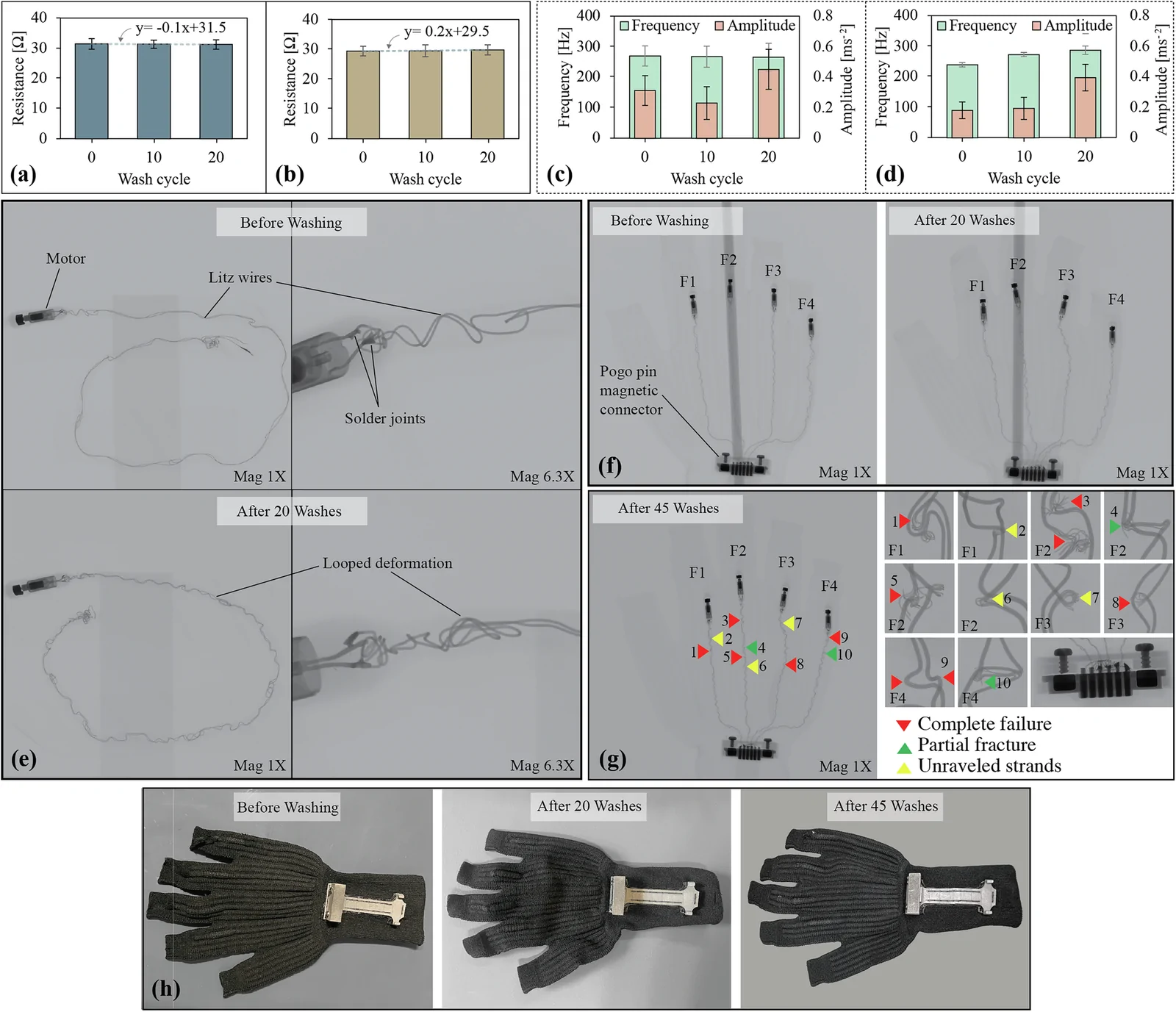
Assessment of washability of the developed haptic E-yarns and electronic textile devices which incorporate haptic E-yarns. **a** Influence to the resistance of conductive components in haptic E-yarns under washing. **b** Influence to the resistance of conductive components in haptic electronic glove under washing. **c** Effect of vibrotactile feedback of the haptic E-yarns under wash cycles. **d** Effect of vibrotactile feedback of the haptic electronic glove under wash cycles. **e** X-ray radiography images of the haptic E-yarn sample no. SWa5 before and after 20 wash cycles. **f** X-ray radiography images of the haptic electronic glove before and after 20 wash cycles. **g** X-ray radiography of the glove after 45 wash cycles, showing failure mechanisms of integrated haptic E-yarns (F1-F3: finger indices; 1-10: failure points). **h** Photographs of the haptic electronic glove before washing, after 20 wash cycles, and after 45 wash cycles.

No functional failures or visible damages were noticed even after the 20^th^ wash cycle of any of the ten standalone haptic E-yarns and four haptic E-yarns embedded in the prototype glove. This showed high survival rates (100%) of samples throughout rigorous and repeated washing and drying. However, noticeable shrinkage and deformation of the textile structure in the washed haptic E-yarns were observed after the 20^th^ wash cycle (see Supplementary Fig. 3). The mean electrical resistance values for the standalone haptic E-yarns and the integrated haptic E-yarns in the glove are presented in Fig. 6a, b accordingly. For embedded haptic yarns, a slight upward trend in resistance was observed, approximately a 0.4% increase after the 10^th^ wash cycle and a 1.0% increase after the 20^th^ cycle. In contrast, standalone haptic yarns exhibited relatively stable resistance values, showing a slight decrease of 0.4% after 10 cycles and 0.3% after 20 cycles. In general, minimal resistance drift was observed across repeated wash cycles. Figure 6c, d present the vibration frequency and resultant amplitude of the standalone and embedded haptic E-yarns after each set of 10 wash cycles. The standalone haptic yarns experienced a slight reduction in vibration frequency of 0.9% after the first 10 wash cycles (10^th^ cycle) and 0.7% after the second 10 wash cycles (20^th^ cycle). However, the vibration amplitude showed notable changes: a 26.2% decrease after the 10^th^ wash cycle, followed by a significant 96.0% increase after the 20^th^ wash.

For embedded haptic yarns inside the glove, notable increases in vibration frequency were recorded: 12% after 10 washes and a slight increase of 5.2% after 20 washes. In contrast, the vibration amplitude exhibited more prominent changes, increasing by 6.9% after the 10^th^ wash and by 105.9% after the 20^th^ wash. These findings clearly demonstrate the significant influence of repeated laundering on vibrotactile feedback in E-textiles incorporating haptic E-yarns. This influence further reveals the same trend as described by Equation (1): shrinkage of the rib knit after drying introduced additional compression on the actuator, further increasing its vibration output. Although haptic functionality was retained throughout the wash cycles, a noticeable calibration drift was observed. This finding has important practical implications for the long-term deployment of haptic e-textiles: systems intended for repeated domestic laundering will likely require either a post-wash recalibration procedure or a preconditioning protocol to ensure consistent vibrotactile feedback prior to use.

Pre- and post-wash X-ray radiography images of all specimens provided direct evidence of the impact of wash cycles on internal conductive structures. Particular attention was given to the micropod-wire interface, as this region was identified in previous experiments and studies as vulnerable due to its high stiffness gradient^48,49^. No cases of fracture, discontinuity, or detachment were observed in the conductive elements or soldered joints. However, increased looped deformations and partial untwisting of the multi-strand in Litz wires were evident after washing (see Fig. 6e). These mechanical alterations did not translate to catastrophic electrical failure or loss of function but may contribute to the future failures. X-ray radiography images of the glove-embedded haptic E-yarns also revealed looped deformations in the Litz wires (see Fig. 6f). However, no broken copper strands in Litz wires were detected in any of these samples. A full comparison of the X-ray radiography images of pre- and post-wash is provided in the Supplementary Wash Report. To determine the maximum wash durability and identify potential failure points, the wash test was further extended until all haptic E-yarns integrated into the glove had failed. The E-yarns along the middle finger (F2) and ring finger (F3) failed after the 23rd wash cycle, whereas the E-yarn on the index finger (F1) remained functional up to the 40th cycle. The E-yarn on the little finger (F4) failed after 45 wash cycles. Figure 6g presents the X-ray radiography images showing the failure locations after all 45 wash cycles.

One failure of Litz wires was located near the micropod-wire interface, a region previous studies identified as a high-risk zone due to high stiffness gradients in the conductive pathway (failure location no. 9 in Fig. 6g)^48,49^. All remaining failures occurred along the Litz wires between the micropod and the pogo-pin magnetic connector (see Fig. 6g). Notably, each failure occurred at points where the Litz wires were bent, indicating that failure arose from bending fatigue of the copper strands rather than tensile loading. The earlier failure of F2 and F3 is likely due to their position in the glove, where the middle and ring fingers experience greater bending, curvature, and inter-finger contact during handling and laundering. Therefore, these locations may be subjected to increased cyclic strain and connector movement, accelerating mechanical fatigue relative to the outer finger channels. Figure 6h shows the glove after repeated washing, revealing minor structural changes apart from slight shrinkage.

### Haptic Perception Ability

The haptic E-yarns integrated into the knitted glove generate vibrations that can mechanically couple through the textile substrate and, potentially, propagate through the soft tissue and bones of the hand to neighbouring skin regions^60,61^. Perception of these high-frequency cues also depends on the mechanoreceptors (*Pacinian corpuscles*) that encode them, each with characteristic spatial and temporal response properties determined with their receptive fields^62,63^. Consequently, both the spatial placement of the stimulation sites and the temporal separation between stimuli are crucial for evaluating users’ ability to identify and localize haptic cues to enable reliable tactile communication^64,65^.

To evaluate this perceptual performance, multi-stage targeted human-subject experiments were conducted on the right dorsal hand, where the haptic E-yarns were positioned. This approach accounts for mechanical factors, such as vibration transmission through the glove and hand, and also perceptual processing by the human sensory system. In particular, known perceptual phenomena such as haptic illusions^13,66,67^ and spatial summation^68–70^ can lead to systematic distortions between the perceived locus of a stimulus compared to the actual. By measuring user localization performance directly while wearing the glove, this approach captures the combined influence of mechanical coupling and human sensory interpretation under realistic wearing conditions. Although the mechanical crosstalk isolation between adjacent actuator channels cannot be directly assessed under the tested conditions, this methodology directly evaluates whether the channels achieve functional perceptual separability. Thus, this explains whether users were able to reliably distinguish and localize stimuli across channels; however, to further characterize the mechanical transmission of vibration across channels (or crosstalk isolation), a future experiment could employ accelerometer-embedded yarn^43^ to quantify any residual inter-channel coupling.

Ethical approval was obtained from Nottingham Trent University (application ID 1621411, approved 07/10/2024). Informed consent was collected from all participants, and all study procedures complied with the Declaration of Helsinki and relevant institutional guidelines. Ten healthy female participants (*n* = 10; mean age 28.6 ± 5.0 years) with no self-reported neurological or dermatological conditions were recruited. The decision to recruit an exclusively female cohort was deliberate and methodologically motivated, given that tactile perception thresholds and haptic sensitivity are known to vary with demographic factors such as biological sex, age, hand morphology, and the presence of sensory impairments^71–73^. By recruiting a demographically homogeneous cohort (non-impaired females only), sources of inter-participant variability were intentionally minimized. In particular, this controlled for variations in glove fit due to hand morphology and differences in tactile sensitivity (e.g., two-point discrimination), both of which directly influence localization accuracy. This study represents an initial perceptual validation conducted on the right dorsal hand of healthy female participants; therefore, generalizability to individuals with deafblindness, to males, other age groups, and left-handed use remains to be established.

After a brief familiarization session (less than 5 min), participants learned the finger-labelling convention. During testing, the hand was positioned behind a visual barrier to remove visual cues. Our previous work introduced a methodology for evaluating haptic localization performance^46^. In that methodology, three parameters were systematically examined: spatial distribution of stimulation sites, temporal interval (TI) between pulses, and stimulus intensity defined by pulse width (PW). The present study incorporates and extends this methodology using the same experimental framework to identify optimal parameter settings. Spatial configurations on the dorsal hand were tested alongside TIs ranging from 0 to 2000 ms and PWs of 10–60 ms. The upper bounds were chosen to avoid excessive delays that reduce information-transfer rate and to prevent prolonged vibration, which eventually decreases battery life in wearable systems. Prior studies similarly explored TIs below 2000 ms^74,75^. A detailed protocol is provided in our previous work^46^.

**Fig. 7:**
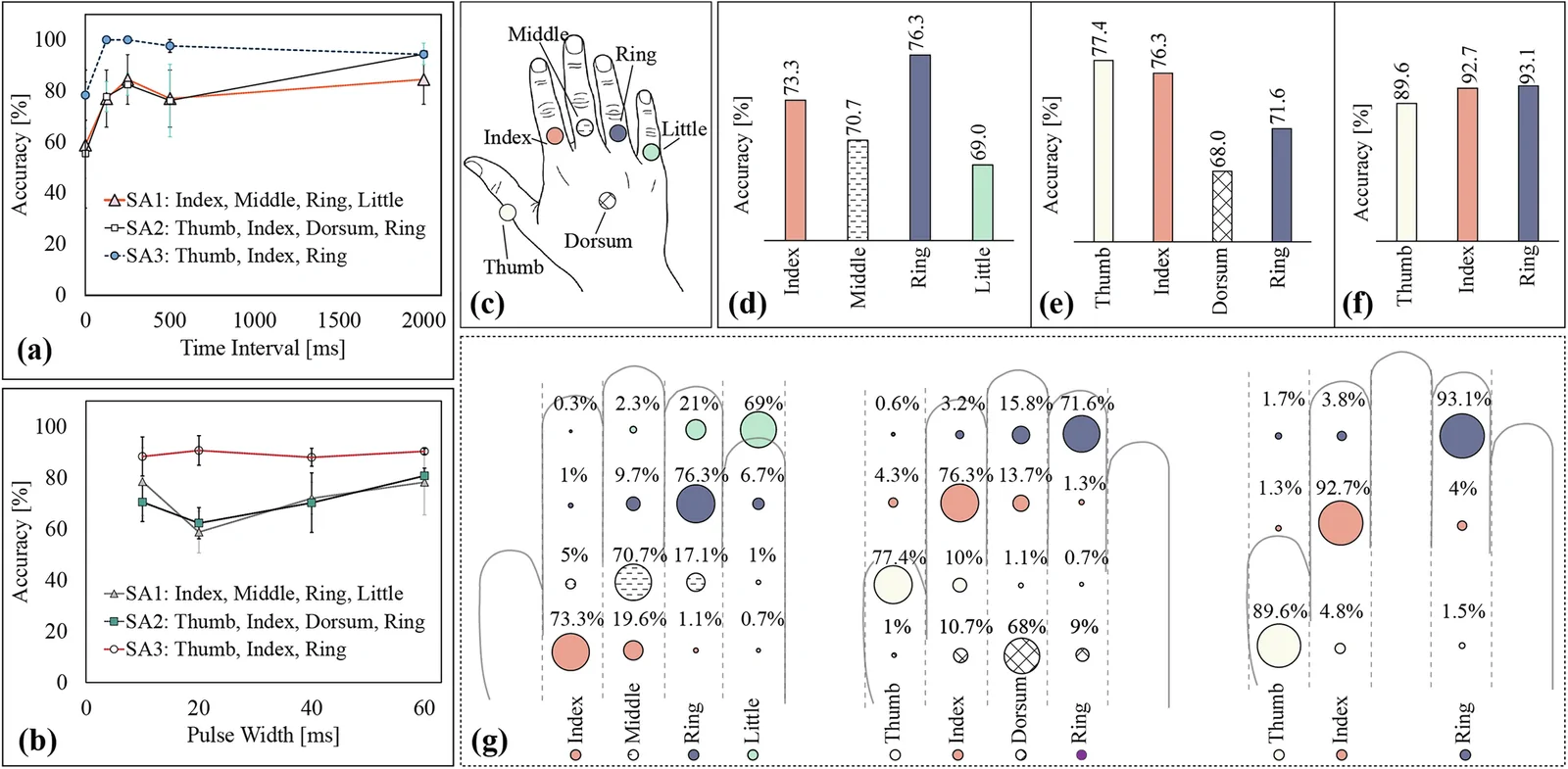
Assessment of haptic perceptibility for multi-stimulus haptic communication systems. **a** Localization accuracy across different inter-stimulus intervals (TI) across three spatial arrangements (SA). **b** Localization accuracy for varying stimulus intensities (pulse widths, PW) across three spatial arrangements (SA). **c** Spatial arrangements assessed on the dorsal side of the right hand, including the proximal phalanges of the thumb to little finger and the dorsum. **d** Average localization accuracy for the spatial arrangements using the proximal phalanges of the index, middle, ring, and little fingers. **e** Average localization accuracy for the spatial arrangements using the proximal phalanges of the thumb, index, and ring fingers plus the dorsum. **f** Average localization accuracy for the spatial arrangements using the proximal phalanges of the thumb, index, and ring fingers. **g** Distribution of mislocalization responses across finger locations for the different spatial arrangements.

The initial spatial configuration described in our previous work (proximal phalanges of the index, middle, ring and little fingers) yielded an overall localization accuracy of 58.8% at a TI of 0 ms, increasing to 88% as the TI was extended to 2000 ms, with performance plateauing at approximately 250 ms (see Fig. 7a). Variations in PW had minimal effect, aside from a decrease in accuracy at 20 ms PW (see Fig. 7b). Finger-wise localization accuracy for this layout was 73.3% (index), 70.7% (middle), 76.3% (ring), and 69.0% (little), resulting in an overall accuracy of 72.3%. Building on these findings, the spatial arrangement was further refined in the present study (see Fig. 7c).

A second spatial configuration replaced the middle and little fingers with the thumb and dorsum to increase spatial separation. Thumb performance was highest at 77.4%, followed by the index (76.3%) as shown in Fig. 7e. The ring finger showed a modest decrease (71.6%), and the dorsum yielded the lowest accuracy (68%). Confusion matrix analysis showed that replacing the middle finger with the thumb reduced misclassification between the index finger and its neighbours; however, introducing the dorsum increased confusion, demonstrating that greater spatial separation alone does not necessarily improve localization performance.

Based on these findings, a refined configuration targeting only the proximal phalanges of the thumb, index, and ring fingers was evaluated. This arrangement substantially improved performance: localization accuracy increased to 89.6% for the thumb, 92.7% for the index, and 93.1% for the ring finger, yielding an overall accuracy of 92.0%, an improvement of 17.5% compared with the previous spatial configuration (see Fig. 7f). Mislocalization rates also fell below 5% (see Fig. 7g). Residual errors were primarily due to incorrect ordering of perceived stimuli (e.g., index–thumb–ring perceived as thumb–index–ring), rather than incorrect identification of the stimulated finger. These results indicate that the optimized layout reduces cross-finger interference and improves perceptual clarity. Temporal-response analysis (see Fig. 7a) showed a characteristic accuracy plateau around 250 ms TI, consistent with previous observations, but with higher overall accuracy and lower inter-participant variability due to the refined spatial configuration. The intensity study (see Fig. 7b) revealed improved accuracy across all PWs relative to previous arrangements.

Studies on fingertip localization similarly report wide performance ranges (45–90%) depending on stimulus site and temporal spacing^75^. Other work highlights that users detect tactile stimuli more reliably than they can localize them, and that localization improves with training and well-chosen temporal parameters^76^.

Together, these results demonstrate that optimized spatial placement of the haptic E-yarns enables highly reliable perception of tactile cues, achieving localization accuracies exceeding 90% with minimal cross-finger confusion, thereby supporting effective tactile communication for everyday assistive applications. Under these operating conditions (4% duty cycle, 10 ms pulse width per stimulus, and an inter-stimulus interval of T=250 ms), the estimated battery operating time of the glove is approximately 1.6 days, as detailed in the Supplementary Table 7. This level of performance highlights the strong potential of the developed haptic glove as a practical and energy-efficient platform for everyday tactile communication.

## Discussion

This study demonstrates the design, development, optimization, and integration of a novel haptic electronic yarn for wearable applications. The secure encapsulation of electronic components within textile yarns was critical for achieving reliable and perceptible haptic functionality. Iterative improvements to the micropod enclosure design enabled robust electrical connections to the actuators and effective fibre coverage of the E-yarns. Introducing conical geometries in the micropods significantly enhanced the protection of solder joints and facilitated uniform textile braiding. This optimized encapsulation reduced the force acting on soldered terminal joints by up to 99.99% at a maximum bending angle of 90°, preventing electrical failures observed in earlier prototypes and improving durability under mechanical stresses such as bending and tension. Furthermore, the textile covering provided a tactile feel and aesthetic appeal, allowing the haptic E-yarn to be integrated inconspicuously within knitted substrates. These advancements collectively establish a pathway for developing robust, functional, and visually acceptable haptic E-textiles for next-generation wearable systems.

Given that the vibration characteristics of ERM cylindrical motors vary under compression forces, material selection for E-yarn fabrication was a critical design consideration. The systematic evaluations identified the significant relationship of this compression forces with textile integrating methodologies such as braiding and knitting techniques. These findings emphasize the importance of carefully considering multiple parameters when designing haptic textiles, particularly in managing radial forces (compression forces) acting on ERM motors and optimizing compression resistance. Precise control of these factors is crucial for ensuring consistent vibrotactile feedback in terms of both frequency and amplitude. This knowledge not only holds value in developing haptic devices with E-yarn technology but would be useful when developing any vibrotactile electronic textile device where the electronics are embedded within the textile structure.

This study demonstrates the potential for the real-world application of embedded haptic E-yarns in textile devices through the development of a haptic electronic glove designed as a potential assistive technology for individuals with vision and hearing impairments. User trials with a defined user group showed high accuracy in haptic localization and stimulus identification, confirming the effectiveness of skin-based communication for people with sensory impairments. However, expanding participant recruitment to include wider group (specially including elderly individuals, and people with varying degrees of sensory impairment) is essential for broader claims of generalizability.

The ability to encode such haptic cues with specific real-time information opens opportunities for continuous haptic communication, navigation assistance, and safety-related alert mechanisms, ultimately supporting improved accessibility, greater independence, and enhanced quality of life. A detailed description of the application is provided in our previous work^46^. Although the present study demonstrates a single-motor haptic E-yarn within a glove, we have also developed multi-motor and branched haptic E-yarn architectures that are suitable for wider textile integration^77^. These configurations can be incorporated into large garment regions using knitted channels, which allow the yarns to be routed, protected, and mechanically supported. Our previous work has shown that tubular knitted channels can successfully embed E-yarns across extensive areas of clothing, such as T-shirts, modular textile panels, and athletic wear^78–80^. These examples confirm that the technology is not limited to small-scale applications. Additionally, a recently accepted poster demonstrates a large-area haptic pillow sleeve, further illustrating that haptic E-yarns can be deployed across broad textile surfaces for practical assistive purposes^77^.

Rigorous wash testing of the glove, along with standalone haptic E-yarns in accordance with international wash standards, revealed high wash durability and confirmed the practical viability of the developed technology for wearable applications. Although this represents the accepted gold standard for reliability assessment in this field, it does not fully replicate the complex chemical environment arising from prolonged skin contact in everyday wear. In particular, repeated exposure to human perspiration introduces ionic species, which may accelerate the degradation of insulating and encapsulating polymer materials over extended use^81^. Characterizing these effects rigorously would require a coupled ageing protocol involving accelerated ageing test, interleaved with standardized wash cycles. Such a study represents a substantial and independent experimental programme and is identified here as a priority direction for future work.

Beyond chemical durability, the wash tests also provided strong validation of the improved encapsulation design achieved through iterative optimization, demonstrating high reliability of the electronic components under the combined mechanical stresses of repeated laundering, including bending, compression, and tensile loading. The integration of non-textile materials, such as the electronics and insulated conductors within the haptic E-yarn, has the potential to affect fabric breathability and, consequently, wearer comfort. Thus, the evaluation of air permeability of knitted structures with embedded haptic E-yarns demonstrated that the incorporation of these electronic components has a minimal adverse effect on breathability relative to standard knitted garments; indeed, the integrated structures exhibited a measurably improved airflow compared to conventional equivalents (see Supplementary Fig. 26). This is attributable to the localized stretching of surrounding knitted stitches induced by the embedded components, which increases the openness of the fabric architecture (see Supplementary Fig. 25).

The introduction of materials stiffer than conventional textile fibres in haptic E-yarn also influences fabric mechanical characteristics, particularly flexibility and drapeability, which are another additional determinant of wearer comfort. Established drape testing standards are not well-suited to the evaluation of e-textiles due to the heterogeneous and anisotropic nature of embedded electronic structures^42^. Bending stiffness was therefore assessed as a more appropriate measure, conducted in accordance with ASTM D1388 using the Shirley cantilever method (Taber Fabric Stiffness Tester) (see Supplementary Fig. 27). While textiles with embedded haptic E-yarns are inherently less flexible than conventional fabrics, the results show that they retain a reasonable degree of conformability that is not likely to compromise wearer comfort under normal conditions (see Supplementary Fig. 28).

As part of a separate study, the haptic glove was also evaluated in three focus groups: people with hearing impairments, people with visual impairments, and caregivers and professionals supporting people with dual sensory impairments. With respect to breathability and thermal comfort, one deaf participant raised the question “*Would you sweat with wearing those gloves in the summer?”*, to which another deaf participant responded *"Probably.”* No participant reported active discomfort during the session itself. With respect to fit and comfort, a blind participant noted *"It’s a bit small, I’m trying to shrink myself in the water to make it fit!”*, while another visually impaired participant remarked *"Yeah, it’s definitely more comfortable to wear.”* When directly asked about comfort, a blind participant responded: *"I think the glove would be the most comfortable option for me. I could simply put it on.”* The variability in these responses reflects the influence of individual hand morphology, glove fit, and the limited wearing time available during group sessions, and underscores the need for a more systematic comfort evaluation employing multiple glove sizes, validated comfort assessment instruments, and extended wearing sessions. This is identified as a priority direction for future work. Taken together, these findings establish the mechanical and functional robustness of the haptic e-textile design with engineered haptic E-yarns, supporting its feasibility for integration into everyday textile products and laying the groundwork for future wearable haptic applications.

## Methods

### Design and Implementation of the Haptic Electronic Yarn

The haptic E-yarn was designed to achieve key goals: delivering adequately perceivable haptic feedback by reducing damping, ensuring feasible integration for manufacturability, protecting the electronics from water and chemicals, and ensuring durability and reliability.

The design and development of the haptic E-yarn began with establishing secure electrical connections for the selected miniature vibrotactile actuator (3.2 mm diameter, 12.7 mm length, and 1.0 ± 0.2 g; NFP-3C-03208B, NFP Motors™, Hong Kong, China) to the conductive wires, followed by encapsulation of the actuator. Especially, the default PVC stiff terminal wires^82^ of the actuator (red and blue, as shown in Fig. 1b) transferred the vibration throughout the yarn, reducing the point stimulus. Replacing that default PVC terminal wires with soldering two Litz wires (NELA7/44SPSN, Type 1 Litz, OSCO Limited, Milton Keynes, United Kingdom) provided a better point stimulus. Litz wires (NELA7/44SPSN, Type 1 Litz, OSCO Limited, Milton Keynes, United Kingdom) are composed of multiple fine copper strands, each individually insulated with a polyurethane coating and then collectively covered with a protective topcoat reinforced by nylon thread^83,84^. These Litz wires had been used in a variety of other E-yarn innovations^43,85,86^ and provided a textile with a normal look and properties at the location of the wire^42^. To ensure reliable protection for the ERM motor, including its moving components and electrical connections, custom 3D-printed enclosures were employed to securely accommodate the motor (Fig. 2d iii). Stereolithography (SLA) printing was selected to achieve fine wall thicknesses (approximately ~ 0.3 mm). The enclosures were fabricated using a clear resin (Clear Resin V5, Formlabs, Massachusetts, USA) with a Formlabs SLA printer (Form 4B, Formlabs, Massachusetts, USA). Following fabrication, the actuator was positioned within the enclosure and sealed using a UV-curable resin (9001-E-V3.5, Multi-Cure® Adhesive & Coating, Dymax Corporation, Torrington, Connecticut, USA), ensuring mechanical stability and environmental protection.

To mitigate mechanical stress on the Litz wires and their soldered joints during textile processing and end-use, two high-tensile-strength support yarns (HT110T20; Vectran™, Kuraray, Tokyo, Japan) were integrated alongside the micropod (Fig. 2d iv). These support yarns also facilitated feed-in process for the subsequent braiding process (Fig. 1a).

The textile braiding served a dual purpose: it further enhanced the mechanical strength of the final E-yarn and contributed to its tactile characteristics and visual appeal, providing a textile-like appearance. This braid was constructed using a textile round braiding machine (Herzog RU 1/24-80; Oldenburg, Germany). Two yarn types and braiding parameters were investigated. Polyester (PE1716, J. H. Ashworth & Son Ltd, Kingston Mills, Hyde, Cheshire, United Kingdom; 1 ply/167 Dtex/48 filaments) and double-covered elastane yarns (20/DCY/003 Black, Stretchline, Sri Lanka; 44 Dtex Creora® elastane core, 44 Dtex/34 filament Nylon 66, 2 ply / 78 Dtex/24 filament Nylon 6) were specifically chosen for their comparable stretch behaviour (low-strain polyester and high-strain elastane) to examine how braiding material influences vibrotactile feedback.

The first configuration used covering yarn carriers, each loaded with two ends of polyester yarn (doubled). The second configuration employed either a single end or two ends of elastane yarn. These variations in strand count, single-end or two-end, were selected to investigate the effect of textile covering on vibrotactile feedback. All braiding was performed using 24 covering yarn carriers, with a lay length of 4 mm over the enclosure to ensure uniform comparison.

### Vibrotactile Measurement

Analyzing the impact of each sub-step in the E-yarn fabrication process, such as soldering, encapsulation and braiding, on the vibrotactile feedback was crucial for optimizing the design of the haptic E-yarn. Additionally, since the vibration characteristics of ERM cylindrical motors vary under compression forces, it was important to understand how vibration feedback changes under different loading conditions. This is particularly relevant because electronic textiles experience compression during human body movements. Therefore, characterizing this variability was crucial for comprehending the dynamic behaviour of the E-yarn during real-world use.

For this analysis, a custom-designed test rig was developed to measure the vibration characteristics of the haptic E-yarn under controlled loading conditions. After evaluating various conceptual designs, an effective test setup shown in Fig. 4a, was selected, as it aligned well with testing methods commonly used by actuator manufacturers. The compression force was precisely controlled by tightening a wing screw (M6, 1 mm), with continuous monitoring of the compression force measured using a force sensitive resistor (Standard Model A201; FlexiForce™, ©Tekscan Inc, Massachusetts, USA) to ensure uniformity throughout the test. The schematic of engineering drawing of the test rig is provided in the Supplementary Fig. 4.

Vibrotactile feedback was recorded using a calibrated tri-axial accelerometer (Model 4520-001; Brüel & Kjær, Nærum, Denmark) connected to a signal analyzer (Photon+; Brüel & Kjær) and controlled via RT Pro Photon software (v7.30.10). Manufacturer calibration was applied to convert voltage signals (mV) into acceleration values (ms^−2^). Root mean square (RMS) acceleration values were documented for induced vibrations. Data acquisition and processing employed a multi-frame hanning window Fast Fourier Transform (FFT) within the RT Pro Photon software.

This setup enabled precise measurement of vibration frequency and amplitude, focusing on how different fabrication stages and design variations of the E-yarn respond to compression forces.

### Pre- and Post-Testing X-ray Assessment of Haptic E-yarns

X-ray radiography (XT H 225 MicroCT, Nikon Metrology NV, Tokyo, Japan) was performed on all haptic E-yarn samples before and after cyclic bending and wash testing to assess solder-joint integrity and identify potential failure mechanisms. Particular focus was placed on mechanically sensitive regions, especially the micropod-wire interface where a pronounced stiffness gradient exists (an area previously identified as a common failure location in e-textile interconnects^48,49^). This protocol enabled direct structural comparison of each sample in its pre- and post-fatigue state. Each specimen was imaged at high magnification with emphasis on these critical interfaces, including solder joints and regions exhibiting steep stiffness gradients.

## Supplementary information

Supplementary information

Supplementary Movie 1

Supplementary Movie 2

## Data Availability

The data supporting the findings of this study are available in the main text and the Supplementary Information. The raw data used to generate the figures in this article are available at https://doi.org/10.6084/m9.figshare.30961862.
