# Supplementary material for: Novel Haptic Electronic Yarn for Wearable Textiles: Vibrotactile Feedback via Knitted Channels for Skin-Based Communication
Source: Npj Flex Electron. 2026 Jul 4;10(1):110. doi: 10.1038/s41528-026-00613-w (PMC13713563; doi:10.1038/s41528-026-00613-w)
Supplement: Supplementary file 1 [file 41528_2026_613_MOESM1_ESM.pdf]

Supplementary Information

Novel Haptic Electronic Yarn for Wearable Textiles: Vibrotactile Feedback via Knitted Channels for Skin-Based Communication

M. Ehelagasthenna [1, *], A. M. Shahidi[1], P. Lugoda[2,3, *], P. Ebrahimi[1], C. Oliveira[1], D. Eberl-Craske[3], W. Hurley[1], T. Dias[1], and T. Hughes-Riley[1, *]

[1]Nottingham Trent University, Nottingham School of Art & Design, Nottingham, NG1 4GG, United Kingdom
[2]University College London, Department of Orthopaedics and Musculoskeletal Science, Division of Surgery & Interventional Science, Brockley Hill, Stanmore HA7 4AP, United Kingdom
[3]Nottingham Trent University, School of Science & Technology, Nottingham, NG11 8NS, United Kingdom
[*]malindu.ehala@gmail.com, p.lugod@ucl.ac.uk, theo.hughes-riley@ntu.ac.uk

The authors acknowledge the support of Nottingham Trent University (United Kingdom), which provided a studentship to M. Ehelagasthenna for this research. Additional financial support and research facilities were provided by the Nottingham School of Art & Design. The authors also thank the Imaging Suite at the Medical Technologies Innovation Facility (MTIF) and Graham Hickman for access to facilities that assisted this work. Mark Flanagan is thanked for manufacturing the custom test rig used for vibration measurements, and Stephanie Smith and Laura Mathews are acknowledged for their support with 3D printing. The authors further acknowledge the contribution of Tharushi Peiris in preparing wash-test ballast and thank Professor Richard Bibb for his critical review of the manuscript. This research was funded by the Engineering and Physical Sciences Research Council (EPSRC) grant EP/T001313/1: *Production engineering research for the manufacture of novel electronically functional yarns for multifunctional smart textiles*

Mesh Optimization Details for the Finite Element Analysis

Mesh details			Value
Element	Statistics	Nodes	80,230
		Elements	258,072
	Defaults	Element size	0.5mm
		Element order	Linear
Sizing	Face sizing	Growth rate	1.1
		Litz wire	0.3mm
		Fixed Boundary	0.03mm
Quality	Element quality	Average	0.6
		At solder joint	0.677-0.975
	Aspect ratio	Average	3.6
		At solder joint	1.39-3.6
Inflation		Transition ratio	0.275
		Maximum layers	5
		Growth rate	1.2

Supplementary Table 1. Mesh parameters used in the finite element analysis for optimizing the enclosure design.

Application of Haptic E-yarn Design Methodology on Multiple ERM Motor Models

**Manufacturer**	NFP motors	NFP motors	NFP motors	NFP motors
**Model No.**	NFP-P4611	NFP-E408S	NFP-3C-03208A	NFP-609-5.14-0675
**Dimensions**	ϕ=3 mm, L=6.7 mm	ϕ=4 mm, L=8 mm	ϕ=3.8 mm, L=8.3 mm	ϕ=10 mm, L=11.5 mm
**Voltage**	2.7 V DC	3 V DC	3 V DC	3 V DC
**RPM**	14,000	11,000	17,500	6,500
**Electrical connection**	SMT/ Soldering	SMT/ Soldering	SMT/ Soldering	Soldering
**Terminal Type**	Pad	Pad	Pad	Wire-lead
**Inter-terminal distances [mm]**	4	3.5	4	6.1
**Weight [g]**	0.6	2	0.8	1.2

Supplementary Table 2. Incorporated different ERM motors to apply haptic E-yarn design configuration.

Model No.	NFP-P4611	NFP-E408S	NFP-3C-03208A	NFP-609-5.14-0675
Default Vibration Motor	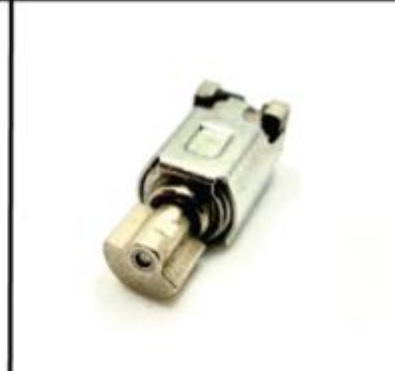	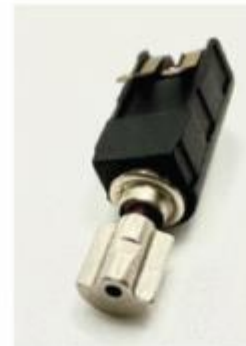	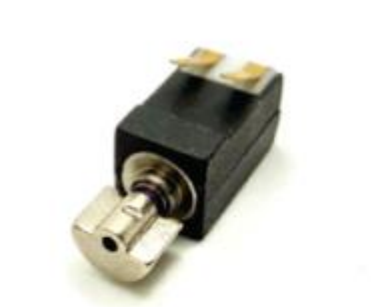	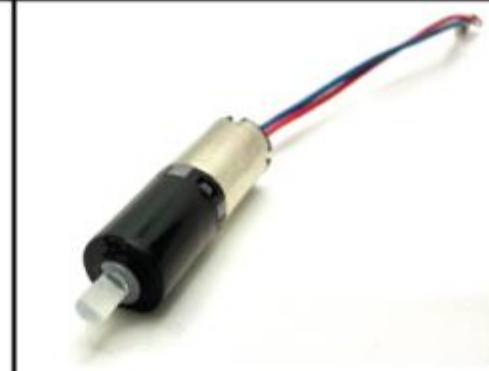
**Step 01:** Preparing motor terminals for soldering	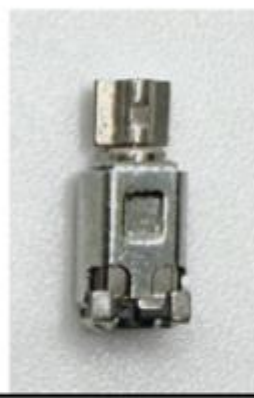	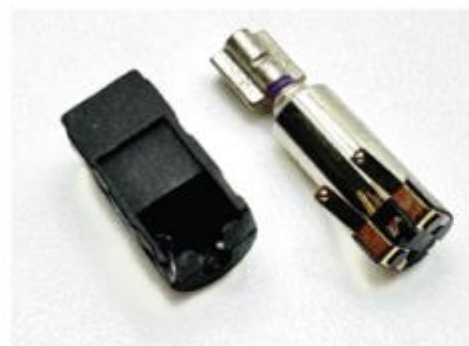	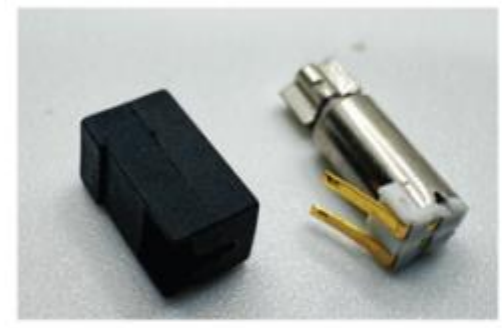	
**Step 02:** Solder Litz wires onto motor terminals	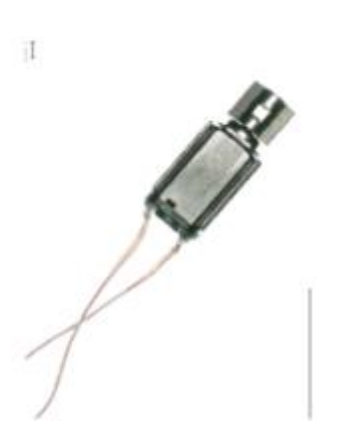	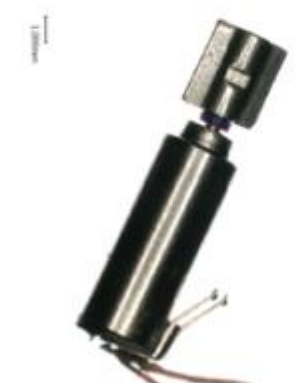	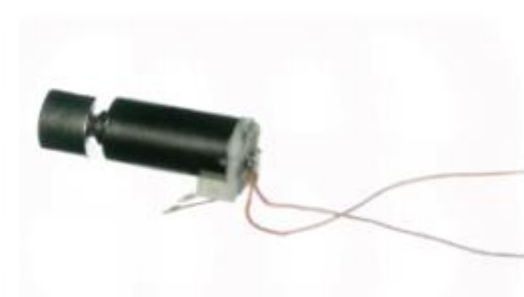	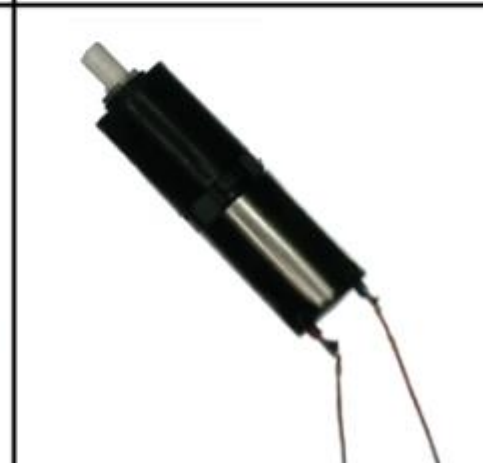
**Step 03:** Preencapsulate solder joints	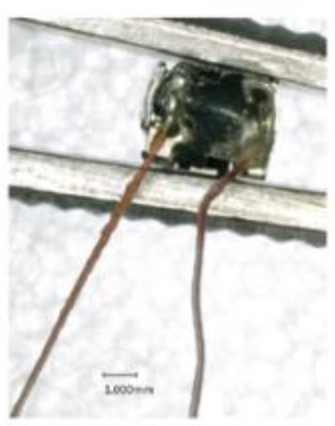	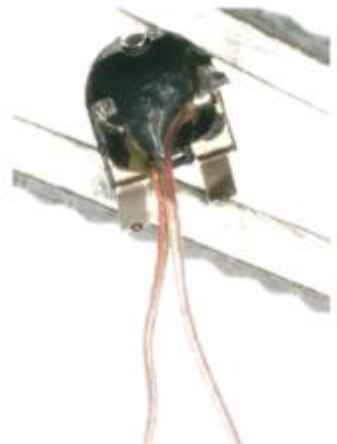	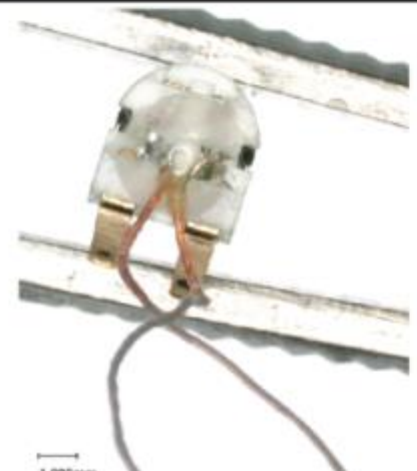	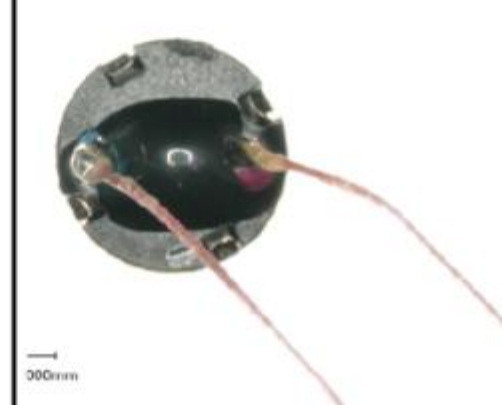
**Step 04:** Insert soldered components into 3D printed enclosures and secure them using resin. Then attach Vectran yarns	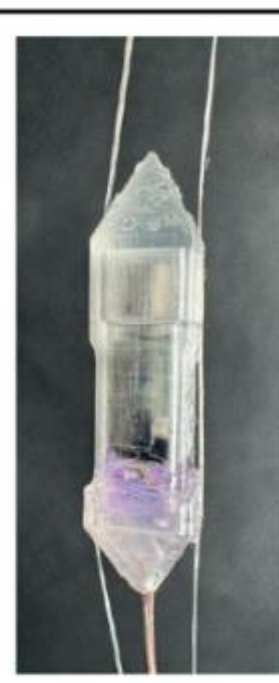	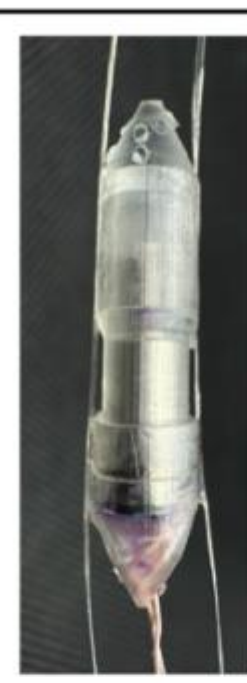	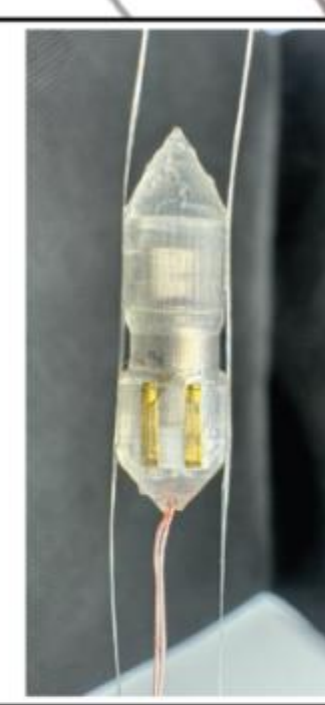	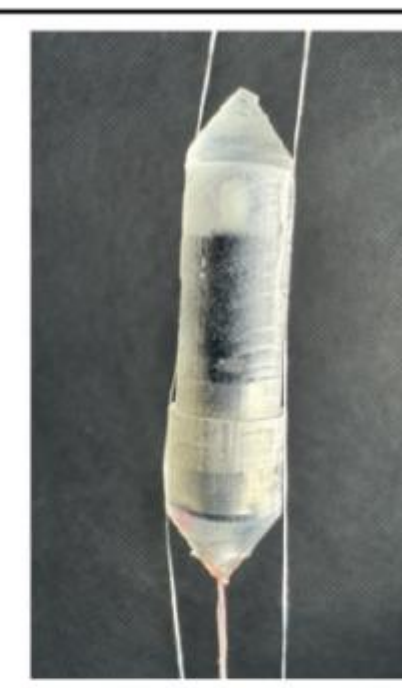
**Step 05:** Braide the encapsulated components to achieve the final haptic E-yarn structure	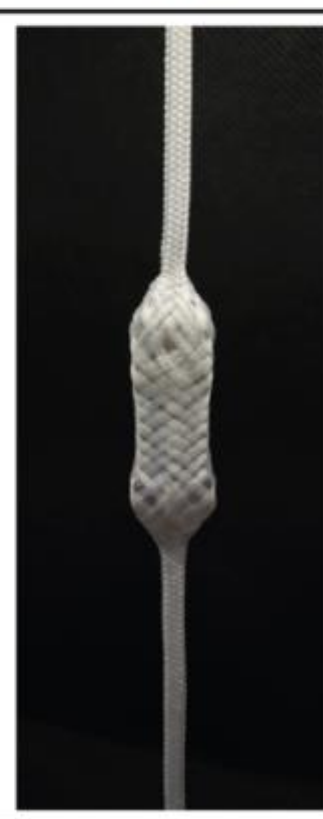	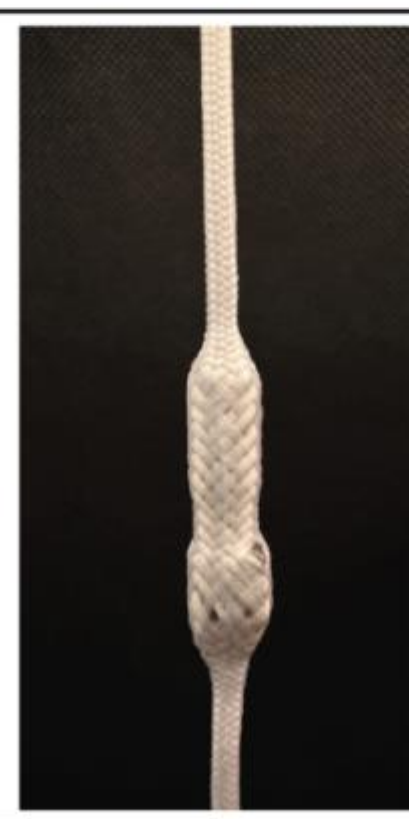	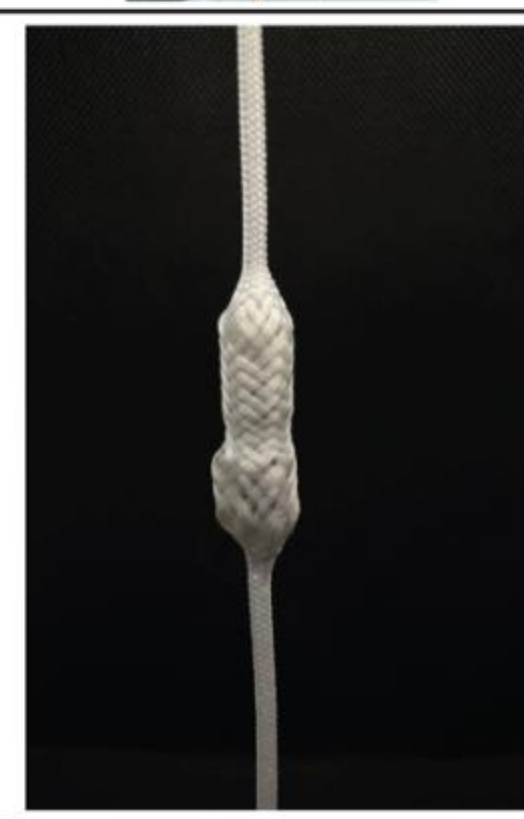	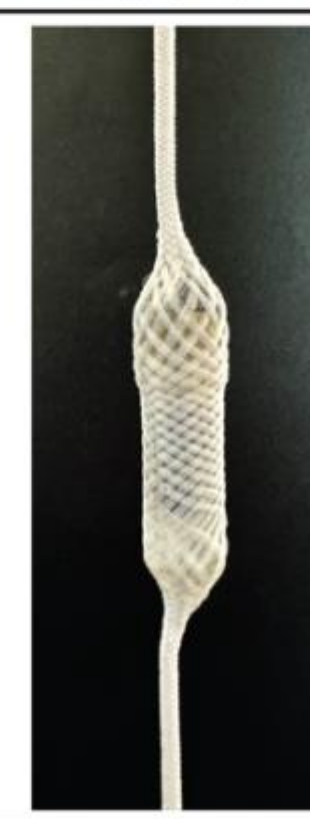

Supplementary Table 3. Haptic E-yarn fabrication steps for four different ERM models as shown in Supplementary Table S2.

Design and Development of a Custom Test Rig for Cyclic Bending Test

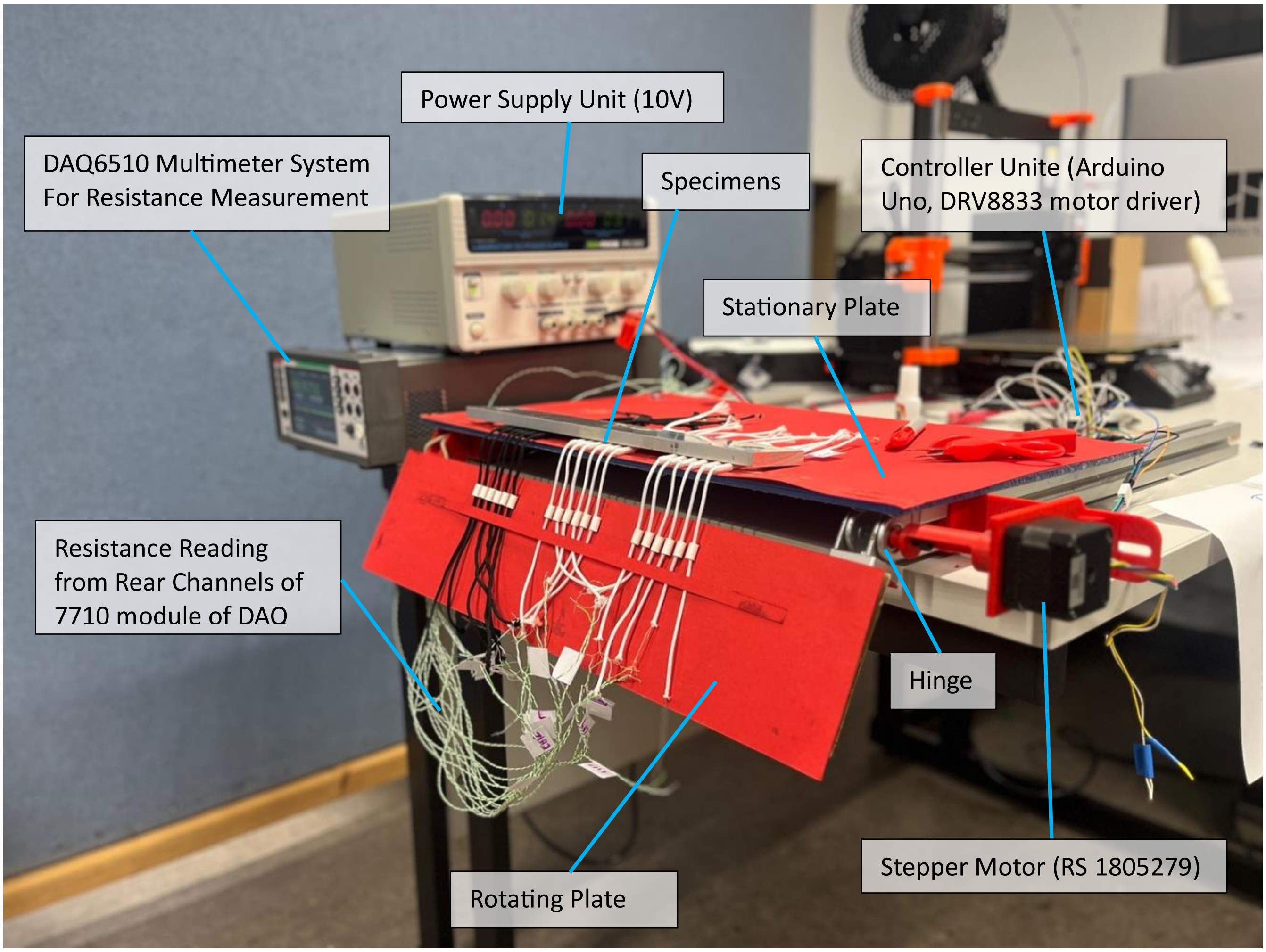

Supplementary Figure 1. Experimental setup developed for cyclic bending of haptic E-yarns.

Current and Power consumption of the EMR motor with respect to the Continuous Loading

As discussed in the article, during the testing procedure for vibration analysis of the haptic electronic yarn using the test rig, the supply voltage (V_m) was maintained at 3.2 V, and the armature current (Ia) was measured with respect to the loading as follows.

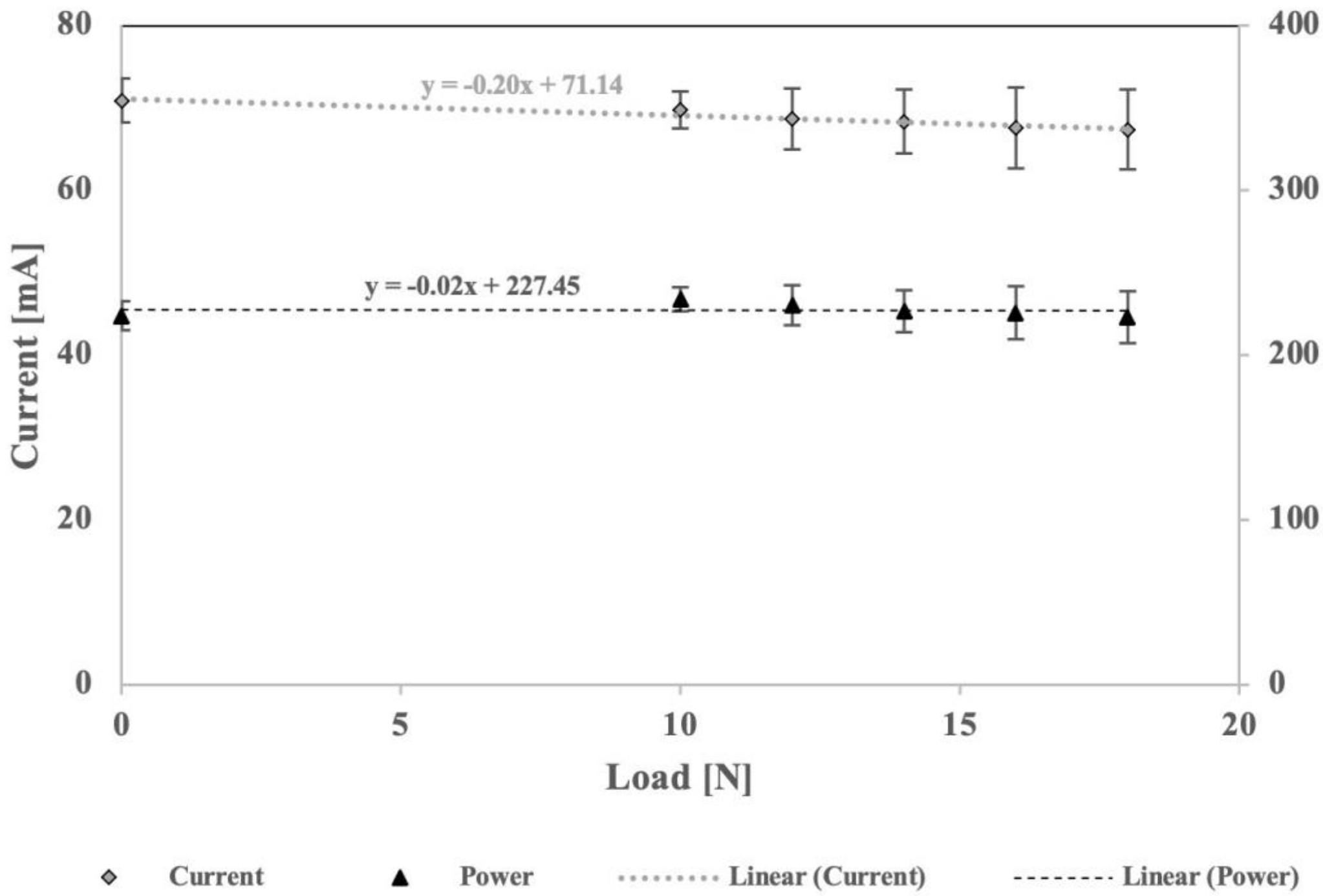

Supplementary Figure 2. Electrical characteristic of the engineered haptic electronic yarn with continuous loading

Pre- and Post-Wash Microscope Imaging of the Haptic E-yarns

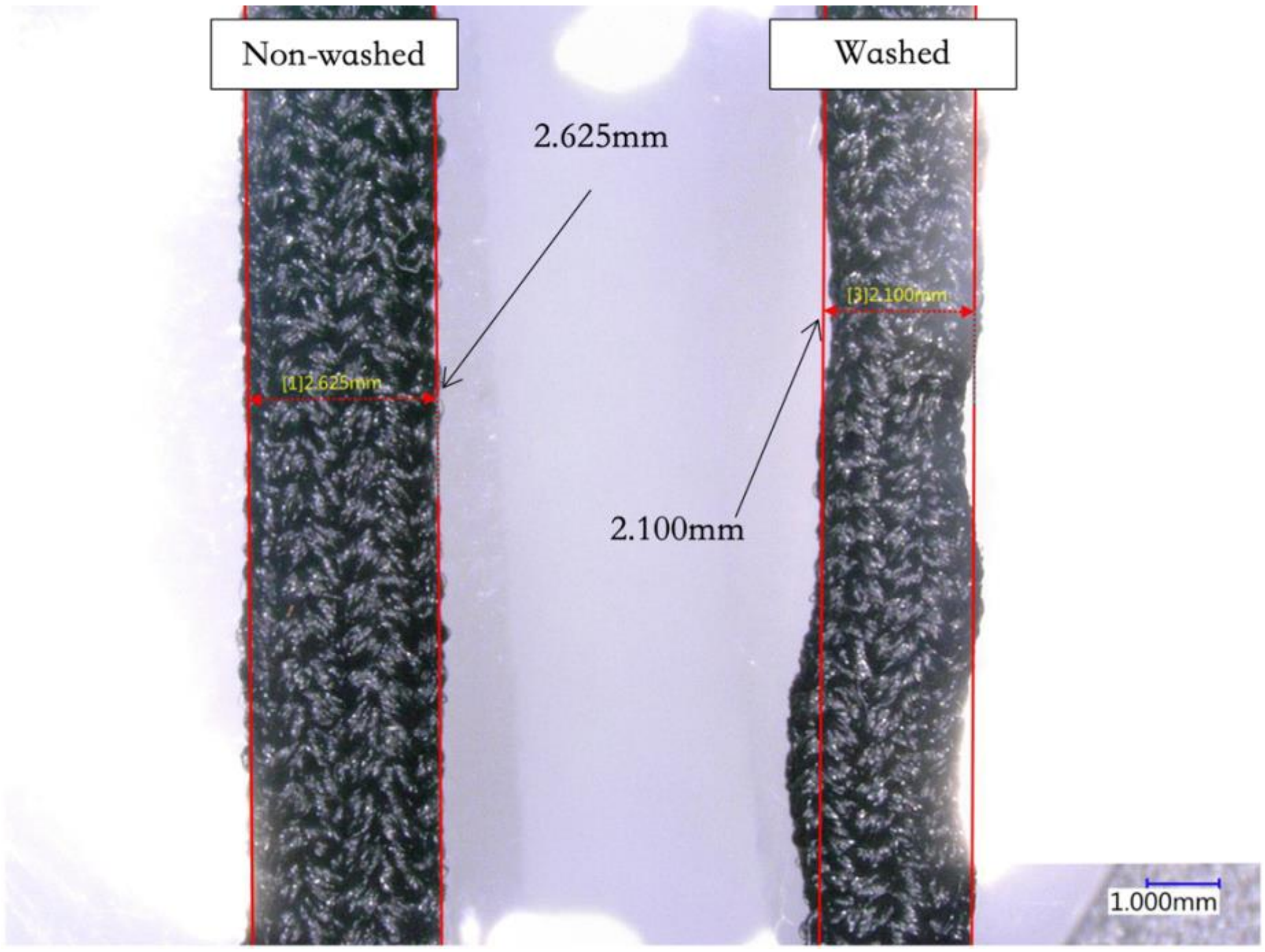

Supplementary Figure 3. Shrinkage of the yarn structure observed in haptic E-yarns after 20 washing cycles.

Engineering Drawing of the Vibration Analysis Test Rig

As discussed in the paper, the engineering drawing of the designed test rig for measuring vibration of the haptic electronic yarn with specific loading conditions is as follows.

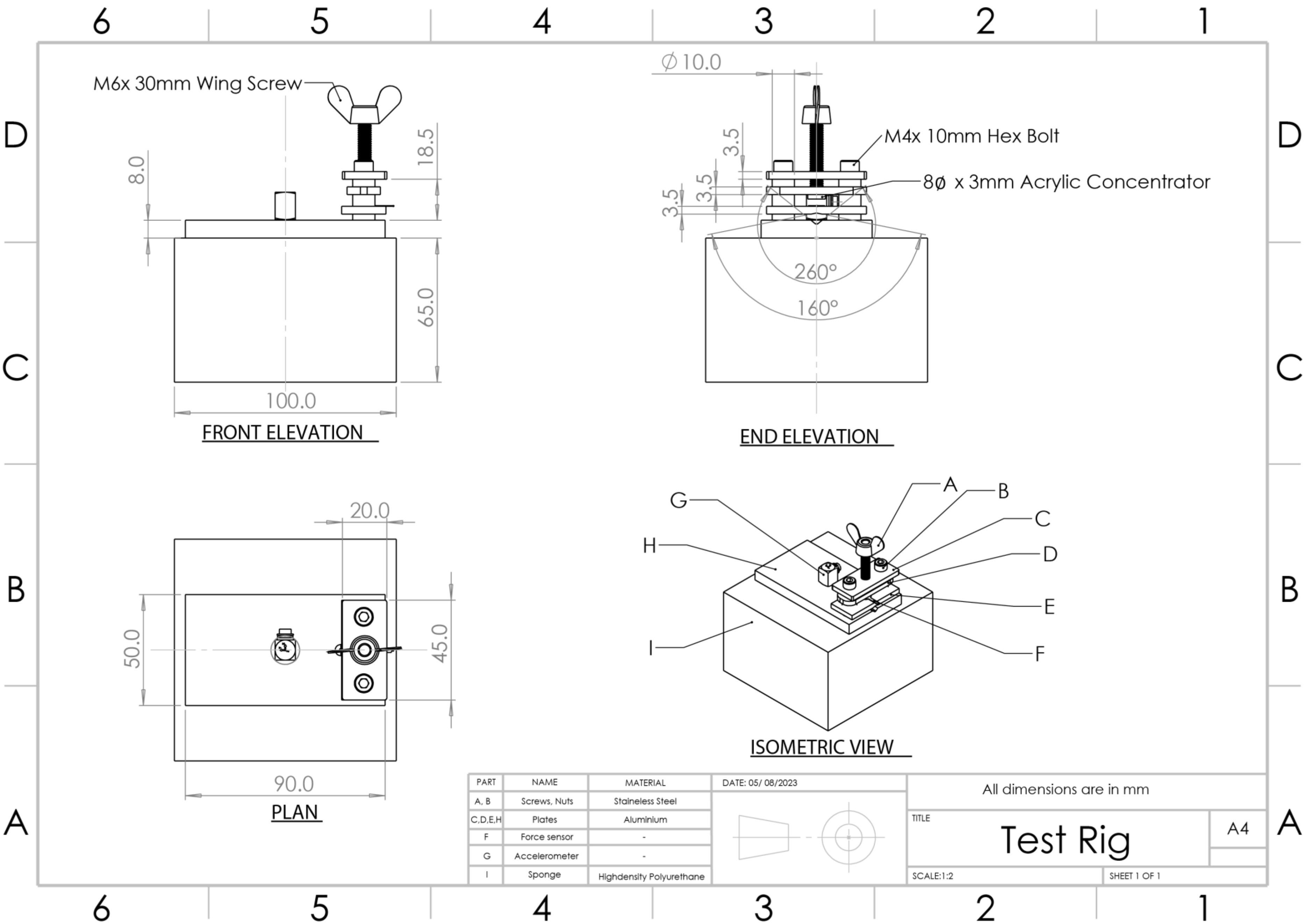

Supplementary Figure 4. Engineering drawing of the designed test rig for analysing vibration of the haptic electronic yarn.

Supplementary Movie 1: Demonstration of haptic E-yarn fabrication, testing, and integration into knitted structures within a wearable glove.

This supplementary video presents the fabrication involved in developing the haptic E-yarn technology. It begins by demonstrating the fabrication of haptic E-yarns using a braiding the micropod encapsulating the haptic actuator. The video then shows how these E-yarns are integrated into knitted textile structures through seamless knitting techniques that create tubular hollow channels designed to house and protect the haptic elements, enabling the construction of a fully functional haptic glove.

Finite Element Analysis (FEA) simulations are briefly introduced to illustrate the predicted stress distributions that occur within the conductive pathways of the E-yarns during bending deformation. Following this, the video demonstrates a custom-built test rig developed to measure and characterize the vibration response of both individual haptic E-yarns and textile structures incorporating these yarns.

The final segment showcases the functional haptic electronic *plain-knit* glove worn on the hand, highlighting its connectivity with external smart devices and its ability to deliver controlled vibrotactile feedback replicating the real-time communication feasibility.

Supplementary Movie 2: Demonstration of the rib knit glove and its vibrotactile functionality.

This supplementary video showcases the vibrotactile performance of the *rib-knit* haptic electronic glove. The demonstration begins with a simple visualization technique in which coffee granules placed on a white A4 sheet positioned over the glove respond to different vibration patterns. The movement of the granules provides an intuitive indication of the vibration amplitude and the spatial characteristics of the haptic feedback produced by the glove.

The video then presents the rib-knit glove worn on the hand, illustrating its flexibility, wearability, and seamless integration of haptic E-yarns within the textile structure. This segment highlights the glove's ability to deliver controlled vibrotactile feedback during natural hand movement.

Wash Report
of Haptic Electronic Yarns and Haptic Electronic Glove

© Malindu Ehelagasthenna 2025

Date: April 8, 2025
Project: Integration Haptic Feedback into Textiles Version

Contents

Purpose 2

Scope 2

Normative references 2

Apparatus and materials 3
X-ray generating MicroCT 3
Automatic washing machine 3
Tumble dryer 3
Reagents 3
Wash bags 3
Line drying 4
Ballast 4
Thermometer and hygrometer 4
Laboratory DC power supply 4
Digital Multimeter 5
Test Rig for Evaluating Mechanical Vibrations 5

Specimens 6

Pretreatment 6

Wash load 6
Total wash load 6

Selection of wash program 6

Washing procedure 7

Drying procedure 8
Line dry 8

Testing after washing and drying 8
Working condition 8
Measurement of electrical resistance and vibration 8

Test results 8
X-ray imaging 11
Summary of the test results 22

Conclusion 23

Contribution statement 23

Purpose

This document is the exclusive property of Nottingham Trent University. This document contains washability of e-textile prototypes conducted by Malindu Ehelagasthenna for his Ph.D under the supervision of Advanced Textile Research Group. This document shows the visibility and transparency of the wash procedure for the public.

Scope

This document demonstrates the domestic wash durability of an engineered haptic electronic yarn and an e-textile prototype of a haptic electronic glove. These test samples include electrically conductive components and electronic actuators to provide vibrotactile haptic feedback for the user.

The test procedure was conducted to align with the wash standard as much as possible and the deviations are listed in this document.

Supplementary references

The wash test was designed with reference to the following standards, and the test procedure was aligned with them as closely as possible. Any deviations from these standards are explicitly stated in this document.

ISO 6330:2021 - Domestic washing and drying procedures for textile testing

This standard outlines washing and drying methods for testing textiles in a household setting. These procedures apply to textile fabrics, garments, and other textile items that undergo various domestic washing and drying processes.

IEC 63203:2023 - Test method for assessing washing durability of e-textile products

This standard defines a domestic washing durability test method for e-textile products. It covers testing procedures for e-textiles with electrically conductive components and sensors designed to collect user data.

ISO 139 - Textiles – Standard atmospheres for conditioning and testing

This standard specifies the standard atmospheric conditions for the conditioning and testing of textiles.

Apparatus and materials

X-ray generating MicroCT

X-ray imaging was utilized to examine the conductive components before and after wash tests, eval-uating the impact of mechanical stress on the samples. A MicroCT Microfocus X-ray system (Model: XT H 225, Nikon, Minato City, Tokyo, Japan; X-ray source: 225kV, 225W reflection) was used for this imaging process.

Automatic washing machine

The reference washing machine, Type A, was selected in accordance with ISO 6330:2021. The specifica-tions of the washing machine (Type A – horizontal axis, front-loading) ((Model: Logixx 8 VarioPerfect, BOSCH, Gerlingen, Baden-Württemberg, Germany)) are provided in **Supplementary Table 4**.

Tumble dryer

A tumble dryer (Model: Classixx 8, BOSCH, Gerlingen, Baden-Württemberg, Germany) was used to immediately remove and dry only the ballasts.

Reagents

The detergent used for testing was Persil Non-Biological Powder, which contains 5-15% anionic sur-factants and oxygen-based bleaching agents, as well as less than 5% nonionic surfactants, optical brighteners, perfume, phosphonates, polycarboxylates, soap, and zeolites. The detergent was ap-plied at a measured dose of (20.9 ± 0.4) g per wash cycle.

Wash bags

Mesh laundry bags (30 cm × 30 cm, made of 100% polyester) in 10 colors were used to keep the samples separated during washing. Each bag features a drawstring with a cord lock to securely close and fasten it.

Position items	Items	Details	BOSCH Logixx 8 VarioPerfect
Inner drum	Diameter	-	480mm
	Depth	-	(320±20)mm
	Net volume	-	58l
	Lifting vanes	Number	3
		Height	50mm
		Length	265mm
		Side length	72.5mm
		Spacing	120°
Outer drum	Diameter	-	-
	Wash	With load and water	50rpm
	Rinsing	With load and water	50rpm
	Hydroextraction	High spin	800rpm
Program	Easy-Care (wash program)	Program for textiles made of synthetic or blended fabrics	27s ON 3s OFF

Supplementary Table 4: Specifications of the washing machine.

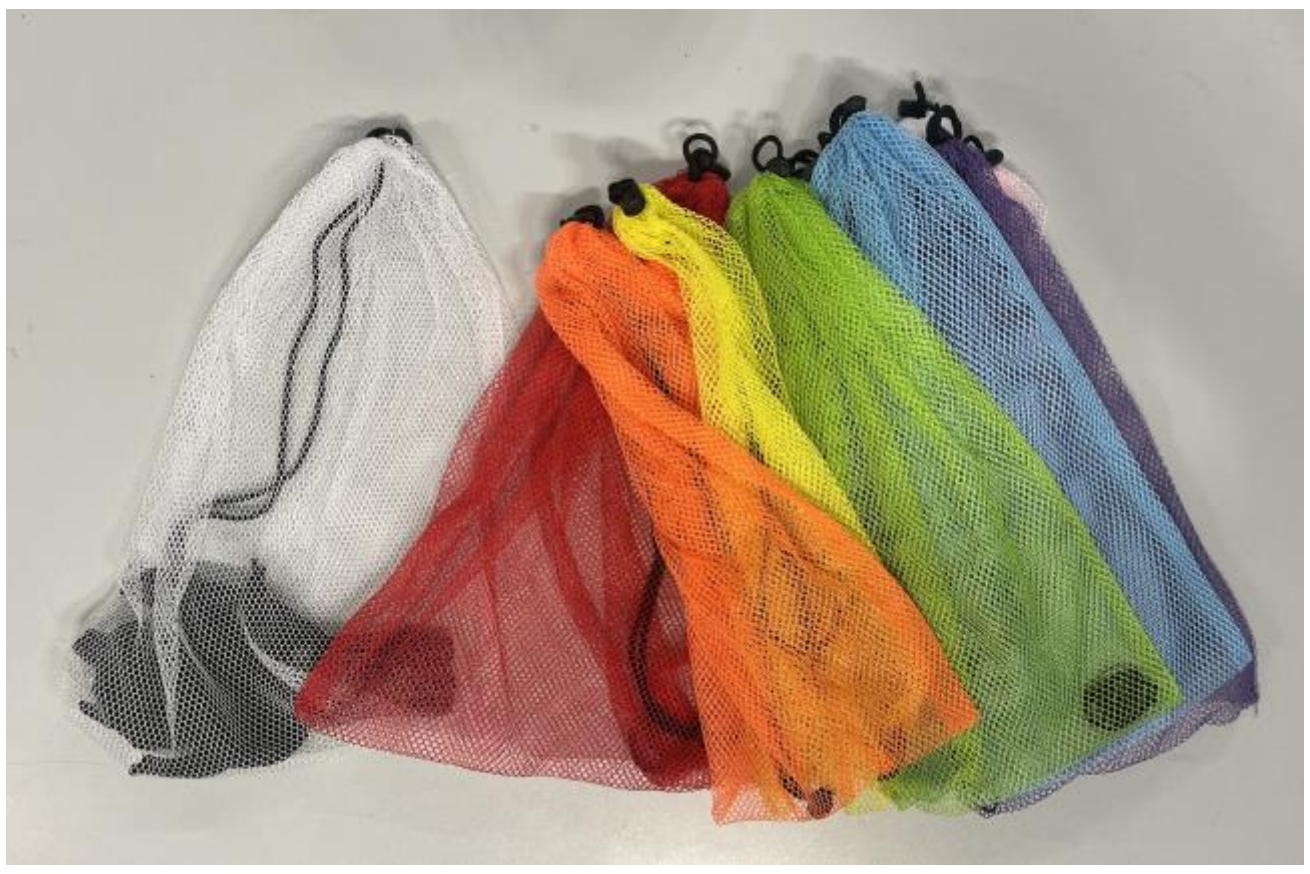

Supplementary Figure 5: Wash bags and samples inside the wash bags

Line drying

Wash bags with cords were used to hang the samples on a wooden rod immediately after washing for air drying. The hanging procedure is shown in **Supplementary Figure 6**.

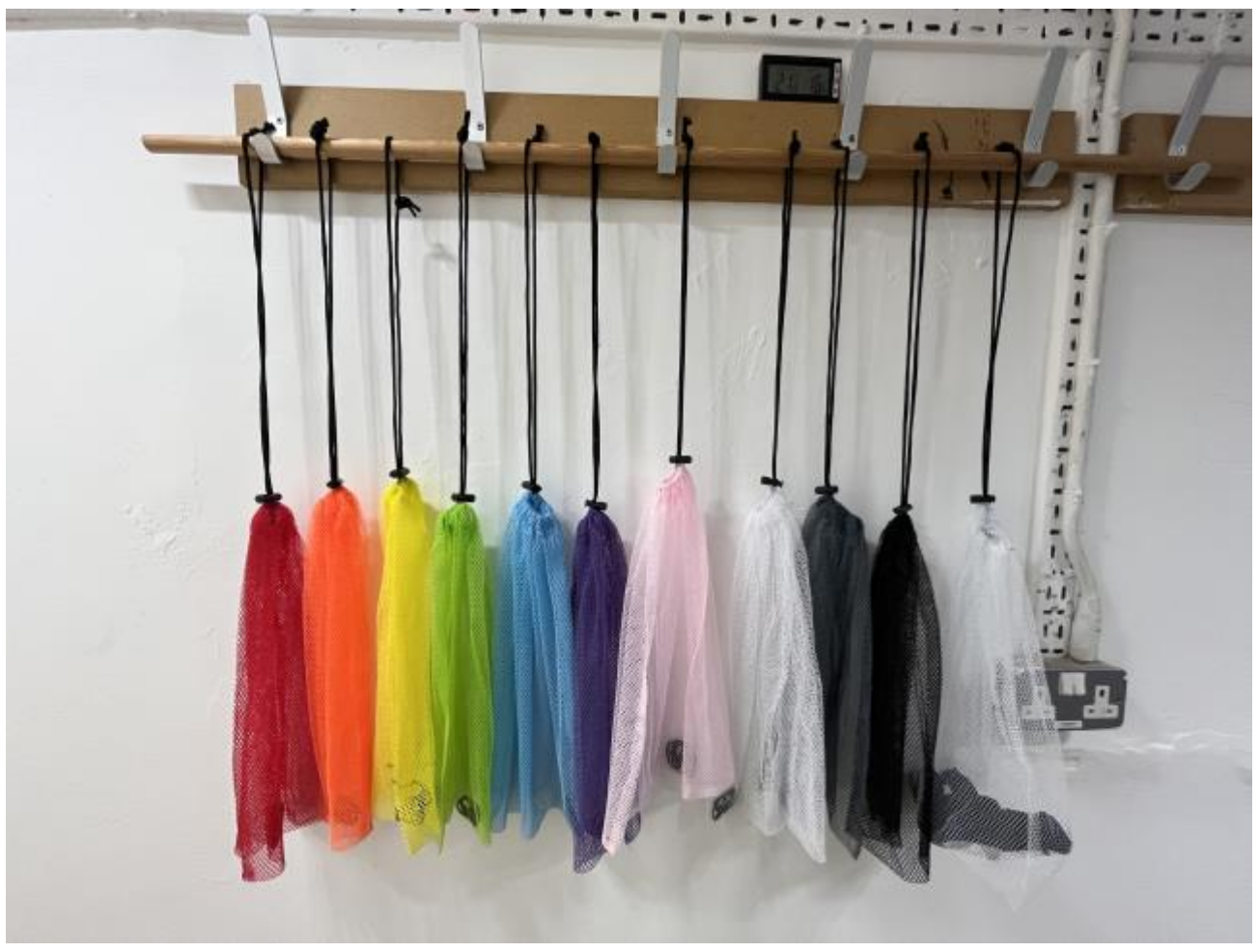

Supplementary Figure 6: Line drying procedure

Ballast

The ballasts were prepared to closely match the Type III ballast specifications as defined in ISO 6330:2021 (see **Supplementary Figure 7).** Detailed specifications for the ballast are provided in **Supplementary Table 5**.

Thermometer and hygrometer

The atmospheric temperature and humidity were measured multiple times at various locations throughout the test using a portable digital thermo-hygrometer.

Laboratory DC power supply

The electronic textile samples were powered using a laboratory DC power supply (IPS 3303, ISO-TECH, Pine Grove, Southport, Merseyside, England, PR9 9AG).

Items	Ballast
Composition	100% Polyester
Fabric Construction	Plain woven fabric
Thread count warp	11 threads/cm
Thread count wef	8 threads/cm
Fabric mass per unit area	290 g/m2
Piece size	(21 ± 1) cm × (21 ± 1) cm
Sewing	over-locked on all four sides
Piece mass	(12.3 ± 0.4) g

Supplementary Table 5: Specification of ballasts

Digital Multimeter

The electric resistance measurements were taken from a digital multimeter (Model no: 34461A 6 1/2 Digital Multimeter, KEYSIGHT, Santa Rosa, California, U.S.)

Test Rig for Evaluating Mechanical Vibrations

The electronic haptic yarns and the haptic glove vibrations were measured using a customized test rig developed for vibration measuring and recording. Vibrations were recorded using a calibrated, tri-axial accelerometer (Model 4520-001; Brüel & Kjær, Nærum, Denmark), connected to a signal analyzer (Photon+; Brüel & Kjær, Nærum, Denmark), and controlled by RT Pro Photon software (v7.30.10; Brüel & Kjær, Nærum, Denmark).

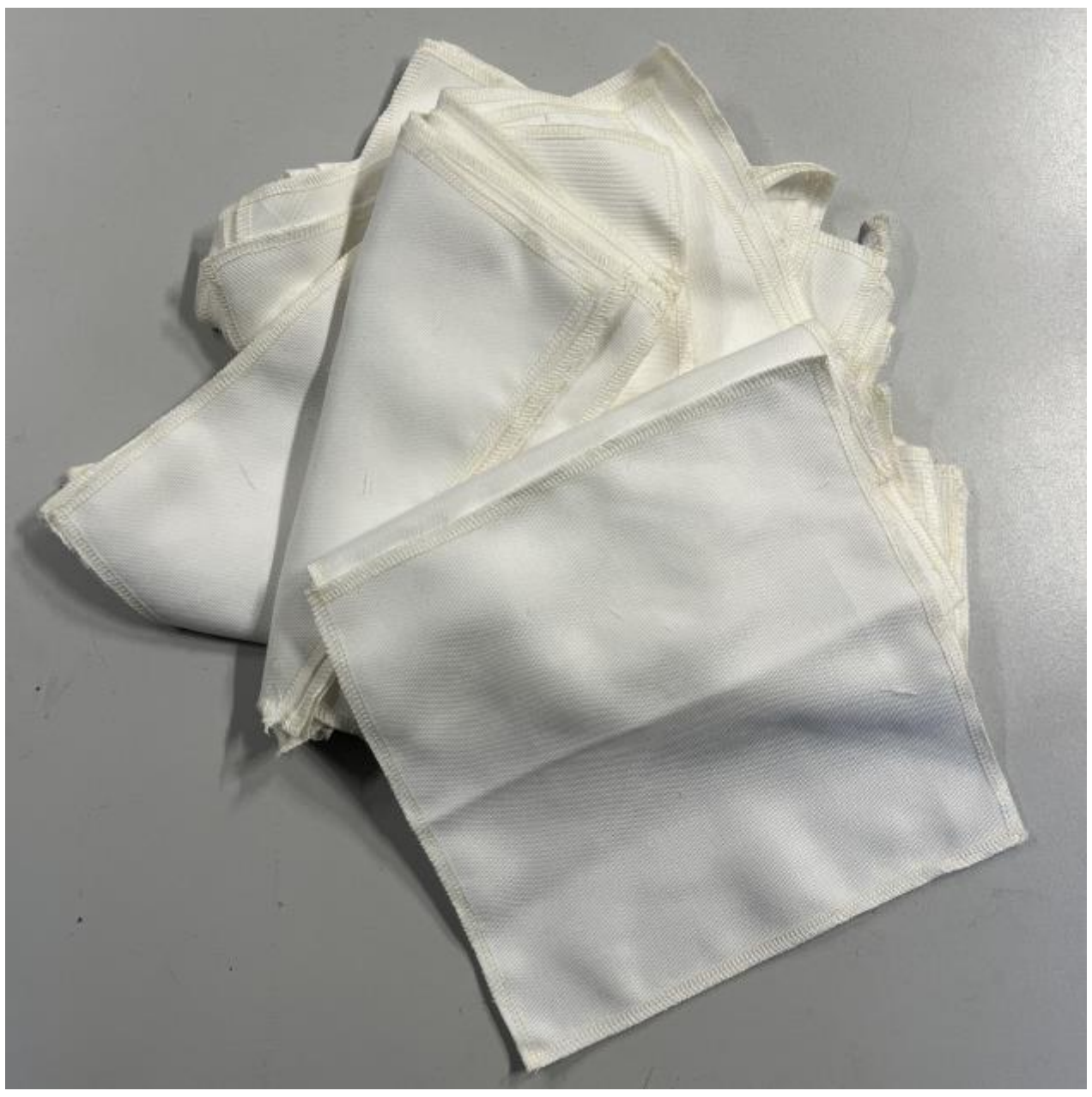

Supplementary Figure 7: Polyester ballast materials used to balance the wash load.

Specimens

A prototype haptic electronic glove embedded with four haptic electronic yarns was washed along with ten additional standalone haptic electronic yarns. Each yarn measured 300mm in length and 2.5mm in diameter.

Pretreatment

The test samples were conditioned in an atmosphere maintained at 19.6^{0}C and 15% relative humidity (RH). The specimens were inspected to ensure proper functionality before testing. All detachable components, such as the control module and battery of the glove prototype, were removed prior to washing. Embedded electronics yarns remained within the prototype throughout the washing durability test.

Wash load

The specimens were washed and dried in accordance with the procedures outlined in ISO 6330:2021 and IEC 63203:2023 standards.

Total wash load

The total air-dry load mass, including the specimen and appropriate ballast, was 2.05kg.

Selection of wash program

The washing program (procedure no. 4N) was selected in accordance with the information provided

Agitation during heating, washing				Normal
**Washing**	**Temperature**	a	C	40
	**Liquour level**	b	mm	100
	**Wash time**	d	min	15
	**Cool down**			No
**Rinse 1**	**Liquour level**	b	mm	130
	**Rinse time**	c	min	3
**Rinse 2**	**Liquour level**	b	mm	130
	**Rinse time**	c	min	3
	**Spin time**	d	min	-
**Rinse 3**	**Liquour level**	b	mm	130
	**Rinse time**	c	min	2
	**Spin time**	d	min	-
**Rinse 4**	**Liquour level**	b	mm	130
	**Rinse time**	c	min	2
	**Spin time**	d	min	5

Supplementary Table 6: Wash programs of the washing machine

in Table B.1 of ISO 6330:2021. The specifications of the wash program can be found in **Supplementary Table 6**.

- Agitation: 27s drum rotation and 3s static. High spin
- [a] : Main wash temperature.
- [b] : The liquor level is measured from the bottom of the cage after the machine has run for 1 minute.
- [c] : A cold rinse is the default program in the machine.
- [d] : The specified times are subject to a tolerance of ± 30 seconds.

Washing procedure

1. Prepare the test specimens, excluding electrical components that incorporate batteries.
2. Record X-ray images of internal conductive parts of each specimen before wash.
3. Set measurement points at 150mm in each conductive line of the haptic electronic yarns, start-ing from the haptic component. Measure the electrical resistance six times at each point and vibration of the yarn. Additionally, measure the resistance six times at the connector pins and vibration of each embedded haptic electronic yarn in the prototype glove.
4. Pre-treat the specimens in an atmosphere maintained at a temperature of 19.6°C and a relative humidity (RH) of 15%.
5. Insert each specimen into a separate coloured wash bag—using 10 different colours for the 10 haptic electronic yarns, and a white wash bag for the prototype glove.
6. Add (20.9 ± 0.4) g of detergent directly into the dispenser.
7. The specimen and ballast were evenly mixed and loaded into the washing machine.
8. Select the wash program and temperature according to **Supplementary Table 6.**
9. After the washing procedure is complete, carefully remove the specimens, ensuring they are neither stretched nor distorted, and dry according to the following drying procedure.
10. Temperature and the relative humidity is recorded in 3 locations of the test room in drying state and average is calculated.
11. Operation status of each specimen is tested after drying in each wash cycle using the laboratory DC power supply.
12. The electrical resistance and the vibration of the specimens are measured after each 10 wash cylces.
13. 20 wash cycles are performed throughout the washing procedure.
14. Record X-ray images of internal conductive parts of each specimen after 20 washes.

Drying procedure

At the end of the washing procedure, promptly remove the material and proceed with the line drying procedure.

Line dry

Remove the specimen from the washing machine and hang each hydro-extracted wash bag unfolded to prevent distortion. Suspend the wash bags with the specimens being tested from a wooden rod in still air. For subsequent testing, the drying was performed at a temperature of (22.1 ± 1.4)°C and a relative humidity (RH) of (11.8 ± 2.6)% (see **Supplementary Figure 6**).

Testing after washing and drying

Working condition

After each wash cycle, the specimens undergo a drying process to ensure they are in a state suitable for further evaluation. Once dried, the operation status of each specimen was tested to ensure that it continues to function correctly after exposure to the washing procedure. This was done using a laboratory DC power supply.

Measurement of electrical resistance and vibration

After each wash cycle, the electrical resistance of the yarn and the prototype glove was measured six times at each designated point along the conductive lines, as well as the vibration characteristics of the yarn.

Test results

Specimen number:

- haptic electronic yarns: SWa1, SWa2, SWa3, SWa4, SWa5, SWa6, SWa7, Swa8, SWa9, SWa10
- Embedded yarns insdie the prototype glove: SWaf1, SWaf2, Swaf3, SWaf4

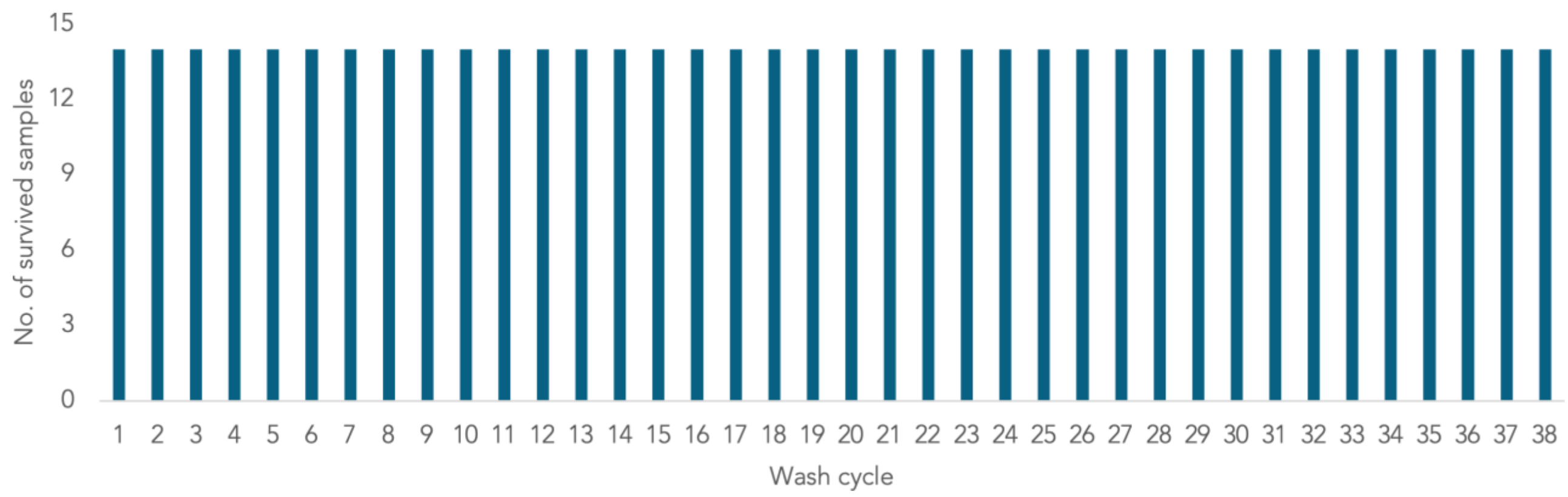

Supplementary Figure 8: Number of survived specimens after each wash cycle

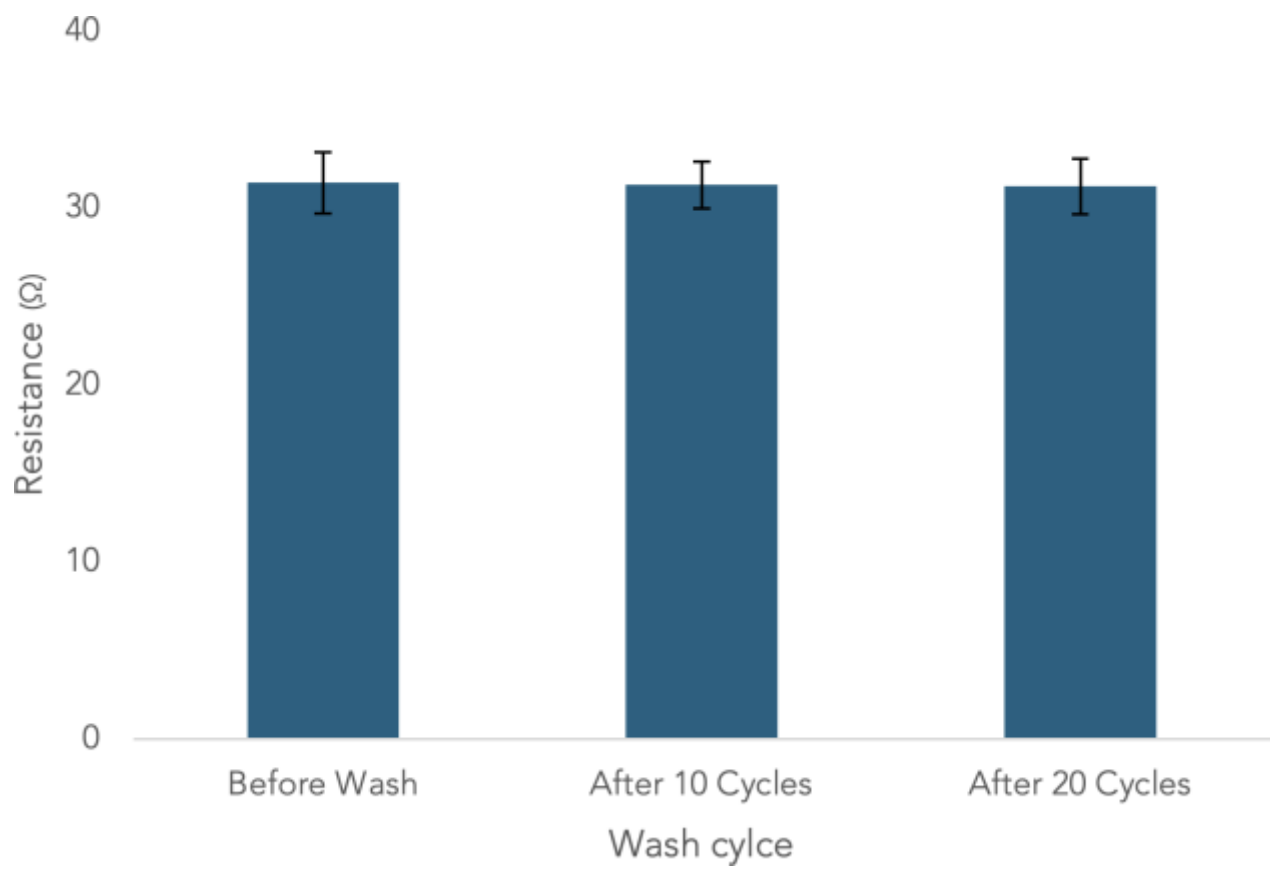

Supplementary Figure 9: Electrical resistance of the haptic electronic yarns after each 10 wash cycle.

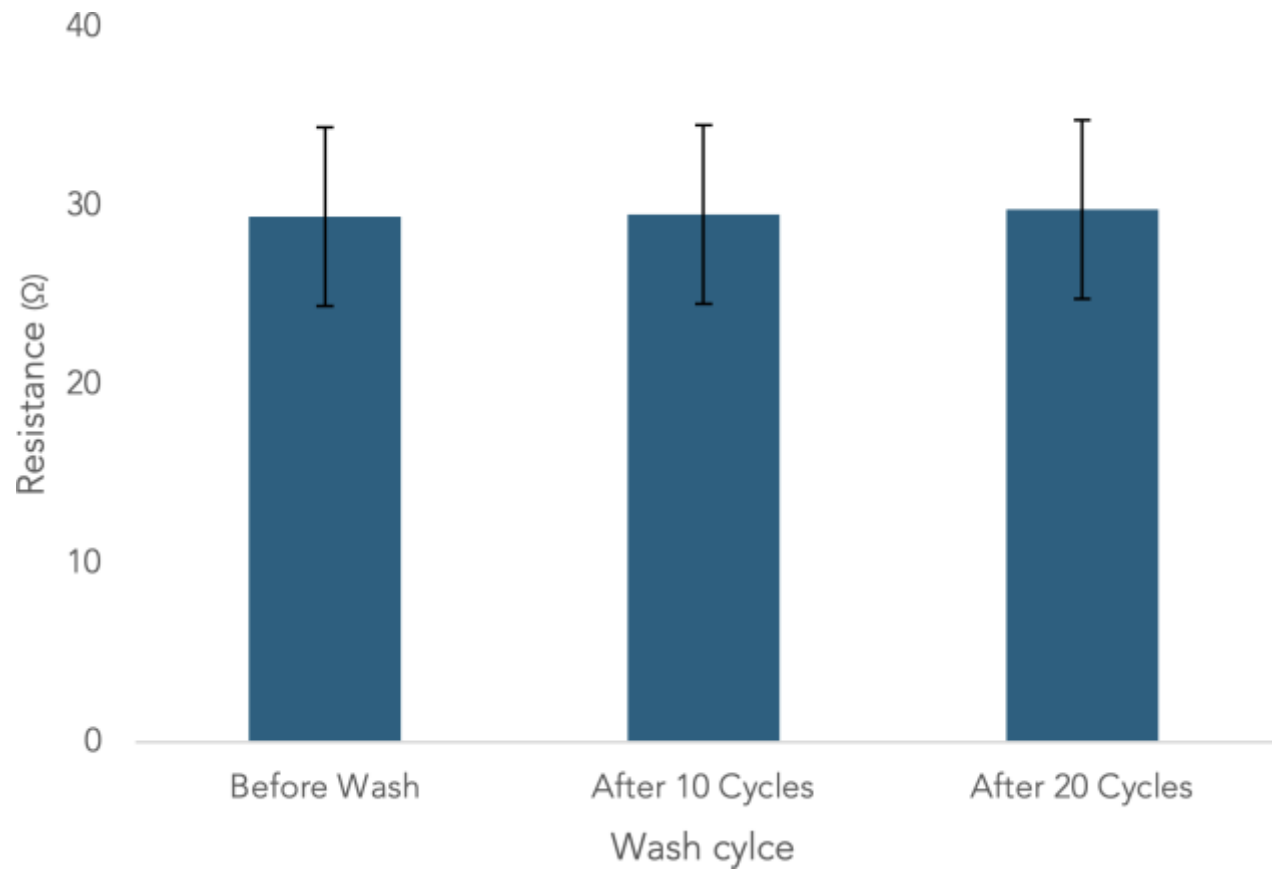

Supplementary Figure 10: Electrical resistance of the haptic electronic yarns inside of the prototype glove after each 10 wash cycle.

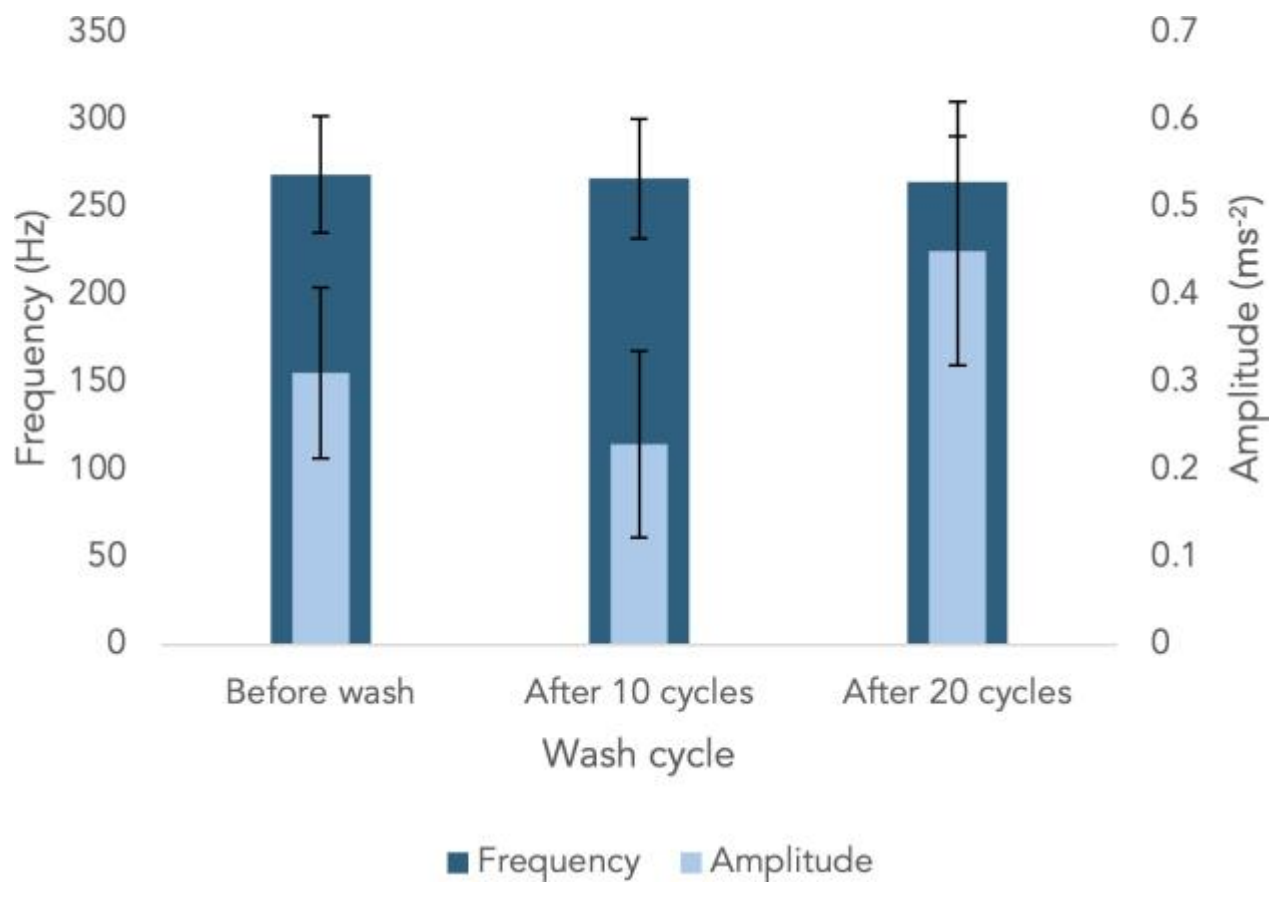

Supplementary Figure 11: Impact to the vibration of the haptic electronic yarns after washing.

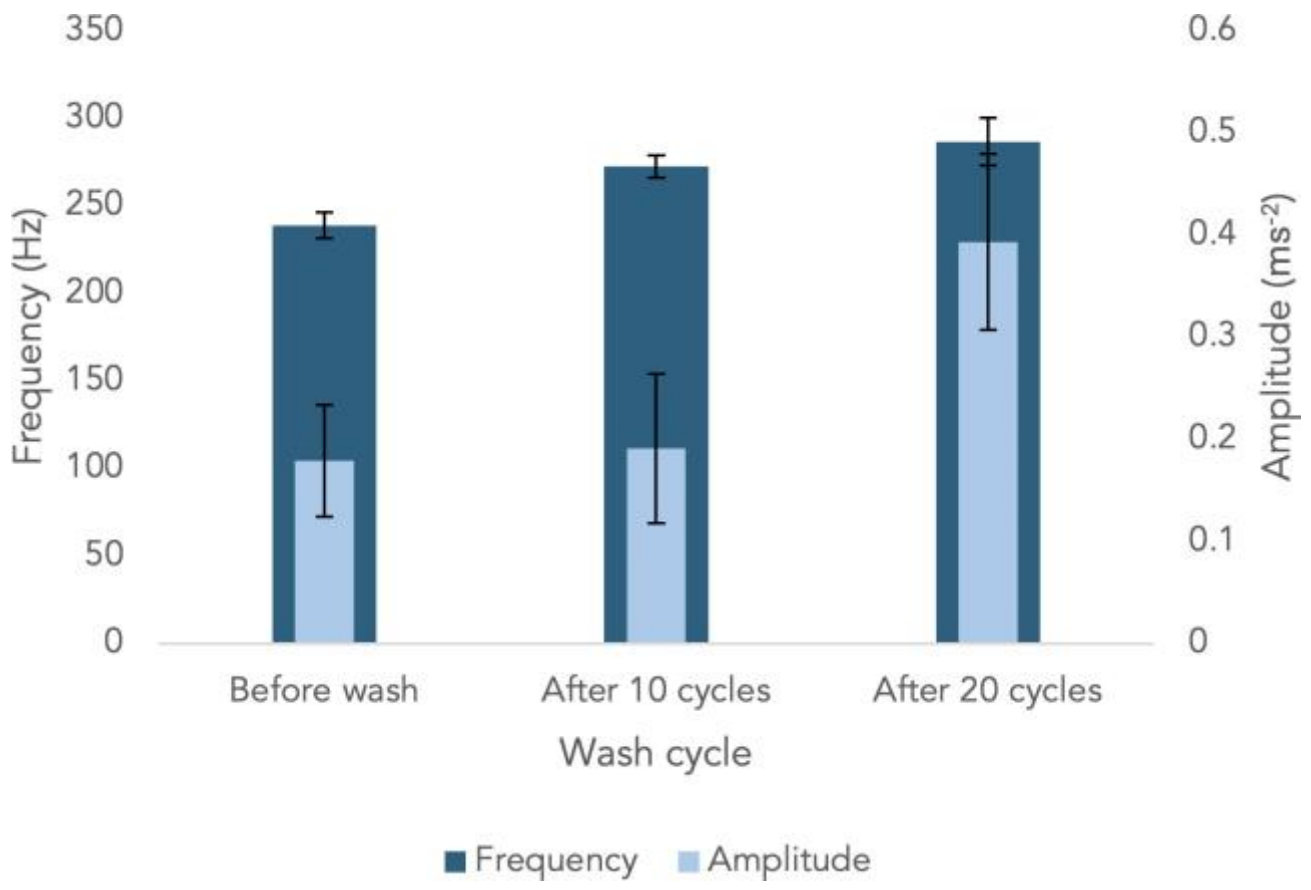

Supplementary Figure 12: Impact to the vibration of the haptic electronic yarns inside the prototype glove after washing.

X-ray imaging

X-ray images were captured before and after the 20th wash cycle to assess the impact of repeated washing on the conductive components of the electronic yarns. Imaging was conducted at two mag-nifications: 1× and 6.3×, respectively (see **Supplementary Figure 13**). All specimens were imaged under identical con-ditions to ensure consistency and comparability across samples.

X-ray images of the prototype glove were also captured in the same manner, with additional focus on the pin connector. These specific images are shown in **Supplementary Figure 14.**

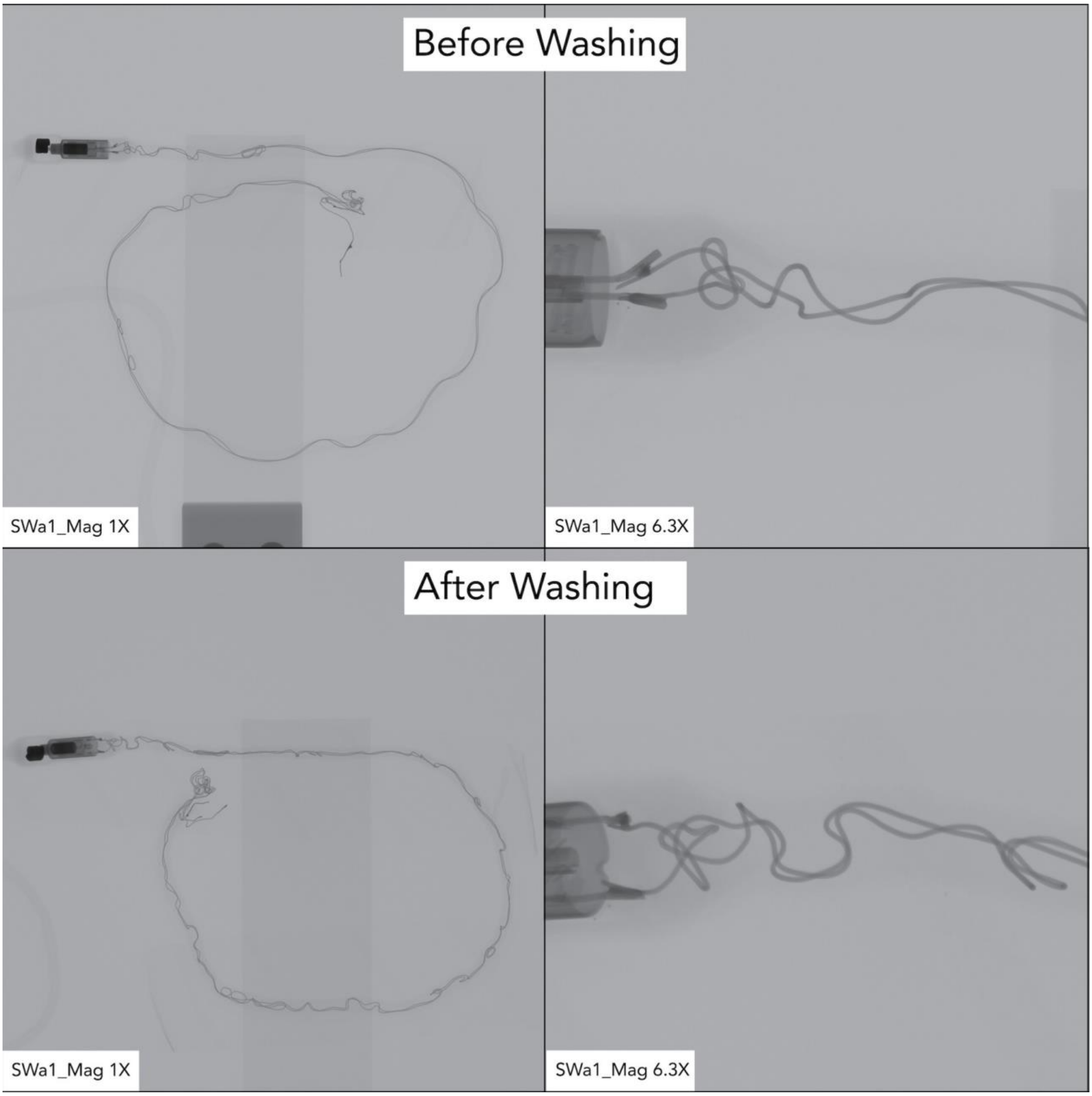

Supplementary Figure 13: X-ray imaging of the conductive lines of the haptic electronic yarn (Specimen no.: SWa1) was performed both before and after 20 wash cycles.

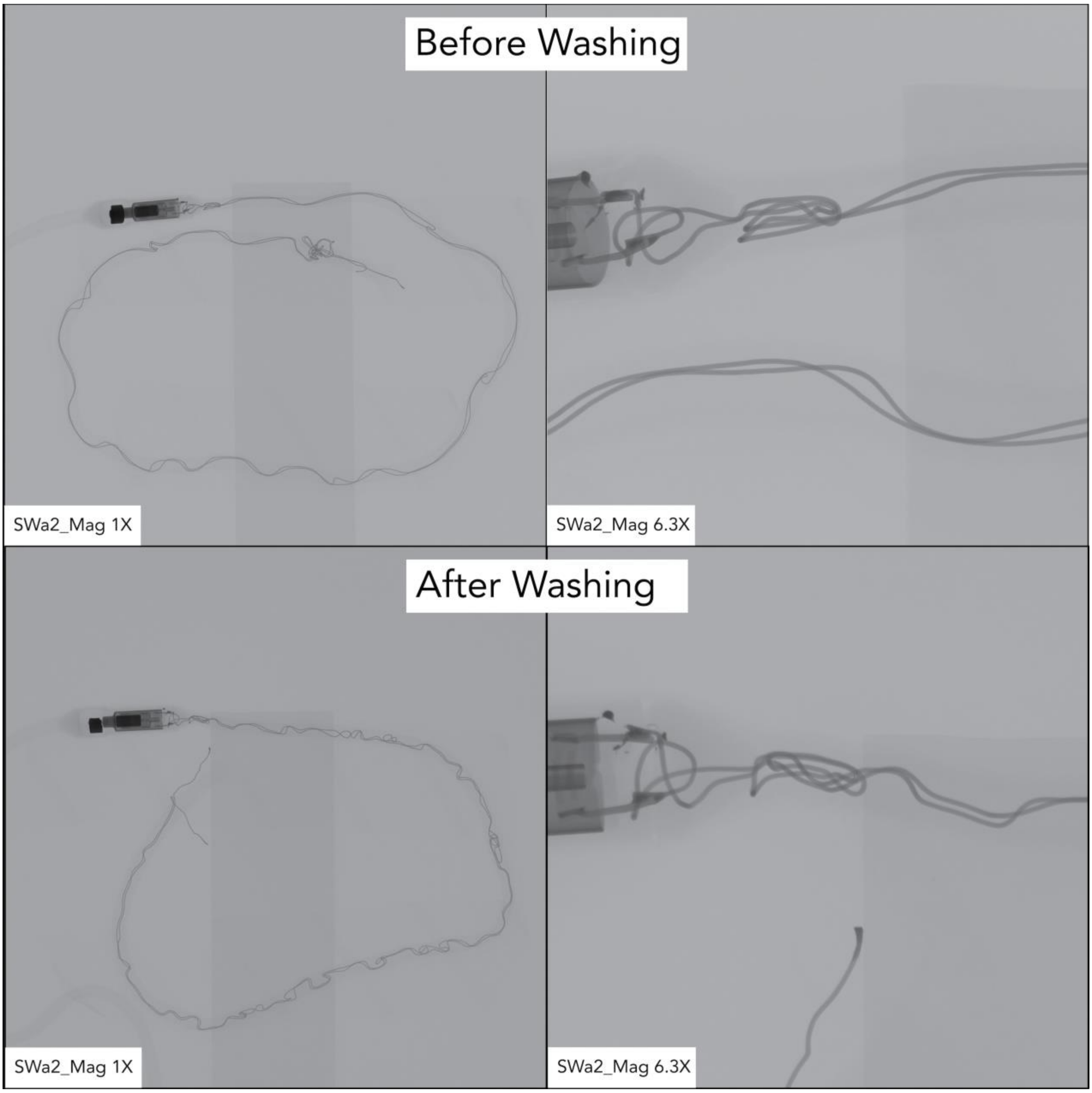

Supplementary Figure 14: X-ray imaging of the conductive lines of the haptic electronic yarn (Specimen no.: SWa2) was performed both before and after 20 wash cycles.

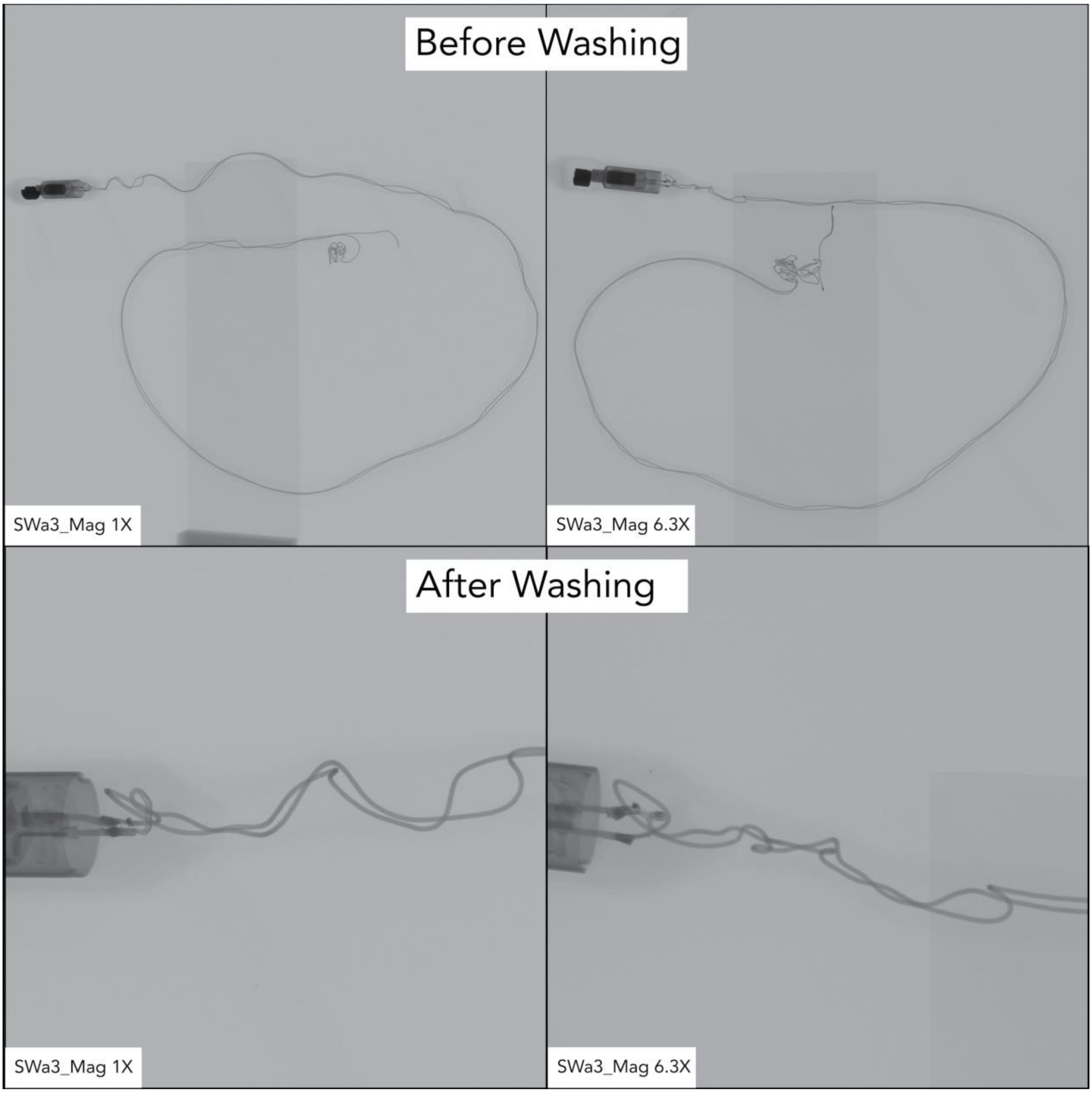

Supplementary Figure 15: X-ray imaging of the conductive lines of the haptic electronic yarn (Specimen no.: SWa3) was performed both before and after 20 wash cycles.

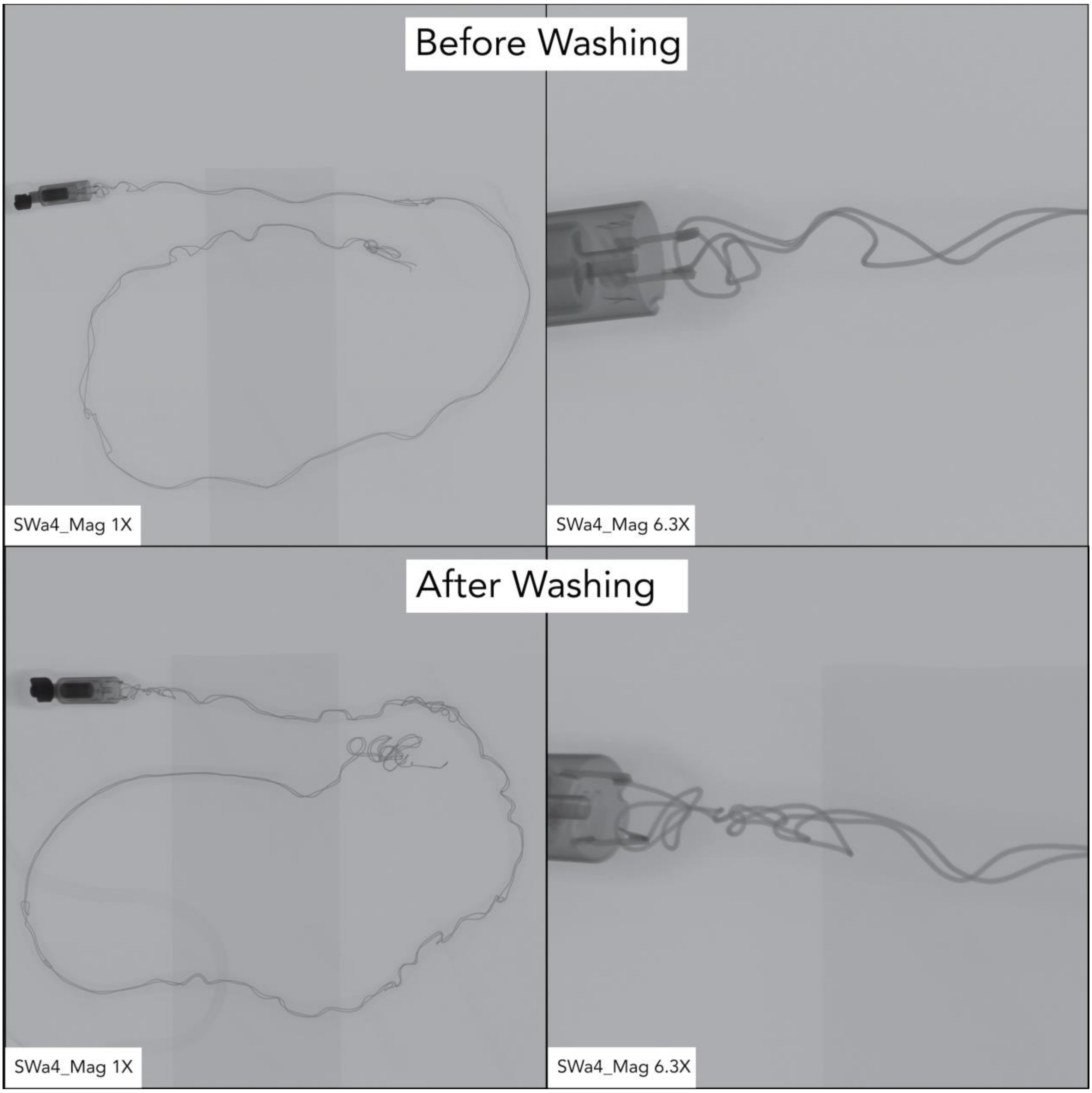

Supplementary Figure 16: X-ray imaging of the conductive lines of the haptic electronic yarn (Specimen no.: SWa4) was performed both before and after 20 wash cycles.

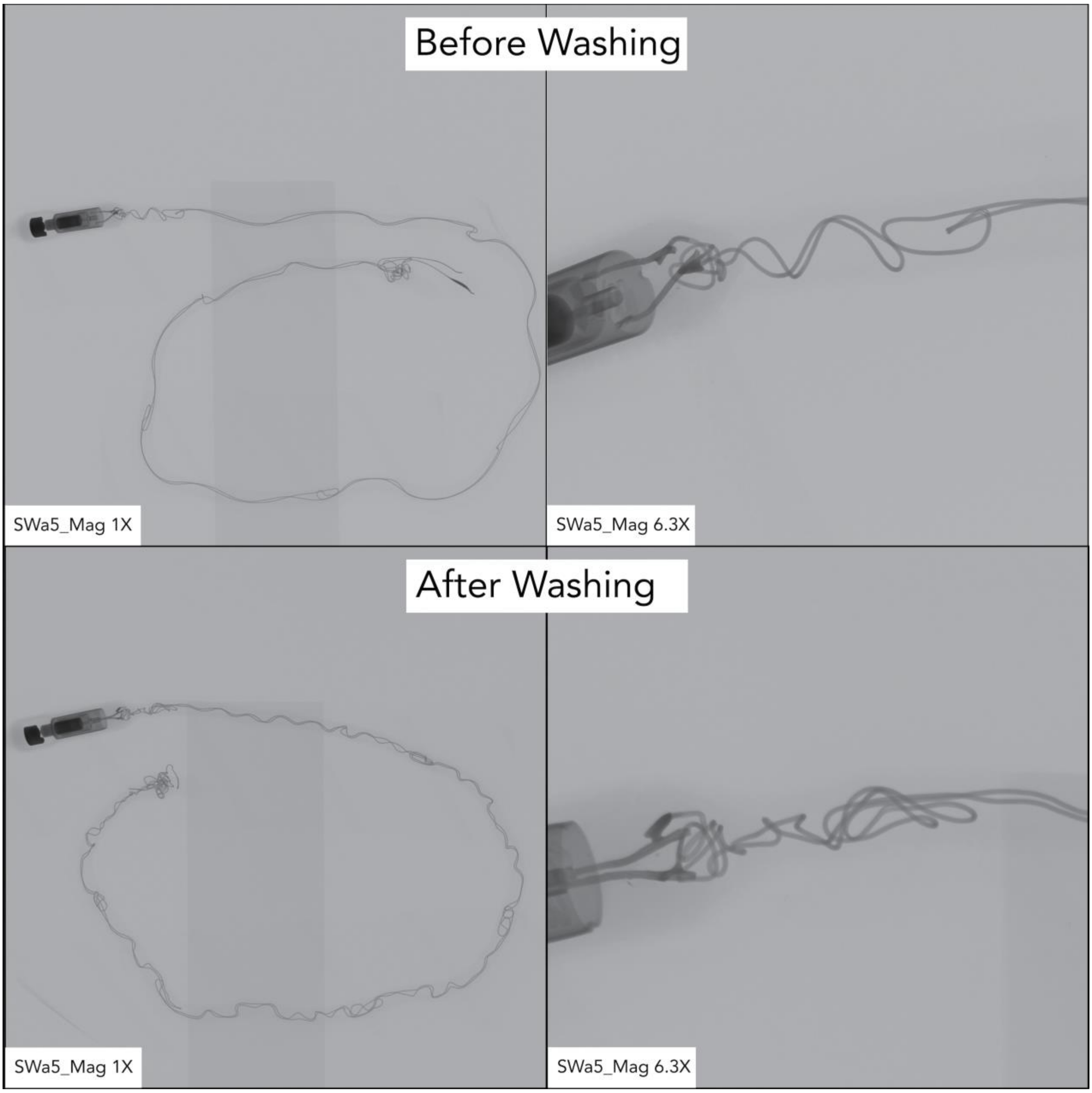

Supplementary Figure 17: X-ray imaging of the conductive lines of the haptic electronic yarn (Specimen no.: SWa5) was performed both before and after 20 wash cycles.

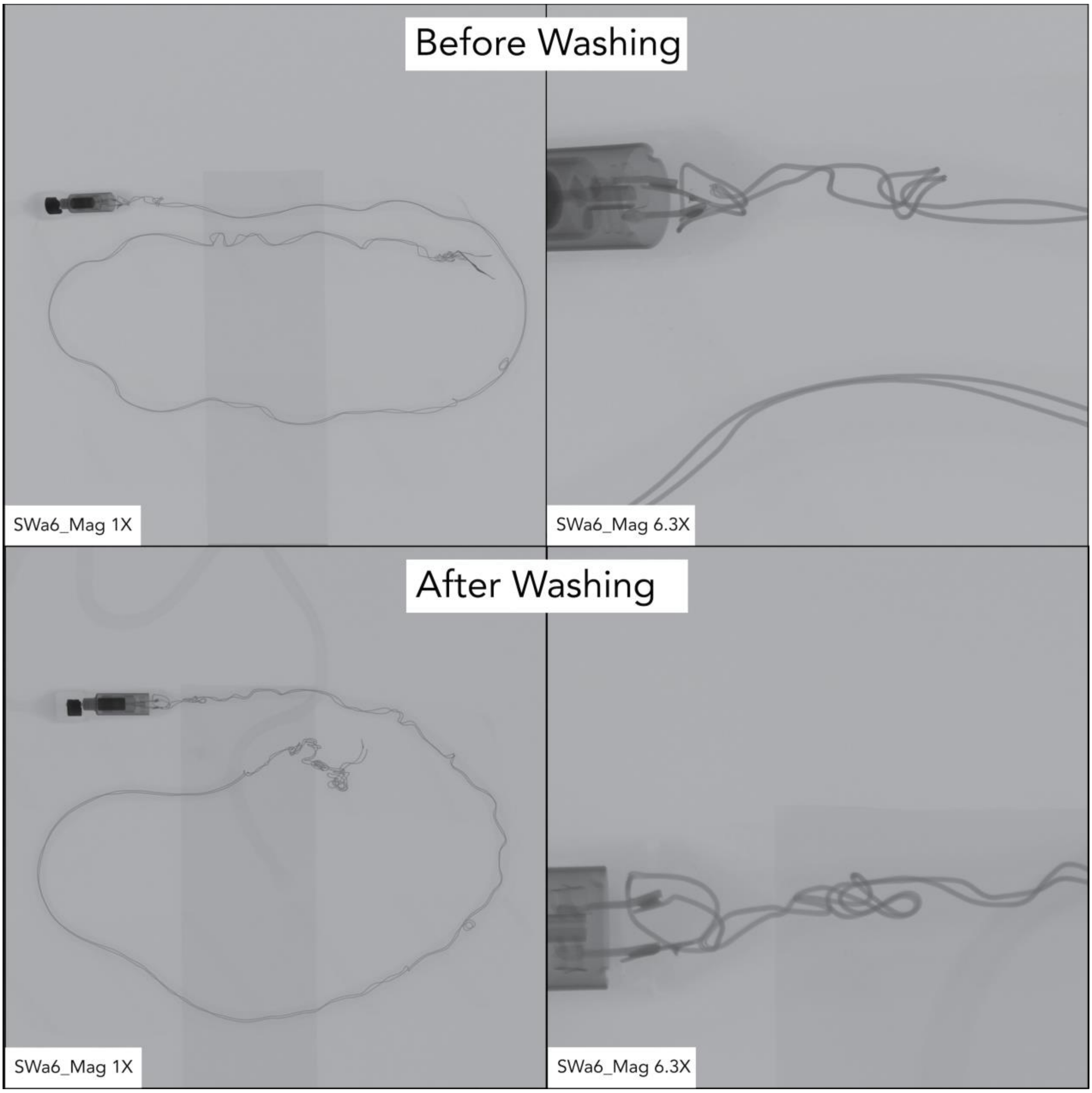

Supplementary Figure 18: X-ray imaging of the conductive lines of the haptic electronic yarn (Specimen no.: SWa6) was performed both before and after 20 wash cycles.

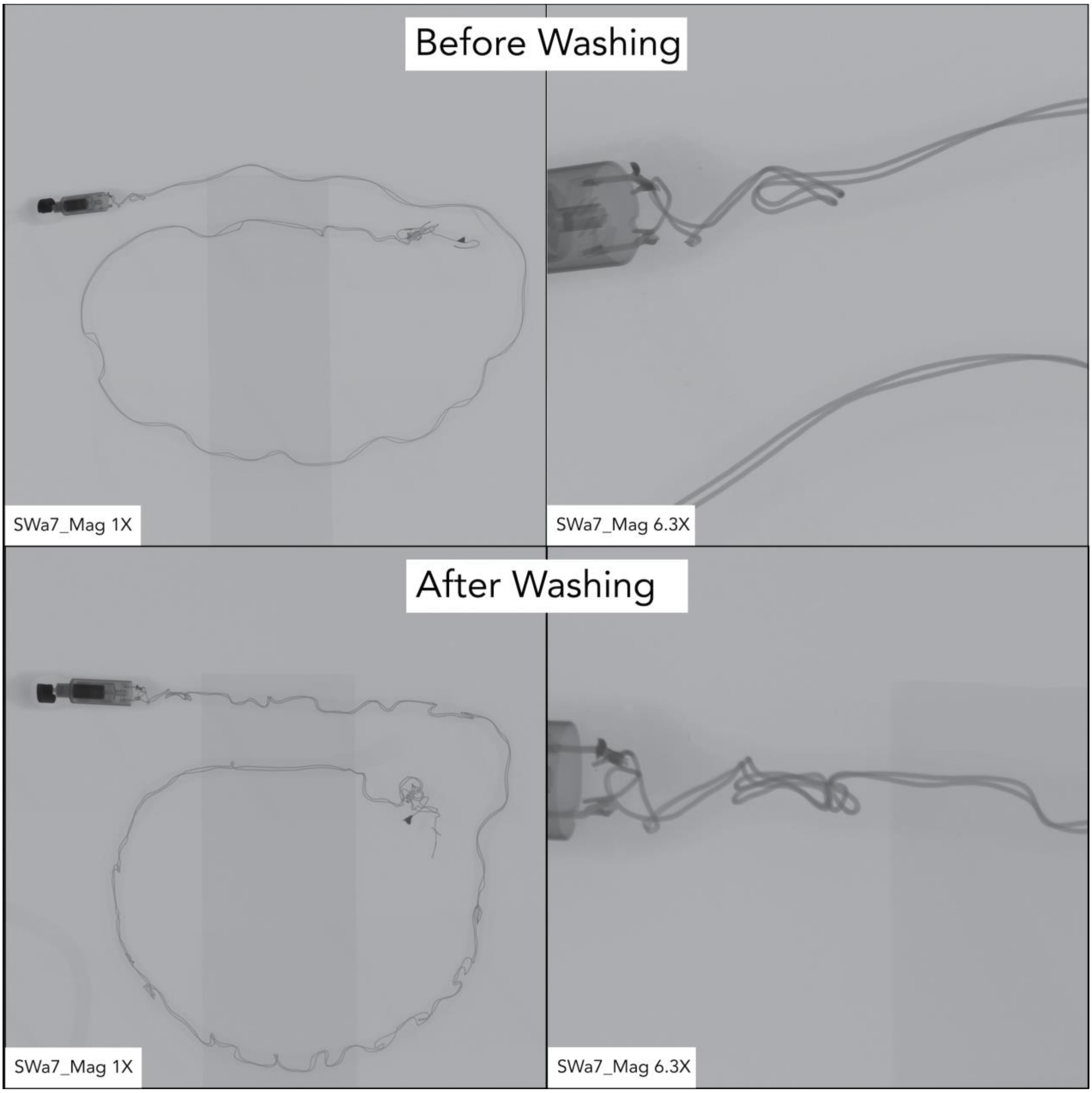

Supplementary Figure 19: X-ray imaging of the conductive lines of the haptic electronic yarn (Specimen no.: SWa7) was performed both before and after 20 wash cycles.

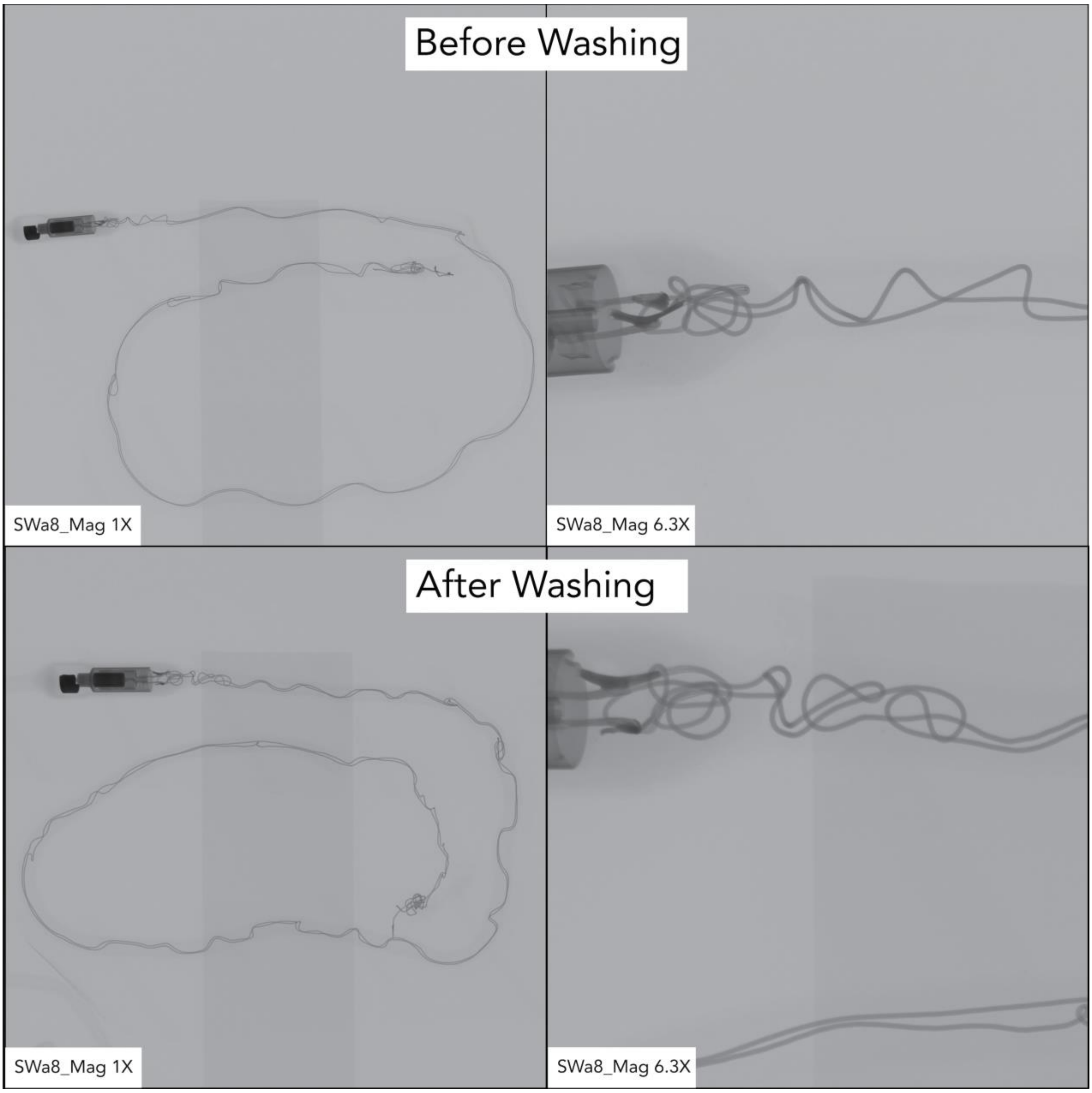

Supplementary Figure 20: X-ray imaging of the conductive lines of the haptic electronic yarn (Specimen no.: SWa8) was performed both before and after 20 wash cycles.

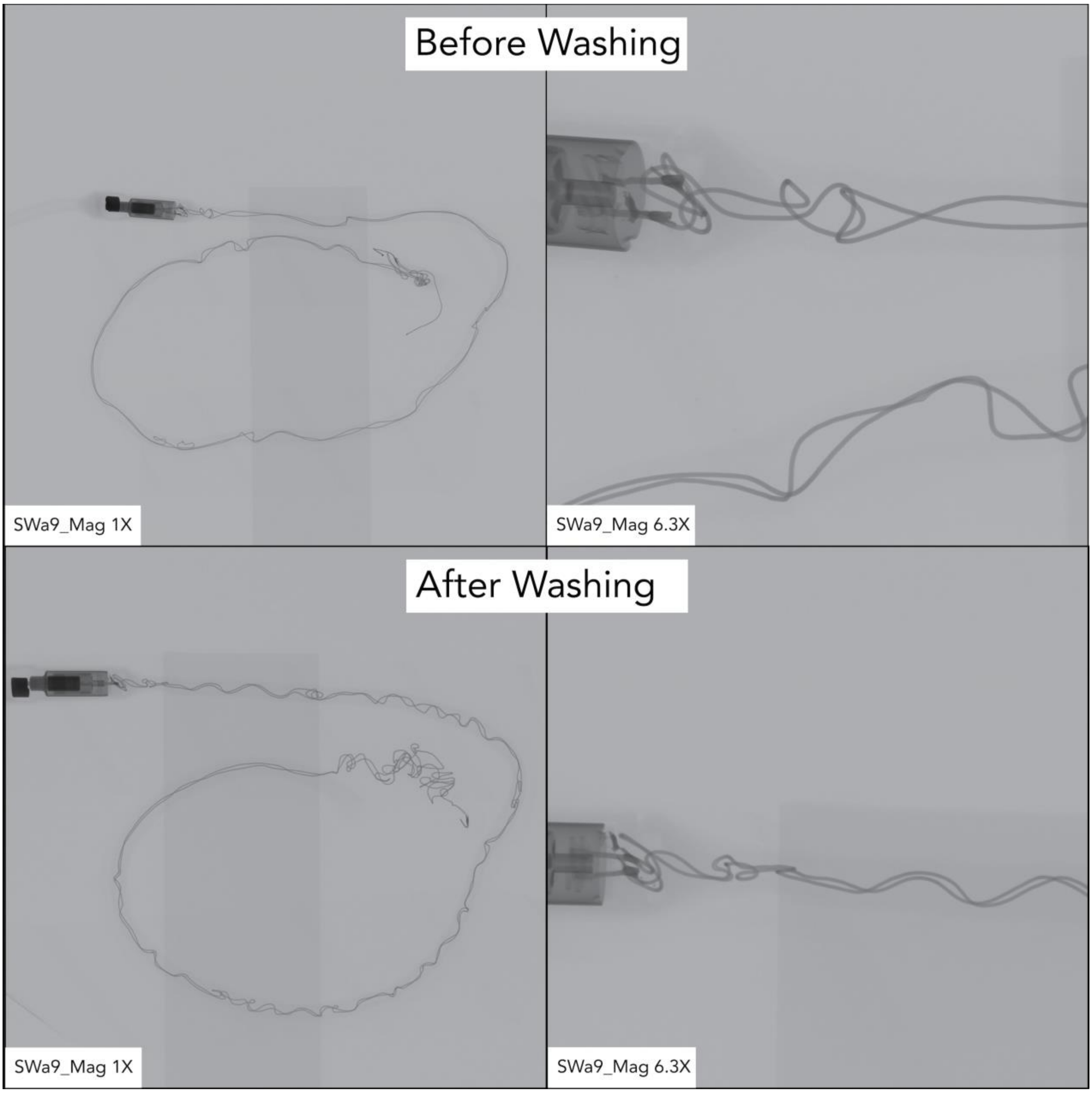

Supplementary Figure 21: X-ray imaging of the conductive lines of the haptic electronic yarn (Specimen no.: SWa9) was performed both before and after 20 wash cycles.

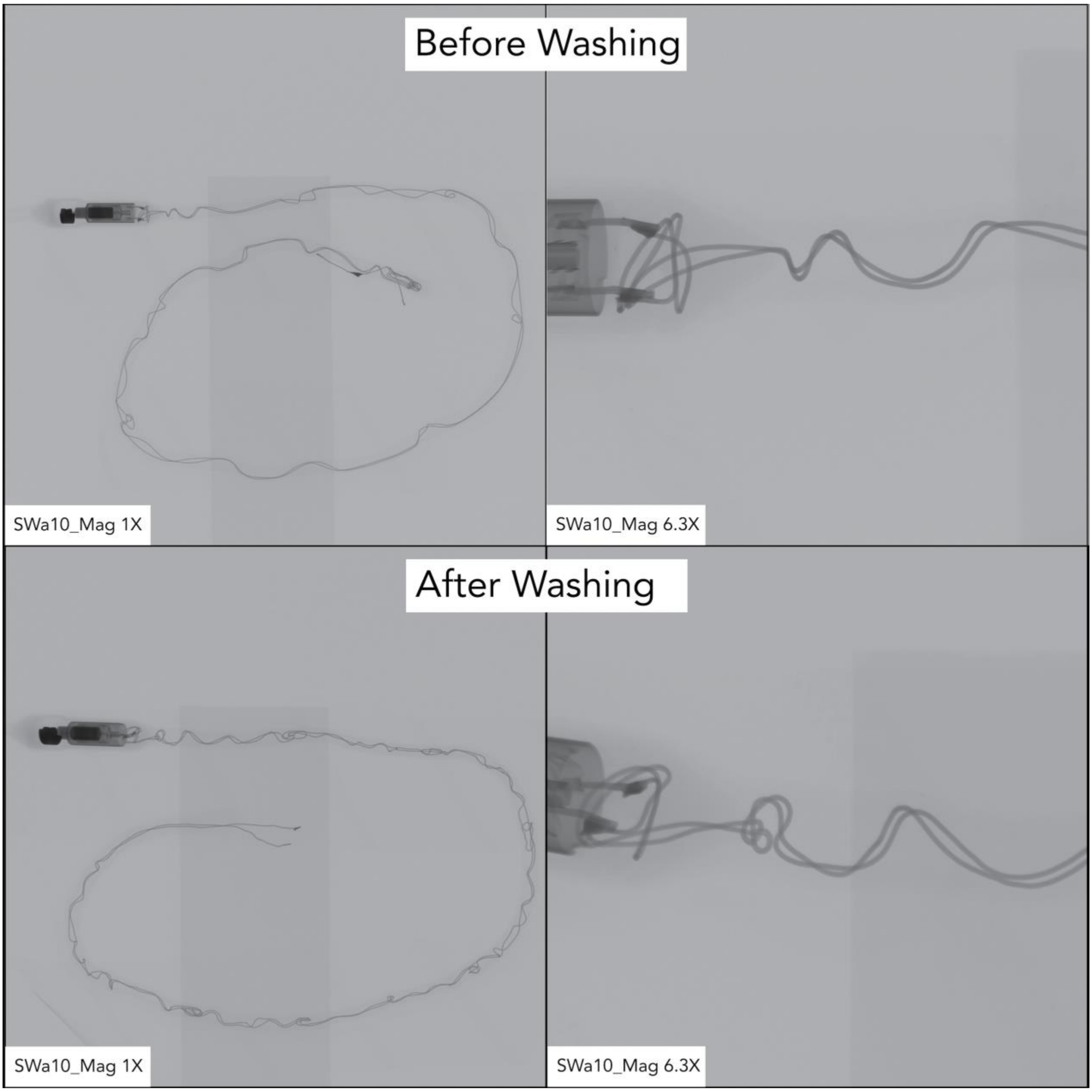

Supplementary Figure 22: X-ray imaging of the conductive lines of the haptic electronic yarn (Specimen no.: SWa10) was performed both before and after 20 wash cycles.

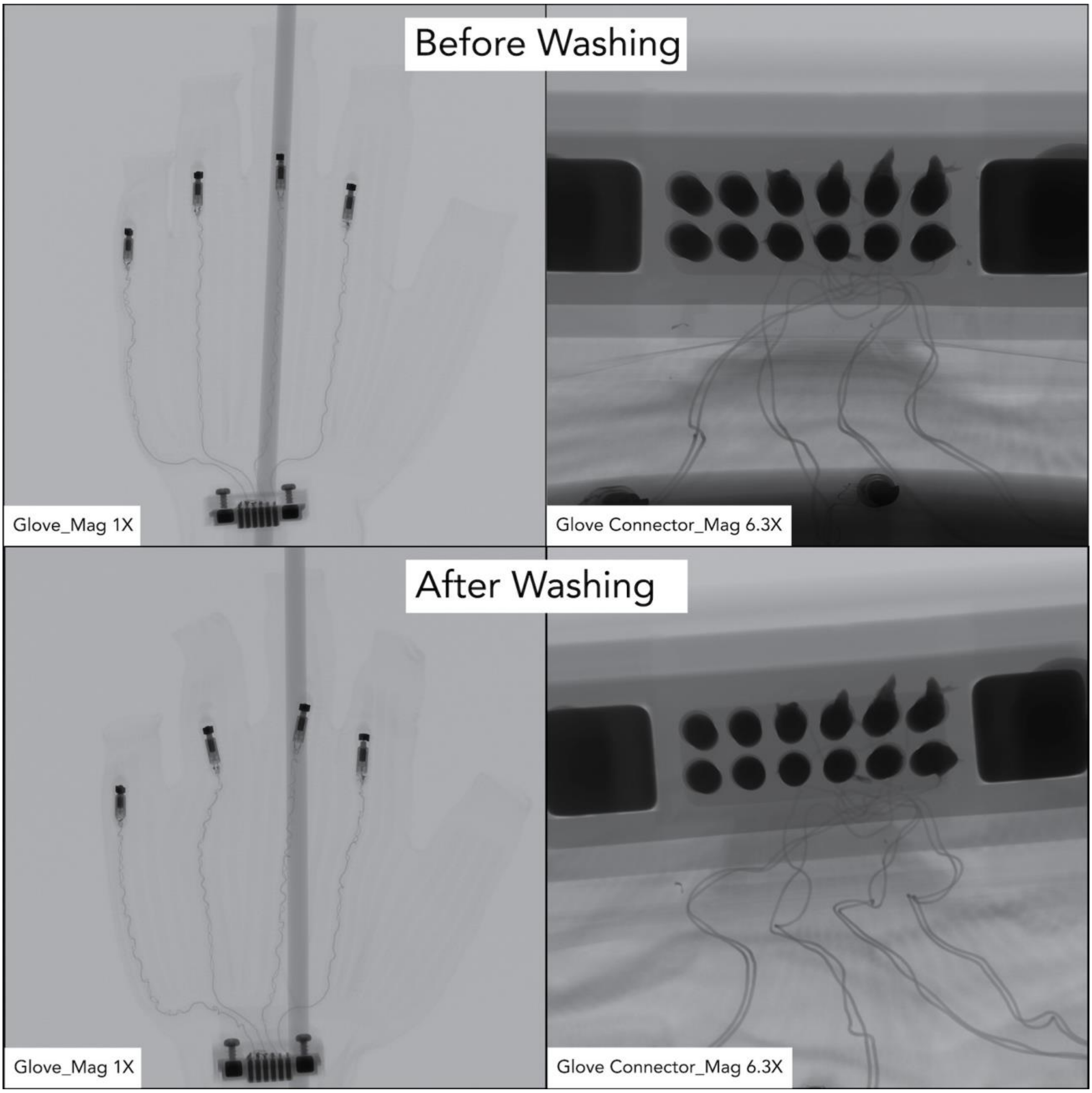

Supplementary Figure 23: X-ray imaging of the conductive lines of the haptic electronic yarns (Specimen no.: SWaf1, SWaf2, SWaf3, SWaf4) embedded in prototype glove were performed both before and after 20 wash cycles.

Summary of the test results

No visible internal or external damage was observed after 20 wash cycles. However, a notable de-gree of deformation in the conductive wires was evident in the X-ray images captured post-washing. The conductive elements exhibited increased loop-like deformation patterns, and slight untwisting was observed at certain locations along the multi-strand twisted conductive wires. Despite these

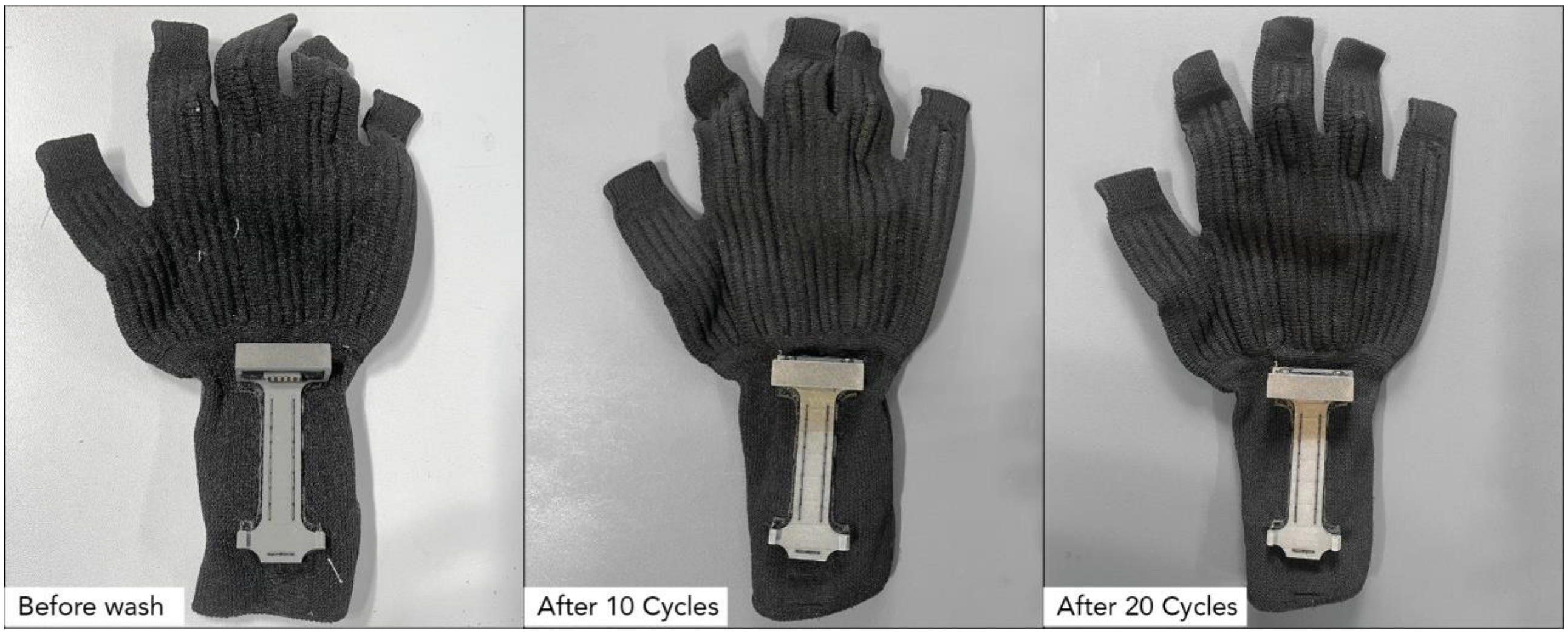

Supplementary Figure 24: Images of the prototype glove after each step of the washing procedure. Note that colour accuracy and white balance may vary between photos.

structural changes, all soldered joints remained intact and securely in place.

Conclusion

This report documents the test procedure for washing electronic textile prototypes developed by the Advanced Textile Research Group at Nottingham Trent University as part of the Ph.D. research of Malindu Ehelagasthenna. The results following 20 wash cycles—each consisting of 15 minutes of washing, 10 minutes of rinsing, and 5 minutes of spinning—indicated no failures in the samples.

Contribution statement

Malindu Ehelagasthenna conducted the test procedure and was responsible for overseeing the entire testing process, including specimen preparation, data collection, analysis, and documentation. Parvin Ebrahimi and Tharushi Pieris assisted with the preparation of the ballasts. Carlos Oliveira contributed to the braiding and knitting of the specimens. Dominic Eberl-Craske supported recording X-ray images. James Meadwell was responsible for the 3D printing of the prototypes. Theodore Hughes-Riley, Richard Bib, Arash Shahidi, and Pasindu Lugoda provided the necessary facilities and guidance throughout the test procedure, ensuring smooth execution and valuable support.

END OF WASH REPORT

Power Consumption Analysis and Battery Life Estimation

Experimental characterisation confirmed that each haptic motor draws a current of approximately 40 mA during active driving (refer **Supplementary Figure 2**).

The optimum stimulation parameters identified by the haptic perception study are a pulse width of T= 10 ms per stimulus and an inter-stimulus interval of T = 250 ms. The resulting duty cycle per channel is:

$$D = t_{on} / T = 10\ ms / 250\ ms = 0.04\ \ (4\%)$$

Applying the duty cycle to the peak current draw, the time-averaged current consumption is:

$$I_{avg,channel} = I_{peak} \times D = 40\ mA \times 0.04 = 1.6\ mA$$

Converting to charge consumption per second per channel:

$$Q_{per\ second} = I_{avg,channel} / 3600 = 1.6\ mA / 3600\ s/hr = 4.4 \times 10^{-4}\ mAh/s$$

Under a conservative worst-case assumption of continuous sequential stimulation across all three channels simultaneously, the total charge consumed per hour is given by:

$$Q_{per\ hour} = 3 \times I_{peak} \times D \times 1\ hr = 3 \times 40\ mA \times D \times 1\ hr$$

At the nominal 4% duty cycle (10 ms pulse width, 250 ms inter-stimulus interval):

$$Q_{per\ hour} = 3 \times 40\ mA \times 0.04 = 4.8\ mAh/hr$$

The current prototype is equipped with a rechargeable battery of capacity C_{batt} = 190 mAh. Under the worst-case continuous stimulation assumption across all three channels (D = 100%, $Q_{per\ hour}$ = 120 mAh/hr), the theoretical battery life is:

$$t_{battery} = C_{batt} / Q_{per\ hour} = 190\ mAh / 120\ mAh/hr = 1.58\ hr\ \ (\sim 1.6\ hr)$$

Under the nominal operating duty cycle of 4% ($Q_{per\ hour}$ = 4.8 mAh/hr), the theoretical battery life extends to:

$$t_{battery} = 190\ mAh / 4.8\ mAh/hr = 39.6\ hr\ \ (\sim 1.6\ days)$$

This estimate assumes uninterrupted continuous stimulation across all three channels, which substantially exceeds realistic usage patterns in tactile language communication, where stimulation is inherently intermittent. The true operational lifetime under normal use conditions is therefore expected to be considerably longer.

The charge consumption figures presented above are specific to the tactile language communication mode characterised in this study. In alerting or notification scenarios, shorter inter-stimulus intervals and higher pulse widths may be required to convey urgency or encode more complex stimulus patterns. As an illustrative comparison, if the pulse width were increased to t_{on} = 50 ms with the same 250 ms interval, the duty cycle would increase to 20%, yielding a three-channel charge consumption of 24.0 mAh/hr and a corresponding battery life of approximately 7.9 hours. Supplementary Table 7 summarises the estimated charge consumption and battery life across a range of representative stimulation duty cycles for reference.

Duty Cycle (%)	Pulse Width at 250 ms interval (ms)	Charge Consumed per Hour (mAh/hr)	Estimated Battery Life (hr)
4	10	4.8	~39.6 (~1.6 days)
10	25	12.0	~15.8
20	50	24.0	~7.9
40	100	48.0	~4.0
100	250 (continuous)	120.0	~1.6

Supplementary Table 7. Estimated battery life as a function of stimulation duty cycle for the three-channel haptic glove system (C_batt = 190 mAh, I_peak = 40 mA per channel).

Air Permeability (Breathability)

To quantitatively assess the breathability of the haptic textile and its suitability for prolonged skin contact, air permeability measurements were conducted in accordance with ASTM D737 (Standard Test Method for Air Permeability of Textile Fabrics). Three fabric configurations were evaluated: plain knit (standard single jersey), rib knit (double jersey), and rib knit with embedded haptic E-yarn (see Supplementary Figure 25).

As presented in Supplementary Figure 26, the rib knit structure with integrated haptic E-yarn exhibited the highest air permeability among all tested configurations, recording 887.6 mms^{-1}. This represents an increase of approximately 34.6% relative to the standard rib knit structure (659.2 mms^{-1}) and 27.1% relative to the plain knit structure (698.2 mms^{-1}). The enhanced airflow in the integrated structure is attributed to the embedded components, which locally stretch the surrounding knitted stitches and create more open, permeable regions within the fabric architecture. The high measurement consistency across all samples (standard deviation ~4 - 5.5 mms^{-1}) confirms the reliability of these results. These findings demonstrate that the integration of the haptic E-yarn does not compromise breathability; on the contrary, it measurably improves airflow relative to standard knitted structures, which may reduce the potential for localised sweating during extended wear.

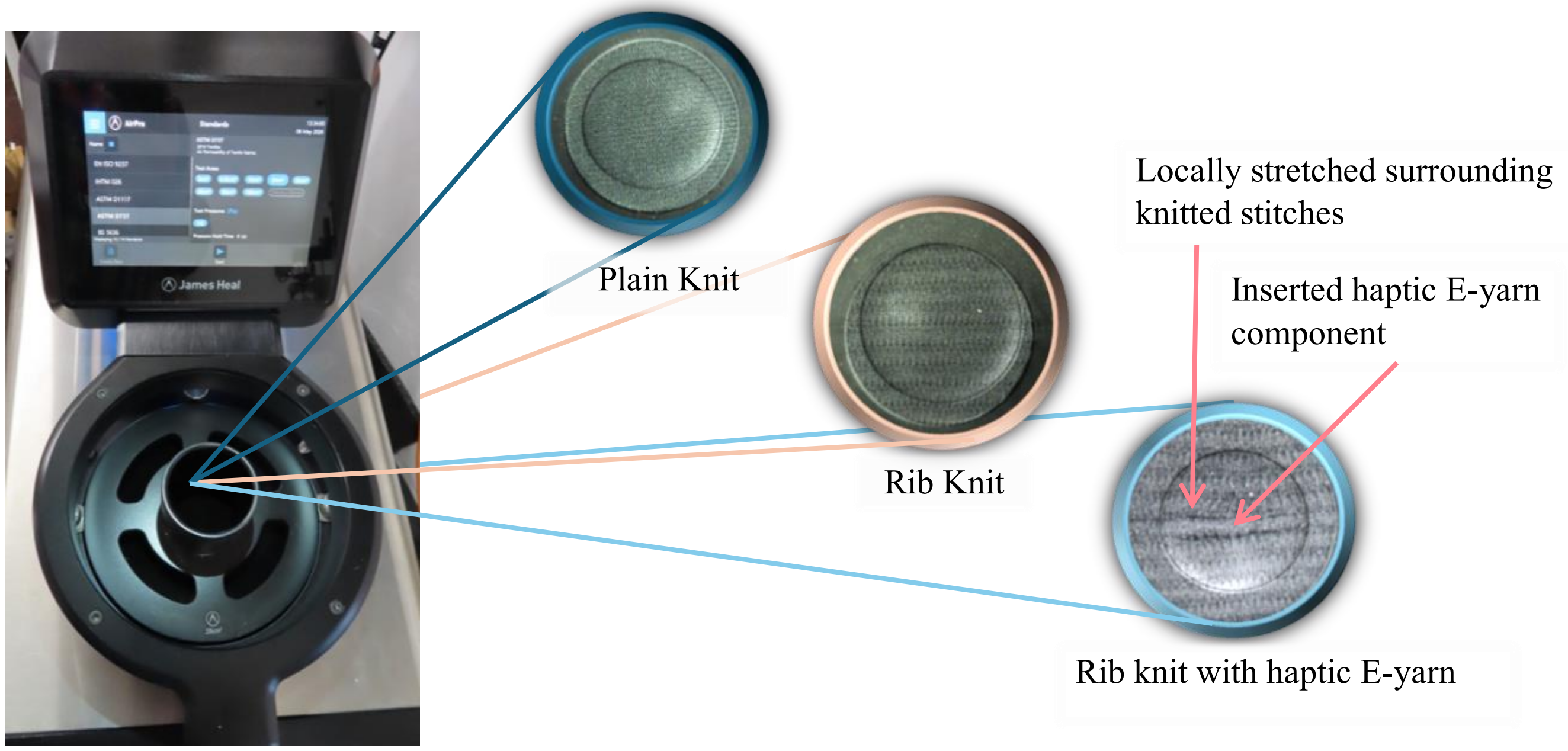

Supplementary Figure 25: Air permeability tester (AirPro, James Heal, Halifax, United Kingdom) and the tested samples.

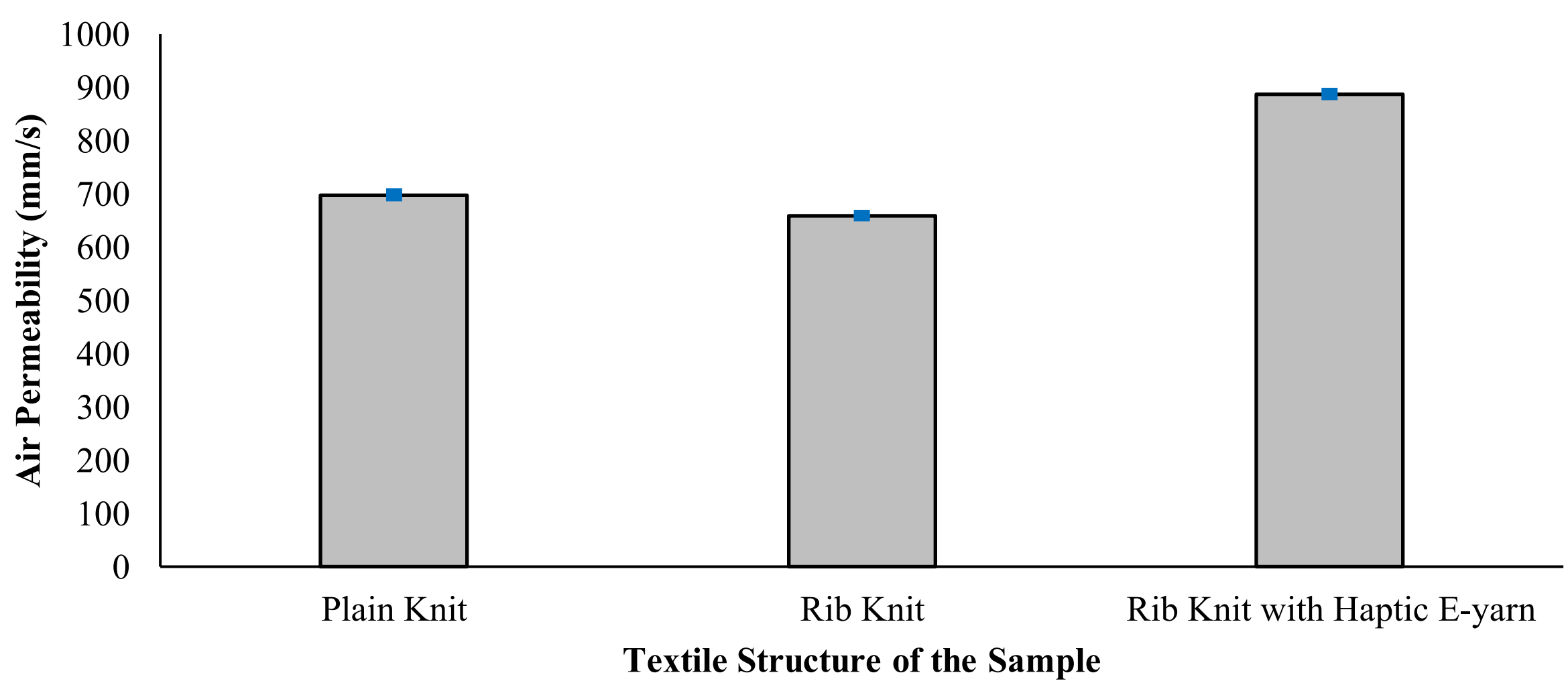

Supplementary Figure 26: Comparison of air permeability in different knitted textile structures and knitted structures with embedded haptic E-yarns.

Bending Stiffness (Drapeability)

Fabric bending stiffness was characterised in accordance with ASTM D1388 using the Shirley cantilever method (using Taber Fabric Stiffness Tester) (see Supplementary Figure 27). The bending modulus values for the three fabric configurations are presented in Supplementary Figure 28.

The plain knit fabric exhibited the lowest bending modulus (0.032 kg/cm^2), indicating the highest structural flexibility. The rib knit fabric exhibited a higher bending modulus (0.052 kg/cm^2 attributable to its more complex double jersey geometry. The rib knit structure with embedded haptic E-yarn (excluding the encapsulated component segment) exhibited the highest bending modulus (0.08 kg/cm^2), representing a 2.5-fold increase relative to the plain knit baseline. This increase in stiffness is attributable to the multi-material composition of the haptic E-yarn - incorporating conductive Litz wires and Vectran fibres within a braided structure - which restricts fabric deformation and enhances structural rigidity. Standard deviation values across five replicate measurements were low (STDV-P: 0.003--0.009), confirming the consistency of the bending behaviour.

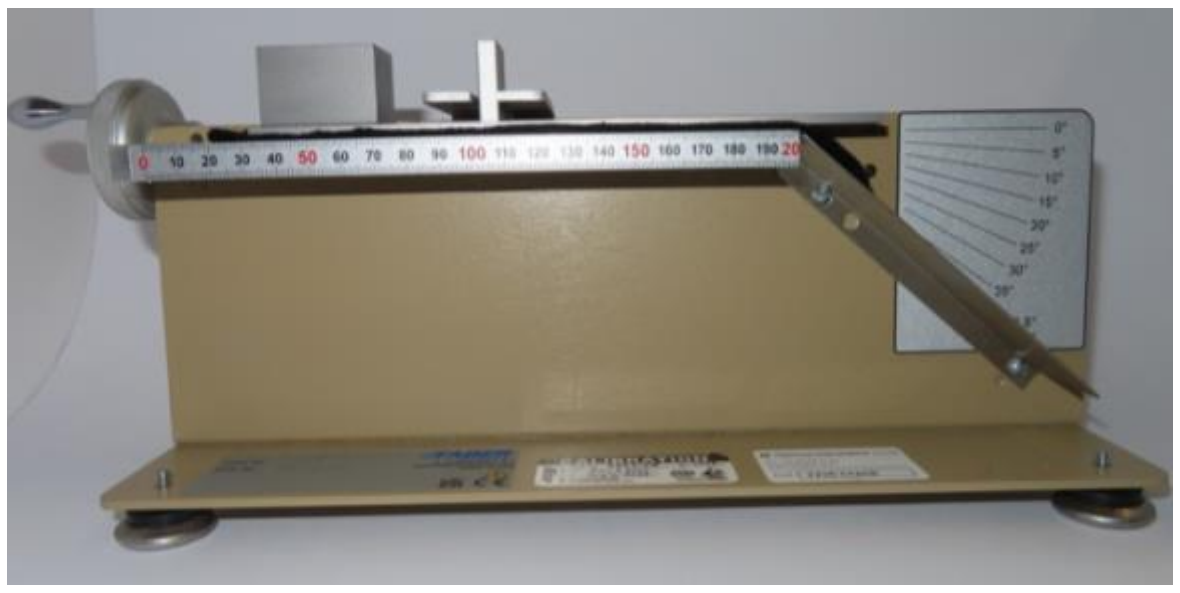

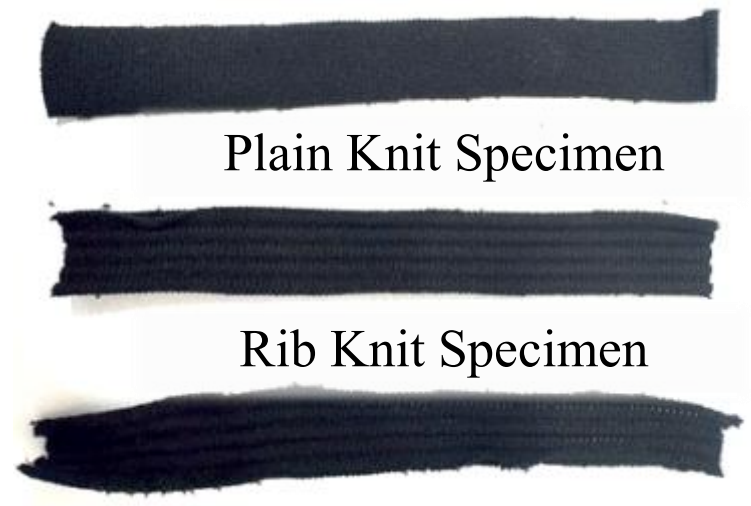

Rib Knit Specimen with embedded non-encapsulated segment of the haptic E-yarn

Supplementary Figure 27: Taber Fabric Stiffness Tester and the tested specimens.

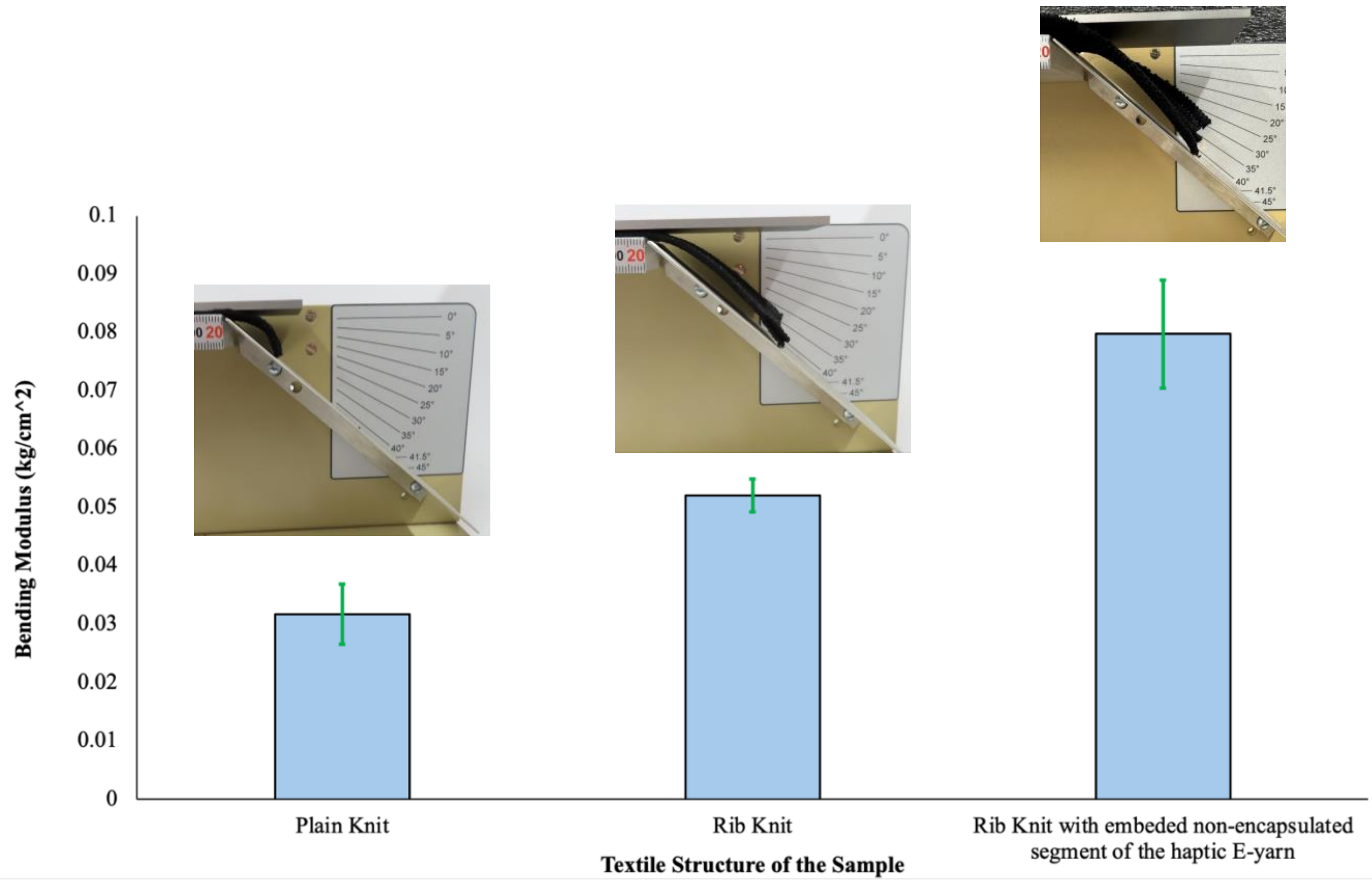

Supplementary Figure 28: Comparison of bending stiffness in different knitted textile structures and knitted structures with embedded haptic E-yarns.
